# Peptide stapling by late-stage Suzuki–Miyaura cross-coupling

**DOI:** 10.3762/bjoc.18.1

**Published:** 2022-01-03

**Authors:** Hendrik Gruß, Rebecca C Feiner, Ridhiwan Mseya, David C Schröder, Michał Jewgiński, Kristian M Müller, Rafał Latajka, Antoine Marion, Norbert Sewald

**Affiliations:** 1 Department of Chemistry, Bielefeld University, Universitätsstr. 25, 33615 Bielefeld, Germany; 2 Department of Technology, Bielefeld University, Universitätsstr. 25, 33615 Bielefeld, Germany; 3 Department of Chemistry, Middle East Technical University, 06800, Ankara, Turkey; 4 Department of Bioorganic Chemistry, Wrocław University of Science and Technology, Wybrzeze Wyspianskiego 27, 50-370 Wrocław, Poland

**Keywords:** accelerated molecular dynamics, halotryptophan, intrinsically disordered peptides, late-stage diversification, macrocyclisation, molecular dynamics, stapled peptides, Suzuki–Miyaura cross-coupling

## Abstract

The development of peptide stapling techniques to stabilise α-helical secondary structure motifs of peptides led to the design of modulators of protein–protein interactions, which had been considered undruggable for a long time. We disclose a novel approach towards peptide stapling utilising macrocyclisation by late-stage Suzuki–Miyaura cross-coupling of bromotryptophan-containing peptides of the catenin-binding domain of axin. Optimisation of the linker length in order to find a compromise between both sufficient linker rigidity and flexibility resulted in a peptide with an increased α-helicity and enhanced binding affinity to its native binding partner β-catenin. An increased proteolytic stability against proteinase K has been demonstrated.

## Introduction

Peptide cyclisation emerged as a popular approach to limit conformational mobility in order to enhance the binding affinity towards a biological target. Moreover, cyclic peptides are more stable against proteolytic digestion and can provide an improved membrane permeability [1–3]. Hence, peptide-based drugs became of high interest because of their high selectivity combined with low toxicity. Cross-linking of side chain residues results in constrained conformations and can be used to stabilise α-helical secondary structures. This technique is called peptide stapling and the most prominent methodology was developed by the groups of Grubbs and Verdine using ring-closing metathesis (RCM) [4–6]. The optimised protocol for these so-called hydrocarbon-stapled peptides uses α-methyl-, α-alkenylglycines in a distance of *i*, *i* + 3/*i* + 4 for one helix turn or *i*, *i* + 7 for two helix turns, respectively, followed by Ru-catalysed cross-linking [7]. By this robust and reliable approach, a library of stapled peptides was generated influencing diverse α-helical dominated protein–protein interactions (PPI) spanning pathways involved in cancer, infectious diseases, metabolic diseases and neurological disorders [8], which had been considered undruggable for a long time due to their large contact area and shallow surface [9]. Since then, many other reactions have been investigated for macrocyclisation with the objective of peptide stapling [10] including lactam- [11–12], disulfide- [13], thioether- [14–20], triazole- [21–22], oxime- [23] and hydrazone formation [24] as well as multicomponent reactions such as the Ugi- or Petasis reaction [25–32]. The content of helicity can moreover be changed by the introduction of a photo-switchable azobenzene staple [33–34]. Moreover, it has been shown that Pd-mediated cross-couplings can be successfully employed in the generation of cyclic and conformationally stabilised peptides. The groups of Buchwald, Pentelute, and Ackermann pioneered the development of Pd-mediated arylation chemistry of biomolecules [35–37]. The approaches by Buchwald and Pentelute are suitable for selective, bioorthogonal labelling of cysteine- [38–40] and lysine-containing [41–42] peptides and proteins using stochiometric amounts of pre-formed Pd(II)-aryl complexes. They can further be applied for peptide macrocyclisation of the two above mentioned side chain residues of the natural amino acids. However, these Pd-mediated stapling reactions were performed only on an analytical scale and the secondary structures of the cyclic peptides were not studied. Since tryptophan has only an incidence of about 1% in proteins, but is highly conserved in binding sites on protein surfaces mediating PPI [43], it is an attractive target for the development of selective diversifications. C–H activation of the indole C^2^ position by Pd-catalysis allows both selective arylation [44–48] and formation of macrocycles [49]. The macrocyclisation technique by tryptophan C^2^–H activation has been further improved showing structurally constrained peptides bearing a side chain connection of tryptophan and phenylalanine or tyrosine [50]. Moreover, a similar Pd-mediated approach for C(sp^3^)–H activation of phthaloyl-protected *N*-terminal alanine was also used for macrocyclisation in peptide stapling [51–52].

Besides addressing the indole C^2^, the regioselective enzymatic halogenation at C5, C6, or C7 using FAD-dependent tryptophan halogenases opens a broad area of Pd-catalysed late-stage diversifications [53–55]. It has been proven that Pd-catalysed cross-couplings are very versatile tools for selective and bioorthogonal modifications of haloindoles, halotryptophans and halotryptophan-containing peptides as well as natural products [56–70]. Additionally, halotryptophans were incorporated in pentapeptides as building blocks for macrocyclisation by Suzuki–Miyaura cross-coupling (SMC) aiming at the preparation of bicyclic peptides [71]. Recently, intramolecular SMC has been successfully applied to side chain-to-tail cyclisation between halotryptophans and boronic acids resulting in RGD peptides with high affinity towards integrin α_V_β_3_, good selectivity and high plasma stability [72].

## Results and Discussion

### Design and synthesis of SMC stapled peptides

**Scheme 1:**
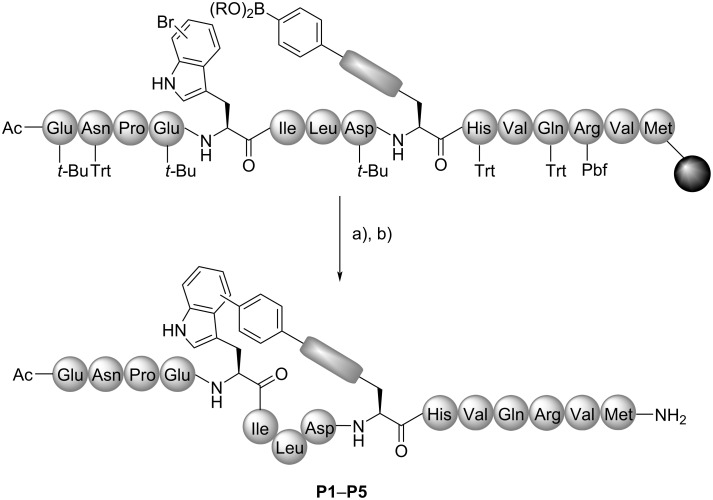
Synthesis of SMC stapled axin CBD peptides. Reaction conditions: (a) Pd_2_(dba)_3_, sSPhos, KF, DME/EtOH/H_2_O 9:9:2, 120 °C, µwave, 30 min; (b) TFA/TIS/H_2_O 95:2.5:2.5, DTT, phenol, 2 × 2 h. B(OR)_2_ = B(OH)_2_, B(pin), pin = pinacolato, *t*-Bu = *tert*-butyl, Trt = trityl, Pbf = pentamethyldihydrobenzofuran-5-sulfonyl.

The intramolecular SMC was envisaged as a novel approach towards one-component peptide stapling by side chain cross-linking of bromotryptophan and an organoboron moiety. Bromotryptophans are accessible by enzymatic bromination utilising cross-linked enzyme aggregates (combiCLEAs) containing an FAD-dependent tryptophan halogenase, a flavin reductase and an alcohol dehydrogenase [73–74]. For this purpose, tryptophan halogenases RebH and Thal were applied for the generation of ʟ-7-bromo- and ʟ-6-bromotryptophan, respectively. As a peptide sequence, we chose the β-catenin-binding domain (CBD) of axin as a benchmark system (PDB ID 1QZ7) [75]. Axin is a scaffold protein playing an essential part in the destruction complex for β-catenin labelling in the canonical Wnt signalling. Loss-of-function mutations in this pathway lead to a dysregulated signal transduction causing cancer [75–76]. All-hydrocarbon stapled peptides comprising amino acids 467 to 481 of the axin CBD had been studied in the group of Verdine and evaluation of optimised staple positions at amino acids 471 (*i*) and 475 (*i* + 4) resulted in enhanced helicity and binding affinity to β-catenin, e.g., for peptide **StAx-3** [77]. Following the **StAx-3** peptide, we designed peptides including bromotryptophan in *i*-position and an organoboron containing side chain in the *i* + 4-position. The peptides were synthesised on Rink amide resin by solid-phase peptide synthesis (SPPS) with Fmoc/*t*-Bu strategy followed by on-resin SMC. For the cross-coupling, a modified protocol by Planas and co-workers was used [78]. Pd_2_(dba)_3_ was employed as the Pd source together with the water-soluble Buchwald ligand sulfonated SPhos (sSPhos) and potassium fluoride as a base. The reaction was performed in a solvent mixture of dimethoxyethane, ethanol and water (DME/EtOH/H_2_O 9:9:2) at 120 °C under microwave irradiation for 30 min (Scheme 1) [78].

**Scheme 2:**
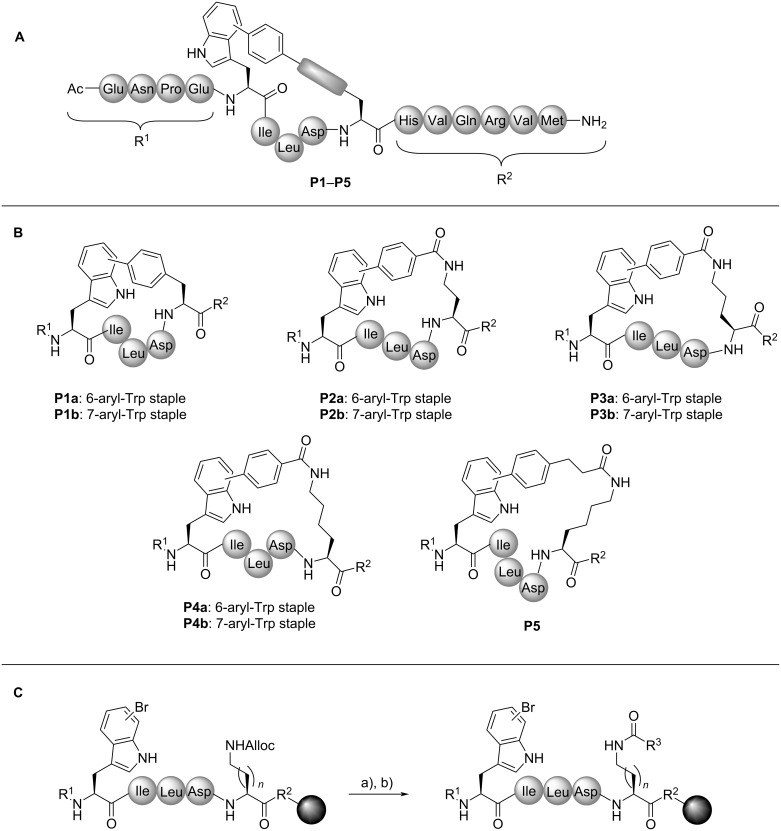
Overview of the different cross-linkages obtained by intramolecular SMC. A) General structure of SMC stapled axin CBD peptides. B) Overview of different cross-links: For peptides **P1** to **P4**, 6- and 7-aryl-Trp staples were synthesised, peptide **P5** was only generated with 7-aryl-Trp staple. C) On-resin modification of amines (*n* = 1: Dab, *n* = 2: Orn, *n* = 3: Lys) in *i* + 4 position to obtain organoboron side chains with increased linker flexibility. Reaction conditions: (a) Pd(PPh_3_)_4_, morpholine in DCM, 2 × 20 min; (b) carboxylic acid, HATU, DIEA in DMF, 1 h. In panel C), amino acids in R^1^ and R^2^ are with protecting groups and R^2^ is resin-bound (Rink amide resin); R^3^ = 4-phenylboronic acid or (4-ethylphenyl)boronic acid (**P2**–**P5**).

The studies were initiated with a macrocyclisation between a 7-bromotryptophan and a 4-pinacolatoborono phenylalanine side chain to generate the cyclic peptide **P1b** (Scheme 2A and B). The intramolecular SMC took place with full consumption of the starting material and without formation of an intermolecular macrocyclisation product, which was confirmed by MALDI-ToF-MS of the crude reaction mixture after test cleavage (see Supporting Information File 1, Figure S1). However, deboronation and dehalogenation were observed as side reactions to some extent as well as oxidation, most likely of methionine (Met) [79]. The oxidation could be minimised by improved cleavage conditions under argon. Replacing sSPhos by tri(*o*-tolyl)phosphine (P(*o*-Tol)_3_), that had successfully been applied for peptide cyclisation by on-resin SMC [78,80], led to incomplete conversion.

The cyclisation of the same peptide with the regioisomer 6- instead of 7-bromotryptophan yielded the expected stapled peptide **P1a**. Next, the secondary structures of the two stapled peptides **P1a** and **P1b** were investigated by CD spectroscopy. Unfortunately, both peptides did not show enhanced helical conformation in water. To get more information about the structure, the spectra were also recorded in a mixture of 2,2,2-trifluoroethanol (TFE)/water 4:1 since TFE promotes the formation of helices [81]. Interestingly, the helical structure could be slightly enhanced but this effect was not as pronounced as for the linear parent peptide aAxWt in TFE/water 4:1 (see Supporting Information File 1, Figure S4).

As a conclusion from those experiments, it was hypothesised that the linker might be too rigid resulting in a distorted structure, which has also been previously reported for thioether cross-linked cysteines bearing a biphenyl template within the staple [20]. Hence, a linker with a higher degree of flexibility was designed. This goal was achieved by a modification of amine-containing amino acids in the *i* + 4 position through coupling to 4-carboxyphenylboronic acid, followed by intramolecular SMC. Different linker lengths were achieved by introducing ʟ-2,4-diaminobutyric acid (Dab), ornithine (Orn), or lysine (Lys). Utilising the Alloc protecting group allowed the coupling of 4-carboxyphenylboronic acid once the linear sequence had been synthesised (Scheme 2C). The intramolecular SMC between 6- or 7-bromotryptophan and the boronic acid afforded the stapled peptides **P2** to **P4** with complete conversion (Scheme 2B). LC–MS analyses revealed broadened or two signals for peptides **P1** to **P4**, which were inseparable by preparative RP-HPLC purification (see Supporting Information File 1). The presence of more than one isomer may be due to the co-existence of diastereomers, i.e. *cis*/*trans* isomers or conformers with a high interconversion barrier. For complestatin-based macrocyclic peptides, the existence of biaryl atropisomers caused by the indole of tryptophan has been reported [82]. Recently, the occurrence of isomers was also observed in our group for SMC cyclised RGD peptides. It could be proven that an isomerisation is not caused by the cross-coupling but by the presence of stable isomers/conformers. Molecular dynamics (MD) simulations verified the appearance of stable, distinct conformers or atropisomers, which were in accordance with the experimental data [72]. Moreover, the epimerization by the conditions of SMC is unlikely as it has been excluded by total hydrolysis of a late-stage SMC modified RGD peptide [67].

Analysis of the secondary structures of the cyclic peptides **P2**–**P4** by CD spectroscopy also did not show a significantly increased α-helicity in water. Investigation of several derivatives by means of density functional theory (DFT) geometry optimisations indicated that substitution at indole C^6^ tends to induce more significant deformation of the peptide chain compared to substitution at indole C^7^. Moreover, introduction of an additional ethylene unit in the linker suggested a conformation with the highest similarity to the linear reference peptide **P6** (see Supporting Information File 1), thus representing a good compromise between rigidity and preservation of the target secondary structure. Serine in *i*-position and glutamic acid in *i* + 4-position of the linear axin CBD sequence ***a*AxWt** were substituted by tryptophan and lysine, respectively, to have a higher analogy to the lysine modified SMC stapled peptides. Following the indications of DFT calculations, stapled peptide **P5** was synthesised by modification of lysine in the *i* + 4-position with 4-(2-carboxyethyl)phenylboronic acid followed by on-resin SMC (Scheme 2B).

**Figure 1:**
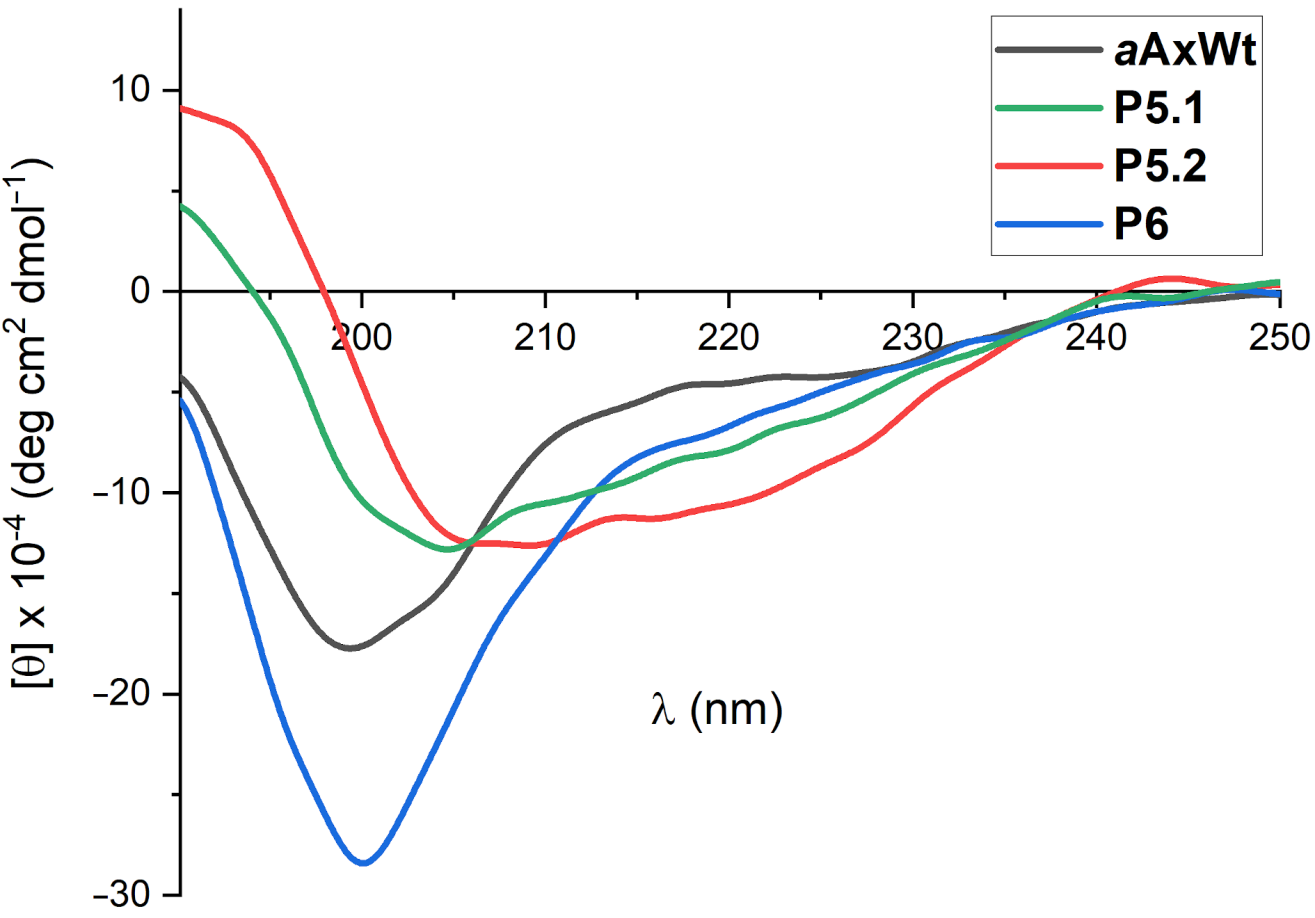
Analysis of the secondary structure by circular dichroism: CD spectra of both isomers of stapled peptide **P5** (**P5.1** and **P5.2**) and linear references (***a*AxWt**, **P6**) (*c* = 100 µM) at 20 °C in water.

LC–MS analysis revealed two isobaric peaks indicating two isomers, which were largely separable by preparative HPLC with the less polar isomer **P5.2** being the major one. The secondary structures of both isomers were investigated by CD spectroscopy and an increased helicity was observed for both peptides. In particular, **P5.2** shows the characteristic signature of an α-helix with the tendency of minima at λ = 208 and 222 nm and a maximum at λ = 190 nm (Figure 1). The CD spectra provided calculated helicities of 9% for ***a*AxWt**, 15% for **P5.1**, and 21% for **P5.2** (see Supporting Information File 1).

### Biological evaluation

**Figure 2:**
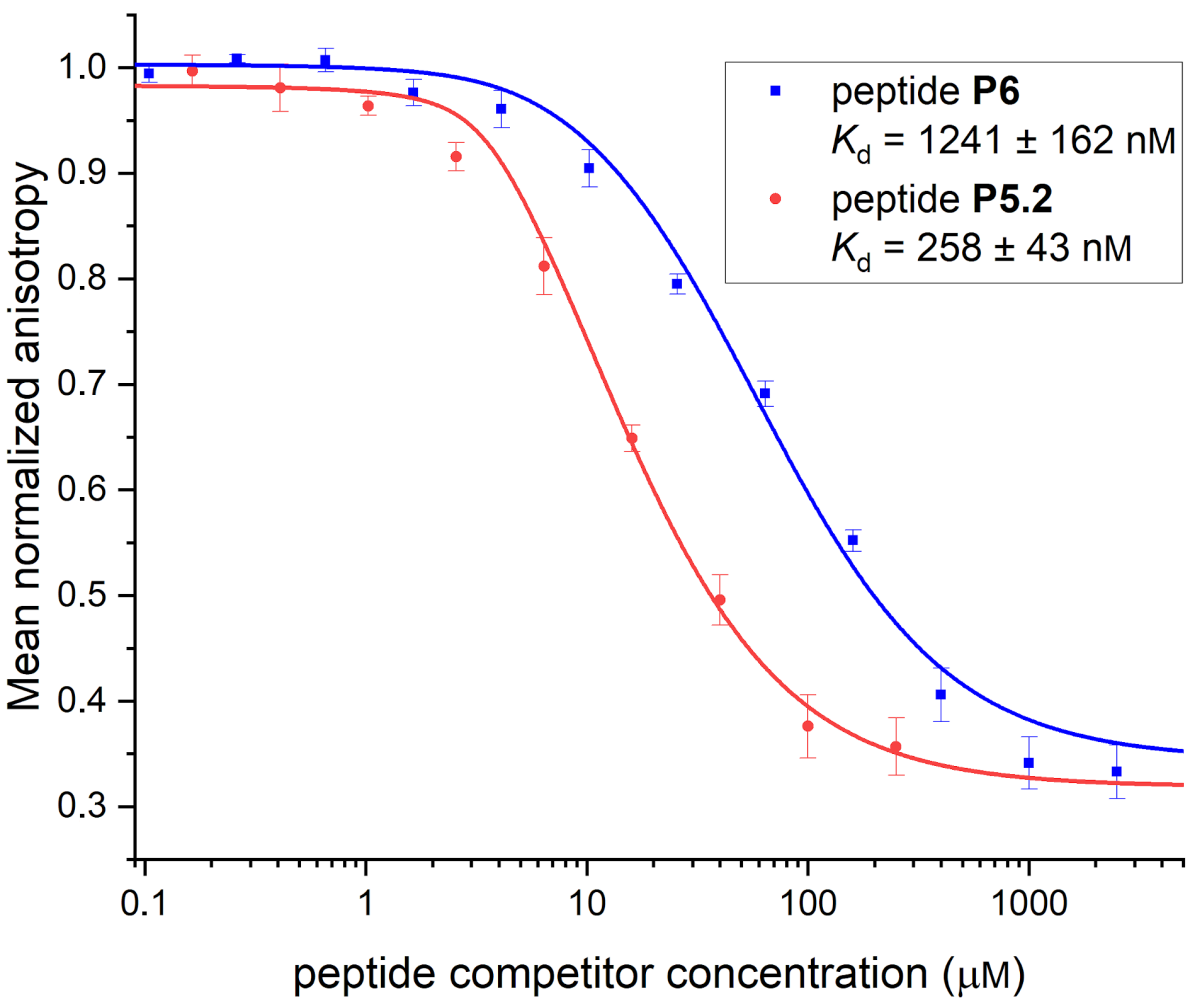
In vitro binding affinities to β-catenin determined by competitive fluorescence polarisation assays.

Competitive fluorescence polarisation (FP) assays were performed to evaluate whether the increased helicity of peptide **P5** also results in an increased binding affinity to its native binding partner β-catenin. The *N*-terminal FITC-labelled RCM-stapled peptide ***f*StAx-3** was synthesised as tracer for this study and its dissociation constant was determined to be 63 ± 6 nM in a direct fluorescence polarisation assay, which is in agreement with previously reported data [77]. Noteworthy, the isomer **P5.2** of stapled peptide **P5** shows an almost five times higher binding affinity (*K*_d_ = 258 ± 43 nM) compared to the linear reference peptide **P6** (*K*_d_ = 1241 ± 162 nM, Figure 2) in the competitive FP assay. Isomer **P5.1** was only isolated in insufficient amounts. Hence, a satisfactorily converging inhibition curve could not be obtained since it requires high inhibitor concentrations. In addition, the wild-type sequence ***a*AxWt** was also tested in the competitive FP assay (*K*_d_ = 1448 ± 204 nM) against its FITC-labelled analogue ***f*AxWt**, which had been determined in a direct assay (*K*_d_ = 1191 ± 182 nM).

**Figure 3:**
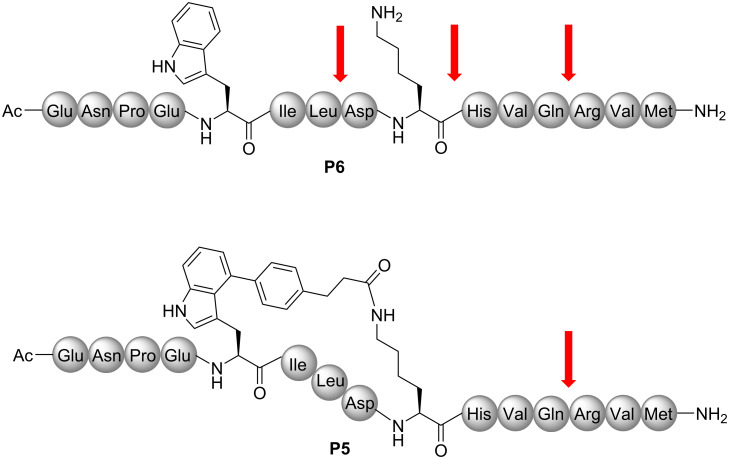
Cleavage sites of Proteinase K digestion indicated by a red arrow.

Finally, the stability of both peptides **P5** and **P6** was tested against proteinase K digestion. Whilst the linear analogue **P6** is cleaved within a period of 120 min to give three fragments, the stapled peptide **P5** allows access to only one of the three cleavage sites: i.e., proteolysis of the Leu–Asp bond within the macrocycle and of the Lys–His bond, which is part of the cross-link, is prevented by the stapling (Figure 3 and Supporting Information File 1, Figures S8 to S10).

### Conformational analysis

The identification of two isomers of **P5** by LC–MS led us investigate the possibility of diastereomers and conformers in the macrocycle. The amide bond in the staple of **P5** is connected to two flexible aliphatic chains and may exist in *cis* and *trans* configuration. The energy difference in the analogue *N*-methylacetamide (NMA) favours the *trans* isomer by about 2.3 kcal mol^−1^, which corresponds to an expected *cis*/*trans* ratio of about 1:44 at 300 K, with an interconversion barrier of 18.7 kcal mol^−1^ [83]. The experimental **P5.1**/**P5.2** ratio is nearly 1:3, suggesting an energy difference of only 0.9 kcal mol^−1^ in favour of **P5.2**. Strain in the macrocycle might be responsible for such slight decrease in the relative energy between the *cis* and *trans* isomers compared to isolated NMA. The presence of diastereomers in the peptide bonds is less likely since the *cis*/*trans* ratio in polypeptides is lower than 1:820 (i.e., an energy difference greater than 4.0 kcal mol^−1^ in favour of the *trans* isomer) [84]. It is, however, currently hopeless to expect catching energy differences as small as that between **P5.1**/**P5.2** by molecular modelling. Instead, we discuss qualitatively the conformational properties of the macrocycle in the two diastereomers of **P5** to determine if conformers may exist with high interconversion barriers, and suggest an assignment based on other experimental observables, namely, the flexibility of the overall peptide and its propensity to form a helical secondary structure. We then proceed to analyse the effect of the staple and sequence variations on the secondary structure of the peptidic backbone of ***a*AxWt**, **P5**, and **P6**.

The conformational preferences of the stapled peptide **P5** and of the linear peptides **P6** and ***a*AxWt** were investigated via extensive accelerated molecular dynamics simulations (aMD) as implemented within the Amber18 program package [85]. The aMD methodology developed by McCammon and co-workers [86] has shown to be a highly effective tool to sample the conformational space of polypeptides made of sequences of 10 to 30 amino acids [87–88] and of macrocycles [89]. Our simulation strategy, mainly adapted from the latter references, made use of 15 independent 700 ns-long aMD simulation runs for each peptide (i.e., a cumulative total of 10.5 μs per peptide) performed with the ff14SB/GAFF [90–91] and TIP4Pew [91] force field parameters for the peptides and water, respectively, as well as specifically derived parameters for non-standard residues of the linker in stapled peptide **P5**. The conformation of the macrocycles was analysed via principal component analysis (PCA) of the non-hydrogen atoms forming the cycle, and the structure of the peptide backbone was investigated via secondary structure analysis and backbone root mean square deviation (RMSD) clustering including amino acids Pro^3^ to Met^15^. Time-averaged analysis was performed on the ensemble of structures obtained from the last 500 ns of each simulation run (i.e., a cumulative total of 7.5 μs per peptide; see Supporting Information File 1 for further methodological details and extended analysis).

**Figure 4:**
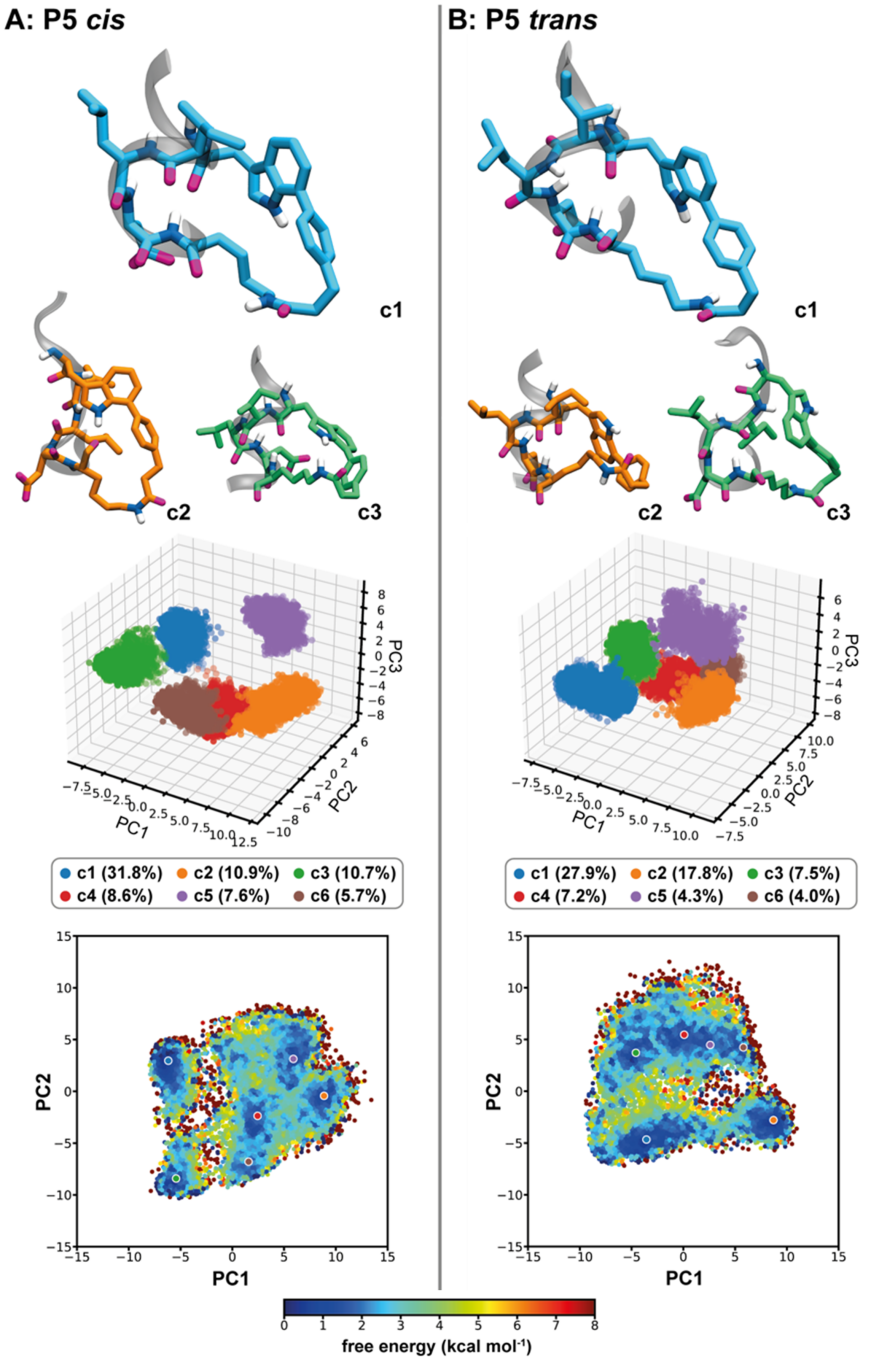
Principal component analysis (PCA) of the macrocycle’s non-hydrogen atoms in the two isomers of **P5**. The upper panel depicts the three main conformations of the macrocycle in **P5** *cis* (column A) and **P5** *trans* (column B) shown in liquorice. Aliphatic hydrogen atoms are hidden and a ribbon representation of the peptidic backbone is shown in grey. Other amino acids in the sequence are not shown in the figure although present in the simulations. The middle panel shows the three-dimensional projection of the six main PCA-based conformational clusters in the space of the first three principal components (PC). The lower panel presents a two-dimensional projection in the plane of the first two PCs. Each point corresponds to a trajectory frame and is coloured according to its corresponding re-weighted relative free energy. Data is calculated on the cumulative last 500 ns of 15 accelerated molecular dynamics runs for each SMC peptide.

Figure 4 summarises the conformational analysis on the macrocycle in the *cis* and *trans* isomer form of **P5**. PCA reveals that the first three principal components (PCs) respectively capture 39.0, 21.3, and 13.4% of the total variance in **P5** *cis*, and 31.2, 25.4, and 9.9% in **P5** *trans*. PCA-based clustering with a minimum distance of 4.0 Å in the three-dimensional space of PC1-3 led to 32 and 38 structural clusters for the *cis* and *trans* isomers, respectively. The first three representative structures are depicted in Figure 4, the first six clusters are projected in the three-dimensional space of PC1-3, and the corresponding representative structures are indicated in the two-dimensional projection in the space of PC1 and PC2, where colouring is made by relative free energy as obtained from Boltzmann reweighting using 10^th^ order Maclaurin series expansion [92–93]. In both isomers, the first three clusters represent about 53% of the total conformational space of the macrocycle. *Cis* and *trans* isomers share a fairly similar main conformation (c1, blue in the figure) with high population (31.8 and 27.9%, respectively). This conformation is stabilised by a hydrogen bond between the CO group of Ile and the NH group of Lys, and triggers the formation of a helical structure in the peptidic portion of the macrocycle. Conformation c2 of the *cis* isomer (10.9%) presents the Leu side chain pointing towards the centre of the macrocycle and leads to a disruption of helicity. The third conformation for this isomer (c3 with 10.7%) is also helical in the peptidic portion of the macrocycle and is similar to c1, with the Trp’s indole group pointing in the other direction. In the *trans* isomer, both conformations are also found, however, with different populations and order. Conformation c2 of **P5** *trans* is helical and resembles c3 of **P5** *cis*, yet with a significantly higher probability of occurrence (17.8%). The non-helical conformation c3 of **P5** *trans* (7.5%) is slightly less populated than the corresponding c2 of **P5** *cis*. The macrocycle of both isomers is found to be rather flexible, forming well-separated conformational clusters in the three-dimensional space of PC1-3. The projection in the two-dimensional space of PC1 and PC2 indicates that the conformers are interconnected with barriers lower than 6 kcal mol^−1^. It is worth noting, that the barriers are likely to be overestimated due to a poor sampling of transition structures compared to local minima. More accurate values would be obtained with methods better adapted for kinetics, such as Markov models (for a general overview see reference [94]). Yet, such low barriers are not sufficient to trap the SMC peptide in conformations that can be separated experimentally at ambient conditions [95] and the analysis therefore rules out the possibility of conformational isomers, within the limits of exhaustivity of our sampling. The *cis*/*trans* conversion barrier is likely to be close to that of isolated NMA, and leads us to conclude that the two isomers isolated experimentally are diastereomers of the amide bond in the staple of **P5**.

**Figure 5:**
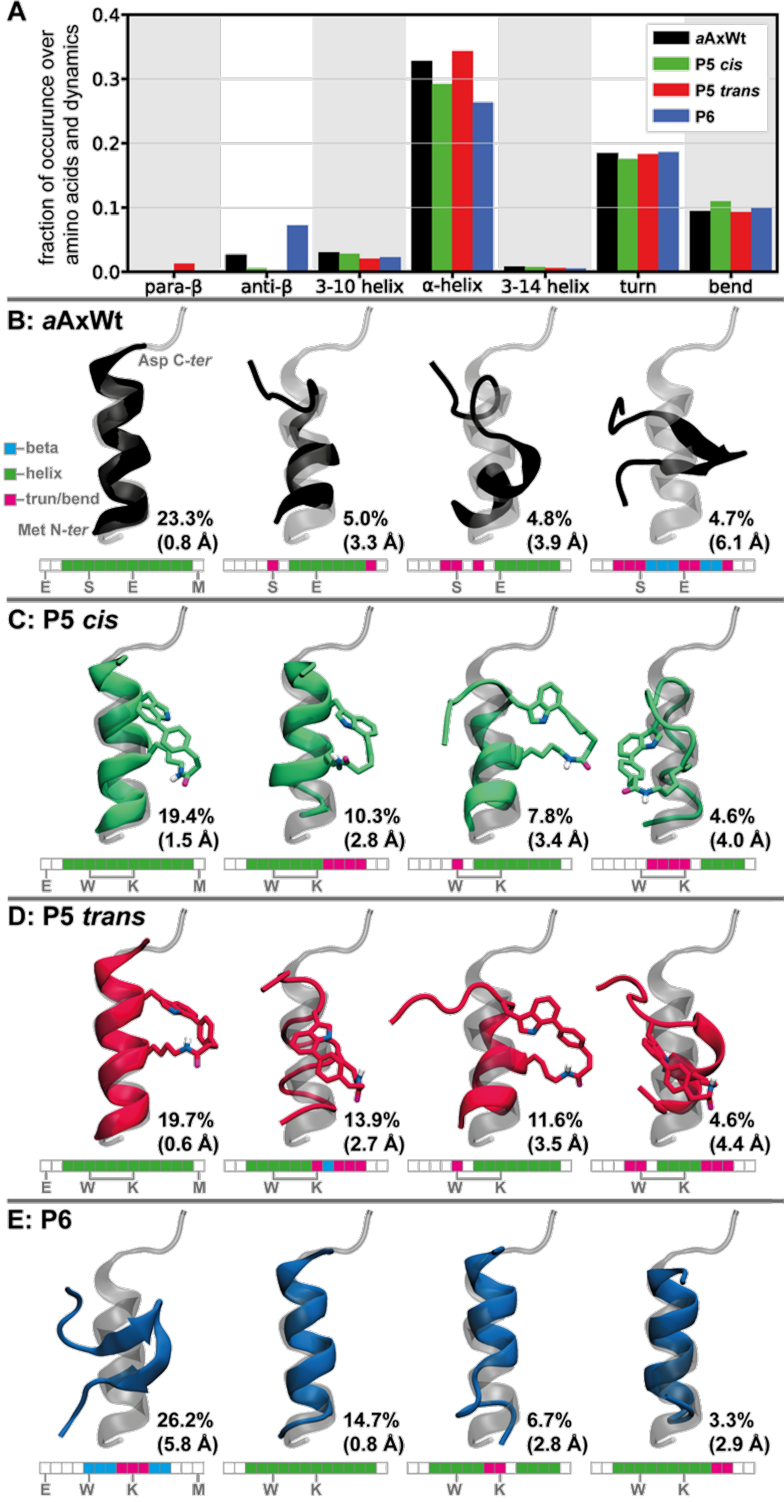
Molecular modelling of the conformational preferences of the SMC stapled peptides **P5** (with *cis* or *trans* amide bond in the staple), and the linear references **P6** and ***a*AxWt** by means of accelerated molecular dynamics (aMD). A. Secondary structure analysis showing the fraction of amino acids found in a specific backbone conformation as normalised over the full sequence and the cumulative last 500 ns of 15 aMD runs for each peptide. B. Results of structural clustering analysis for peptide ***a*AxWt**. The representative structure (black) of the four main structural clusters are depicted superimposed to the active conformation of axin’s binding domain (in transparent grey; PDB ID 1QZ7) [75]. Cluster populations are given next to the respective structure and backbone-atoms root mean square deviation with respect to the active conformation are indicated in parenthesis. RMSD reported and used in clustering were calculated from Pro to Met only. A schematic representation of the secondary structure of each amino acid is given bellow the representative structures as obtained from an average over the whole cluster (beta structures in blue, helices in green, and turn/bend in pink). C–E. Same as panel B for **P5.1** (green), **P5.2** (red), and **P6** (blue), respectively. **P5.1** and **P5.2** differ in the conformation of the amide bond in the staple (*cis* and *trans*, respectively).

The secondary structure analysis of the three peptides ***a*AxWt**, **P5** (*cis* and *trans*) and **P6** is summarized in Figure 5. Figure 5A reports the percentage of amino acids adopting a given secondary structure over the simulation time. All peptides show a significant fraction of amino acids in an α-helical conformation, with a smaller yet substantial propensity to participate in turns and bends. Overall, **P5** *trans* is the most helical peptide, followed by ***a*AxWt**, and **P5** *cis*, and **P6** is significantly less helical than the others, in terms of individual amino acid’s contributions. **P6** stands out with a fairly high fraction of amino acids present in an anti-parallel β-sheet backbone conformation. ***a*AxWt** also shows a small fraction of anti-parallel β-sheet, while **P5** *trans* only shows a marginal percentage of parallel β-structure. The backbone RMSD-based clustering further breaks down the conformational preferences of the three peptides and is summarized in Figure 5B–E showing the representative structures of the first four structural clusters. The main conformation of ***a*AxWt**, **P5** *cis*, and **P5** *trans* is highly populated (23.3, 19.4, and 19.7%, respectively), and shows a full α-helix that closely resembles the active conformation of axin’s binding domain (superimposed in transparent grey). **P6** also forms a similar α-helix with a fairly high probability (14.7%). However, the main conformation of **P6** (26.2%) is found to be formed by two β-sheets linked by a turn at the middle of the sequence. The second and third conformations of ***a*AxWt**, **P5** *cis*, and **P5** *trans* are also significantly helical, with helices formed by at least six consecutive amino acids. The fourth conformation of ***a*AxWt** is a β-structure resembling the main geometry of **P6**. Overall, ***a*AxWt**, **P5** *cis*, and **P5** *trans* form helices made of at least six consecutive amino acids with a cumulative probability of 33.1, 37.5, and 45.2%, respectively. Noteworthy, these percentages are not re-weighted and are, therefore, somewhat biased by the aMD protocol. Yet, trends should be qualitatively captured by the analysis, which correlates fairly well with the experimental results in Table S1 (Supporting Information File 1).

The mutations from ***a*AxWt** to **P6** result in a significant change in conformational preferences and the probability of stable β-structures in the latter. This observation is consistent with the CD spectrum of **P6** that presents β-sheets features. The staple in **P5** successfully quenches such biologically unfavourable conformation and significantly increases the probability of forming helical structures that closely resemble the active conformation of axin’s binding domain. Both, *cis* and *trans* isomers form α-helices with a high probability, yet the *trans* isomer tends to be more helical than the *cis* variant. The CD spectra of **P5.1** and **P5.2** indicate that the latter has a more helical character, which leads us to speculate that **P5.1** corresponds to the *cis* diastereomer, while **P5.2** presents the amide bond in the staple in a *trans* configuration. Furthermore, analysis of the structural diversity of the two isomers of **P5** indicates that **P5** *cis* (**P5.1**) is more disordered (see Supporting Information File 1, Table S3), which also correlates with a blue-shifted absorption minimum compared to **P5** *trans* (**P5.2**).

In the partially helical structures of ***a*AxWt** (i.e., second and third conformations in Figure 5b), the helix is formed in the second half of the sequence. In **P5** *cis* and **P5** *trans*, however, the second conformation presents the beginning of the sequence with a helical structure, including the amino acids that participate in the macrocycle. While possible, it would be speculative to link this property of the **P5** to its enhanced biological activity. Instead, we find a more likely reason for the greater activity of **P5.2** over that of ***a*AxWt** in analysing the conformational diversity of the two peptides (see Supporting Information File 1, Table S2). In **P5 ***trans* (identified as **P5.2**), 60% of the conformational space is represented by the first 7 structural clusters against 18 for ***a*AxWt**. The latter is, therefore, significantly more flexible and may be found more often in a non-active conformation, including β-structures, compared to **P5.2**. This last observation tends to correlate with a higher binding affinity of **P5.2** over its linear counterparts. Although all peptides can form an α-helix that resembles the active form of axin’s binding domain, **P6** and ***a*AxWt** occur often in other conformations that are rather far from the active one. Overall, when presenting the peptides to the target domain of β-catenin, **P6** and ***a*AxWt** are substantially less likely to be in an active or near-active conformation compared to **P5.2**. In the ensemble of conformations, the fraction of active ones is therefore greater for **P5.2**, which translates into a greater binding affinity measured experimentally.

## Conclusion

In conclusion, suitable reaction conditions were found for the synthesis of stapled peptides by an intramolecular late-stage SMC on resin. The peptide sequences are based on the CBD of axin. Optimisation of the cross-link, guided by DFT geometry optimisation, finally resulted in SMC stapled peptide **P5** showing an increased α-helicity. Compared to the linear analogue **P6**, **P5** revealed a five times higher binding affinity to its native binding partner β-catenin. A proteinase K stability assay demonstrated higher stability of the stapled peptide **P5** against proteolytic digestion because two of the three cleavage sites are blocked by the macrocycle. Accelerated molecular dynamics simulations verified a significantly higher degree of helicity for SMC stapled peptide **P5** compared to the linear analogues **P6** and ***a*AxWt**, which is in accordance with the experimental data obtained from CD and moreover, explains the increased binding affinity to β-catenin as **P5** is more likely to be found in an active conformation.

## Supporting Information

File 1 Details on the amino acid and peptide synthesis, analytical data of the peptides, CD spectroscopy, β-catenin expression and purification, fluorescence polarization assay, proteinase K stability assay, and theoretical methods.
