# Supplementary material for: Peptide stapling by late-stage Suzuki–Miyaura cross-coupling
Source: Beilstein J Org Chem. 2022 Jan 3;18:1–12. doi: 10.3762/bjoc.18.1 (PMC8744458; doi:10.3762/bjoc.18.1)
Supplement: File 1 — Details on the amino acid and peptide synthesis, analytical data of the peptides, CD spectroscopy, β-catenin expression and purification, fluorescence polarization assay, proteinase K stability assay, and theoretical methods. [file Beilstein_J_Org_Chem-18-01-s001.pdf]

BEILSTEIN JOURNAL OF ORGANIC CHEMISTRY

Supporting Information

for

Peptide stapling by late-stage Suzuki–Miyaura cross-coupling

Hendrik Gruß, Rebecca C. Feiner, Ridhiwan Mseya, David C. Schröder,
Michał Jewgiński, Kristian M. Müller, Rafał Latajka, Antoine Marion and Norbert Sewald

Beilstein J. Org. Chem. **2022,** *18,* 1–12. doi:10.3762/bjoc.18.1

Details on the amino acid and peptide synthesis, analytical data of the peptides, CD spectroscopy, β-catenin expression and purification, fluorescence polarization assay, proteinase K stability assay, and theoretical methods

License and Terms: This is a supporting information file under the terms of the Creative Commons Attribution License (https://creativecommons.org/licenses/by/4.0). Please note that the reuse, redistribution and reproduction in particular requires that the author(s) and source are credited and that individual graphics may be subject to special legal provisions.
The license is subject to the *Beilstein Journal of Organic Chemistry* terms and conditions: (https://www.beilstein-journals.org/bjoc/terms)

Table of contents

General information S1
GP1: General procedure for enzymatic bromination of tryptophan S5
GP2: General procedure for Fmoc-protection of bromotryptophan S5
Synthesis of *N*$^{\alpha}$-Fmoc-4-pinacolatoborono-L-phenylalanine S6
Peptide synthesis S8
GP3: General procedure for on-resin Suzuki–Miyaura cross-coupling S10
GP4: General procedure for on-resin ring-closing metathesis S10
Example MALDI TOF MS of crude peptide P1b S11
Analytical data of peptides S12
Circular dichroism S31
β-Catenin expression and purification S34
Direct fluorescence polarisation assay S35
Competitive fluorescence polarisation assay S36
Proteinase K stability assays S38
Density functional theory (DFT) calculations S41
Molecular dynamics-based conformational analysis S45
References S47

General information

Standard chemicals and solvents were obtained from commercial suppliers in highest purity suitable for analytical applications (p. a.). Sodium 2′-dicyclohexylphosphino-2,6-dimethoxy-1,1′-biphenyl-3-sulfonate hydrate (sSPhos), Grubbs 1st generation catalyst and Fmoc-(*S*)-2-(4-pentenyl)alanine (Fmoc-S_5-OH) were purchased from Sigma-Aldrich, Tris(dibenzylideneacetone)dipalladium(0) ($Pd_2(dba)_2$) from Alfa Aesar, boronic acids from Acros Organics or Alfa Aesar, Rink Amide AM resin, Fmoc-protected amino acids, TBTU and HATU from Iris Biotech or Carbolution Chemicals and FITC from AppliChem and were used without further purification. The plasmid vector encoding alcohol dehydrogenase, pET21-ADH, was a gift from Prof. Dr. Werner Hummel, pCIBhis-PrnF encoding the flavin reductase was donated by Prof. Dr. Karl-Heinz van Pée and the plasmid M57 pPET28a-TEV-full-length human β-catenin was a gift from Randall Moon (Addgene plasmid # 17198). The water used for reactions, workups and purifications was purified by a Millipore Milli-Q biocel device. Solvents for cross-coupling reactions were degassed by freeze-pump-thaw (3 cycles). DCM was dried over CaH_2 and distilled and DMF over 4 Å molecular sieve. DMF was degassed by a stream of argon for 60 min. Silica gel 60 (Macherey-Nagel) with a particle size of 0.04-0.063 mm (230-400 mesh) was used for column chromatography. Fractions were analysed by TLC

with silica gel 60-coated aluminium sheets (Macherey-Nagel) and spots visualised by irradiation (λ = 254 nm) or $KMNO_4$ staining solution.

Abbreviations

AA: amino acid, CV: column volume, DCE: 1,2-dichloroethane, DCM: dichloromethane, DFT: density functional theory, DIEA: *N*,*N*-diisopropylethylamine = Hünig base, DHB: 2,5-dihydroxybenzoic acid, DME: 1,2-dimethoxyethane, DMF: dimethylformamide, EtOH: ethanol, FITC: fluorescein 5-isothiocyanate, HATU: *O*-(7-azabenzotriazol-1-yl)-*N*,*N*,*N*′,*N*′-tetramethyluronium hexafluorophosphate, HRMS: high resolution mass spectrometry, MD: molecular dynamics, MeOH: methanol, MTBE: methyl-*tert*-butylether, NMP: *N*-methyl-2-pyrrolidon, NP: normal phase, ^{i}PrOH: *iso*-propanol, prop.: proposal, RCM: ring-closing metathesis, RP: reversed phase, r.t.: room temperature, SMC: Suzuki-Miyaura cross-coupling, TBTU: *O*-benzotriazol-1-yl-*N*,*N*,*N*',*N*'-tetramethyluronium tetrafluoroborate, TFA: trifluoroacetic acid, TFE: 2,2,2-trifluoroethanol.

RP-HPLC(-MS)

Analytical LC-MS was performed on an Agilent 6220 TOF-MS with a Dual ESI-source, 1200 HPLC system with autosampler, degasser, binary pump, column oven, diode array detector and a Hypersil Gold C_{18} column (1.9 µm, 50 × 2.1 mm) with a gradient (in 11 min from 0% B to 98% B, back to 0% B in 0.5 min, total run time 15 min) at a flow rate of 300 µL/min and column oven temperature of 40°C. HPLC solvent A consists of 94.9% water, 5% acetonitrile and 0.1% formic acid, solvent B of 5% water, 94.9% acetonitrile and 0.1% formic acid. ESI mass spectra were recorded after sample injection via 1200 HPLC system in extended dynamic range mode equipped with a Dual-ESI source, operating with a spray voltage of 2.5 kV. Nitrogen served both as the nebuliser gas and the dry gas. Nitrogen was generated by a nitrogen generator NGM 11.

MALDI-TOF-MS

Dissolved samples were mixed on a steel target with 1 µL of matrix solution (50 mg mL^{-1} 2,5-DHB in 50% ACN containing 0,1% TFA). MALDI-TOF mass spectra were recorded with a Ultraflex MALDI-TOF/TOF mass spectrometer (Bruker Daltonik, Bremen, Germany) operated in reflectron positive mode. Ionisation was achieved using an LTB nitrogen laser MNL 200 (337 nm beam wavelength, 50 Hz repetition rate). Maximum resolution achieved with this instrument is approx. 20,000. Calibration was conducted with PEG 400-1200 and FlexControl 3.0 and FlexAnalysis 3.4 (Bruker Daltonik) were used for recording and processing.

ESI MS

Nano-ESI mass spectra were recorded using an Esquire 3000 ion trap mass spectrometer (Bruker Daltonik GmbH, Bremen, Germany) equipped with a nano-ESI source. Samples were dissolved in acetonitrile and introduced by static nano-ESI using in-house pulled glass emitters. Nitrogen served both as nebuliser gas and dry gas. Nitrogen was generated by a Bruker nitrogen generator NGM 11. Helium served as cooling gas for the ion trap. The mass axis was externally calibrated with ESI-L Tuning Mix (Agilent Technologies, Santa Clara, CA, USA) as calibration standard. The spectra were recorded with the Bruker Daltonik esquireNT 5.2 esquireControl software by the accumulation and averaging of several single spectra. DataAnalysis™ software 3.4 was used for processing the spectra.

High-resolution MS

ESI accurate mass measurements were performed using either an Agilent 6220 time-of-flight mass spectrometer (Agilent Technologies, Santa Clara, CA, USA) or a Q-IMS-TOF mass spectrometer Synapt G2Si (WatersGmbH, Manchester, UK).

Agilent 6220 TOF mass spectrometer

ESI accurate mass measurements are acquired in extended dynamic range mode equipped with a Dual-ESI source, operating with a spray voltage of 2.5 kV. Nitrogen served both as the nebuliser gas and the dry gas. Nitrogen was generated by a nitrogen generator NGM 11. Samples are introduced with a 1200 HPLC system consisting of an autosampler, degasser, binary pump, column oven and diode array detector (Agilent Technologies, Santa Clara, CA, USA) using a C18 Hypersil Gold column (length: 50 mm, diameter: 2.1 mm, particle size: 1,9 µm) with a short gradient (in 4 min from 0% B to 98% B, back to 0% B in 0.2 min, total run time 7.5 min) at a flow rate of 250 µL/min and column oven temperature of 40°C. HPLC solvent A consists of 94.9% water, 5% acetonitrile and 0.1% formic acid, solvent B of 5% water, 94.9% acetonitrile and 0.1% formic acid. The mass axis was externally calibrated with ESI-L Tuning Mix (Agilent Technologies, Santa Clara, CA, USA) as calibration standard. The mass spectra are recorded in both profile and centroid mode with the MassHunter Workstation Acquisition B.04.00 software (Agilent Technologies, Santa Clara, CA, USA). MassHunter Qualitative Analysis B.07.00 software (Agilent Technologies, Santa Clara, CA, USA) was used for processing and averaging of several single spectra.

Q-IMS-TOF mass spectrometer Synapt G2Si

Accurate Mass nano-ESI measurements were performed in resolution mode, interfaced to a nano-ESI ion source. Nitrogen serves both as the nebuliser gas and the dry gas for nano-ESI. Nitrogen is generated by a nitrogen generator NGM 11. Samples were dissolved in mixtures of acetonitrile, water and 0.1% formic acid and introduced by static nano-ESI using in-house pulled glass emitters. The mass axis was internally calibrated with the doubly protonated GluFib ion or the protonated LeuEnk ion as internal calibration standard. Scan accumulation and data processing was performed with MassLynx 4.1 (WatersGmbH, Manchester, UK) on a PC Workstation. The spectra were generated by the accumulation and averaging of several single spectra. Determination of exact masses were performed using centroided data. Drift spectra were visualised and selected with DriftScope v2.8.

Preparative RP-HPLC

Preparative RP-HPLC was performed on a Merck-Hitachi LaChrom system equipped with interface module D-7000, pump L-7150 and detector L-7420 using solvent A (H_2O:ACN:TFA 95:5:0.1) and solvent B (H_2O:ACN:TFA 5:95:0.1) as mobile phase.

Method A: Nucleosil 100-7 C_{18} column (7 µm, 250 × 10 mm) from Macherey-Nagel with a gradient (in 45 min from 0% B to 100% B) at a flow rate of 4 mL/min. Detection took place at a wavelength of 220 nm.

Method B: Hypersil Gold C_{18} column (8 µm, 250 x 21.2 mm) from Thermo-Scientific with a gradient (5 min 40% B, then in 60 min from 40% B to 100% B) at a flow rate of 10 mL/min. Detection took place at a wavelength of 220 nm.

Automated column chromatography

Automated NP-column chromatography on silica gel was partly performed using a Büchi Reveleris X2 chromatography system with UV and ELSD detection.

Method A: FlashPure EcoFlex Silica 12 g column, flow rate 30 mL min^{-1}, solvent A: dichloromethane, solvent B: methanol, gradient (2 min 0% B, 3.2 min 8% B, 6.1 min 9% B, 4.8 min 20% B)

NMR spectroscopy

NMR spectra were recorded on a Bruker Avance III 500 HD (^{1}H: 500 MHz, ^{13}C: 126 MHz). Chemical shifts δ [ppm] are reported relative to residual solvent signal ($CDCl_3$, ^{1}H: 7.26 ppm, ^{13}C: 77.2 ppm; DMSO-d_6, ^{1}H: 2.50 ppm, ^{13}C: 39.5 ppm).

GP1: General procedure for enzymatic bromination of tryptophan

Enzymatic bromination on a preparative scale was performed according to our previously reported procedure using halogenase-PrnF-*RR*-ADH combiCLEAs.[1] The biocatalyst was produced using lysed *E. coli* cells containing overexpressed tryptophan-7-halogenase RebH or tryptophan-6-halogenase ThaI resulting from 1.5 L expression culture. After cell lysis via French-Press (three times), cell debris were spun down and flavin reductase PrnF (2.5 U mL^{-1}) and alcohol dehydrogenase *RR*-ADH (1 U mL^{-1}) were added to the supernatant. Finely grounded ammonium sulphate was added for protein precipitation and incubated at 4 °C for 1 h under permanent inversion. After addition of glutaraldehyde (0.5% w/v for RebH, 1.0% w/v for ThaI), the orange suspension was again incubated at 4 °C for 2 h under permanent inversion. The resulting combiCLEAs were isolated by centrifugation and thoroughly washed with Na_2HPO_4 buffer (100 mM, pH 7.4, 3 × 50 mL). For enzymatic bromination, the combiCLEAs were suspended in the reaction buffer containing L-tryptophan (1 mM for RebH, 0.5 mM for ThaI), Na_2HPO_4 (15 mM), NaBr (30 mM), NAD^+ (0.1 mM), FAD (1 μM) and 5% (v/v) iPrOH at pH 7.4 in a total reaction volume of 1000 mL. Full conversion was usually observed after three days for RebH and eleven days for ThaI. The suspension was filtrated and desalted. For desalting, the filtrate was concentrated up to a volume of about 200 mL and loaded on a RP-C18-column, which was equilibrated with solvent A (water containing 0.1% TFA). The loaded column was washed with solvent A (200 mL) and 7-bromotryptophan eluted using solvent B (methanol containing 0.1% TFA, 300 mL). The elution fraction was concentrated and freeze-dried to provide bromotryptophan·TFA as pale brown solid.

GP2: General procedure for Fmoc-protection of bromotryptophan

Fmoc-Osu (1.2 equiv) was added to a solution of bromotryptophan·TFA (1.0 equiv) and sodium carbonate (2.5 equiv) in a mixture of 1,4-dioxane/water 9:1 (20 mL/mmol bromotryptophan) and stirred for 16 h at room temperature. If necessary, the pH was re-adjusted during the reaction progress with a saturated, aqueous sodium carbonate solution to maintain a pH of 9–10. For workup, brine (50 mL/mmol bromotryptophan) and ethyl acetate (100 mL/mmol bromotryptophan) were added and under stirring, the pH adjusted to three with 5%, aqueous $KHSO_4$-solution. The aqueous layer was extracted with ethyl acetate (3 ×). The combined organic layers were washed with water (3 ×) and dried over sodium sulphate. After removal of the solvent *in vacuo*, the crude product was either purified by column chromatography using a Büchi Reveleris X2 chromatography system (method A) or preparative RP-HPLC (method B).

$N^α$-Fmoc-L-6-bromotryptophan (52.6 mg, 40%) was obtained as colourless solid after purification by preparative RP-HPLC on a 0.25 mmol scale. **^{1}H-NMR** (500 MHz, DMSO-d_6): δ [ppm] = 12.70 (s, 1H), 11.00 (d, J = 2.4 Hz, 1H), 7.88 (d, J = 7.6 Hz, 2H), 7.69 (d, J = 8.3 Hz, 1H), 7.64 (t, J = 8.2 Hz, 2H), 7.54 – 7.51 (m, 2H), 7.44 – 7.37 (m, 2H), 7.34 – 7.25 (m, 2H), 7.20 (d, J = 2.4 Hz, 1H), 7.10 (dd, J = 8.5, 1.8 Hz, 1H), 4.27 – 4.14 (m, 4H), 3.17 (dd, J = 14.6, 4.6 Hz, 1H), 3.01 (dd, J = 14.6, 9.8 Hz, 1H). **^{13}C-NMR** (126 MHz, DMSO-d_6): δ [ppm] = 173.6, 156.0, 143.8, 143.7, 140.7, 137.0, 127.6, 127.1, 126.3, 125.3, 125.2, 124.8, 121.3, 120.1, 120.0, 114.0, 113.7, 110.7, 65.7, 55.0, 46.6, 26.7. **MS** (ESI, negative ions): found m/z = 502.8 [M(^{79}Br)-H]$^-$, 504.8 [M(^{81}Br)-H]$^-$; calcd. m/z = 503.1 [M(^{79}Br)-H]$^-$, 505.1 [M(^{81}Br)-H]$^-$. The spectroscopic data are in accordance with literature.[2]

$N^α$-Fmoc-L-7-bromotryptophan (100.0 mg, 87%) was obtained as colourless solid after purification by Büchi Reveleris X2 chromatography system on a 0.23 mmol scale. **^{1}H-NMR** (500 MHz, DMSO-d_6): δ [ppm] = 12.74 (s, 1H), 11.12 (d, ^3J = 2.4 Hz, 1H), 7.87 (d, ^3J = 7.6 Hz, 2H), 7.75 (d, ^3J = 8.2 Hz, 1H), 7.66 (d, ^3J = 7.6 Hz, 1H), 7.63 (d, ^3J = 7.6 Hz, 1H), 7.60 (d, ^3J = 7.8 Hz, 1H), 7.43 – 7.37 (m, 2H), 7.33 – 7.25 (m, 4H), 6.95 (t, ^3J = 7.8 Hz, 1H), 4.24 (ddd, ^3J = 9.9, 8.2, 4.6 Hz, 1H), 4.21 – 4.15 (m, 3H), 3.21 (dd, ^2J = 14.6 Hz, ^3J = 4.6 Hz, 1H), 3.04 (dd, ^2J = 14.6 Hz, ^3J = 9.9 Hz, 1H). **^{13}C-NMR** (126 MHz, DMSO-d_6): δ [ppm] = 173.6, 156.0, 143.8, 143.7, 140.7, 134.4, 128.9, 127.6, 127.1, 125.4, 125.3, 125.1, 123.5, 120.1, 119.9, 117.8, 111.8, 104.2, 65.7, 54.8, 46.6, 26.9. **MS** (ESI, negative ions): found m/z = 502.9 [M(^{79}Br)-H]$^-$, 504.8 [M(^{81}Br)-H]$^-$; calcd. m/z = 503.1 [M(^{79}Br)-H]$^-$, 505.1 [M(^{81}Br)-H]$^-$. The spectroscopic data are in accordance with literature.[2]

Synthesis of $N^α$-Fmoc-4-pinacolatoborono-L-phenylalanine

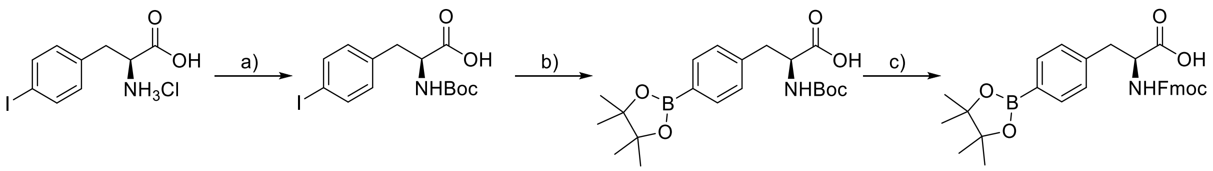

Scheme S1: Reaction conditions: a) Boc_2O, NaOH, H_2O/*tert*-butanol, rt, 19 h, 93%; b) $Pd(OAc)_2$, B_2pin_2, KOAc, DMF, 80 °C, 24 h; c) 1) TFA/DCM, room temperature, 1.5 h, 2) Fmoc-Osu, Na_2CO_3, dioxane/H_2O, room temperature, 17 h, 59% starting from $N^α$-Boc-4-iodo-L-phenylalanine.

$N^α$-Boc-4-iodo-L-phenylalanine: A solution of di-*tert*-butyl dicarbonate (4.310 g, 19.7 mmol, 1.3 equiv) in *tert*-butanol (13 mL) was added dropwise to a solution of 4-iodo-L-phenylalanine·HCl (4.980 g, 17.3 mmol, 1.0 equiv) in aqueous, 1 M NaOH (19 mL, 19 mmol, 1.3 equiv) at 0 °C. The resulting solution was stirred at room temperature for 19 h and afterwards washed with pentane (2 × 20 mL) and aqueous, saturated $NaHCO_3$ solution. The combined, aqueous layers were acidified with aqueous, 5% $KHSO_4$ solution to pH 3, extracted

with ethyl acetate (4 × 50 mL) and the combined, organic layers washed with water and concentrated *in vacuo*. Freeze-drying afforded N^{α}-Boc-4-iodo-L-phenylalanine (5.526 g, 93%) as colourless solid. **^{1}H-NMR** (500 MHz, DMSO-d_6): δ [ppm] = 12.49 (s, 1H), 7.63 (d, ^3J = 8.0 Hz, 2H), 7.12 (d, ^3J = 8.4 Hz, 1H), 7.06 (d, ^3J = 8.0 Hz, 1H), 4.06 (ddd, ^3J = 10.5, 8.5, 4.6 Hz, 1H), 2.96 (dd, ^2J = 13.8 Hz, ^3J = 4.6 Hz, 1H), 2.76 (dd, ^2J = 13.8 Hz, ^3J = 10.5 Hz, 1H), 1.31 (s, 9H). **^{13}C-NMR** (126 MHz, DMSO-d_6): δ [ppm] = 173.4, 155.4, 137.9, 136.8, 131.6, 92.2, 78.1, 54.9, 35.9, 28.1. **MS** (ESI, negative ions): found *m*/*z* = 389.9 $[M-H]^-$; calcd. *m*/*z* = 390.0 $[M-H]^-$. The spectroscopic data are in accordance with literature.[3]

<u>N^{α}-Fmoc-4-pinacolatoborono-L-phenylalanine (Fmoc-Phe(4-Bpin)-OH):</u> Under Argon, a solution of N^{α}-Boc-4-iodo-L-phenylalanine (1.989 g, 5.1 mmol, 1.0 equiv), palladium acetate (120.7 mg, 0.53 mmol, 0.1 equiv), bis(pinacolato)diboron (1.495 g, 5.9 mmol, 1.2 equiv), and potassium acetate (2.562 g, 26.1 mmol, 5.1 equiv) in anhydrous, degassed DMF were heated to 80 °C for 24 h. After cooling to room temperature, the dark brown solution was diluted with ethyl acetate and washed with 1 M hydrochloric acid (2 ×) and brine (2 ×). The combined aqueous layers were extracted with ethyl acetate (3 × 75 mL), dried over sodium sulphate and concentrated *in vacuo*. The resulting dark brown oil was filtrated over silica gel using ethyl acetate as eluent. After concentration, the brown solid was dissolved in a mixture of dichloromethane (7.5 mL) and trifluoroacetic acid (5.5 mL) and stirred at room temperature for 1.5 h. The solution was concentrated and dried over potassium hydroxide in a desiccator at 0.1 mbar overnight. The resulting brown solid was dissolved in a mixture of 1,4-dioxane and water 7:1 (40 mL) and sodium carbonate (2.220 g, 20.9 mmol, 4.1 equiv) and Fmoc-Osu (2.314 g, 6.9 mmol, 1.3 equiv) were added. The solution was stirred at room temperature for 17 h and for workup, dioxane was removed *in vacuo*. The residue was spiked with some water and the pH adjusted to 3 with aqueous, 5% $KHSO_4$ solution, followed by extraction with ethyl acetate (4 × 50 mL). The combined organic layers were washed with brine, dried over sodium sulphate and concentrated *in vacuo*. Purification by column chromatography (SiO_2, DCM/MeOH, gradient from 99:1 to 95:5) afforded N^{α}-Fmoc-4-pinacolatoborono-L-phenylalanine (1.573 g, 59%) as slightly orange solid. **^{1}H NMR** (500 MHz, $CDCl_3$): δ[ppm] = 7.82 – 7.71 (m, 4H), 7.55 (t, *J* = 9.0 Hz, 2H), 7.44 – 7.36 (m, 2H), 7.35 – 7.27 (m, 2H), 7.20 (d, *J* = 7.5 Hz, 2H), 5.30 (d, *J* = 8.2 Hz, 1H), 4.77 – 4.68 (m, 1H), 4.48 – 4.29 (m, 2H), 4.21 (t, *J* = 7.3 Hz, 1H), 3.25 (dd, *J* = 13.8, 5.4 Hz, 1H), 3.15 (dd, *J* = 13.7, 6.2 Hz, 1H), 1.35 (s, 12H). **^{13}C NMR** (126 MHz, $CDCl_3$): δ[ppm] = 175.5, 156.0, 143.9, 143.8, 141.4, 139.0, 135.3, 128.9, 127.8, 127.2, 125.3, 125.2, 120.1, 84.1, 67.3, 54.6, 47.2, 38.0, 25.0. **MS** (ESI, positive ions): found *m*/*z* = 536.2 $[M+Na]^+$; calcd. *m*/*z* = 536.4 $[M+Na]^+$. The spectroscopic data are in accordance with literature.[4]

Peptide synthesis

Peptide synthesis was performed on Rink Amide AM resin using Fmoc/*t*-Bu strategy.

<u>Fmoc-deprotection:</u> The resin was swollen in DMF for 10 min and filtrated. A solution of piperidine (20% v/v) and 0.1 M HOBt in DMF was added to the resin and incubated for 20 min at room temperature. The suspension was filtrated and the procedure repeated once. The resin was washed with DMF (3 × 1 min) and DCM (3 × 1 min).

<u>Amino acid coupling:</u> The resin was swollen in DMF for 10 min and filtrated. *Coupling of protected proteinogenic amino acids:* In a vial, Fmoc-AA-OH (5 equiv) and TBTU (5 equiv) were dissolved in DMF and DIEA (10 equiv) was added. The resulting solution was gently shaken for 30 s, added to the resin and the suspension incubated at room temperature for 60 min. The suspension was filtrated and the resin washed with DMF (3 × 1 min) and DCM (3 × 1 min). *Coupling of Fmoc-Trp(Br)-OH and Fmoc-Phe(4-Bpin)-OH:* In a vial, Fmoc-AA-OH (1.3 equiv) and HATU (1.3 equiv) were dissolved in DMF and DIEA (2.6 equiv) was added. The resulting solution was gently shaken for 30 s, added to the resin and the suspension incubated at room temperature for 60 min. The suspension was filtrated and the resin washed with DMF (3 × 1 min) and DCM (3 × 1 min). *Coupling of 4-carboxyphenylboronic acid and 4-(2-carboxyethyl)phenylboronic acid:* In a vial, the carboxylic acid (3 equiv) and HATU (3 equiv) were dissolved in DMF and DIEA (6 equiv) was added. The resulting solution was gently shaken for 30 s, added to the resin and the suspension incubated at room temperature for 60 min. The suspension was filtrated and the resin washed with DMF (3 × 1 min) and DCM (3 × 1 min). *Coupling of Fmoc-S_5-OH:* In a vial, Fmoc-S_5-OH (3 equiv) and HATU (3 equiv) were dissolved in NMP and DIEA (6 equiv) was added. The resulting solution was gently shaken for 30 s, added to the resin and the suspension incubated at room temperature for 60 min. The suspension was filtrated and the resin washed with NMP (3 × 1 min), DMF (3 × 1 min) and DCM (3 × 1 min).

<u>Resin loading:</u> The resin was swollen in DMF for 10 min and filtrated. Fmoc-deprotection was performed following the procedure listed above. Amino acid coupling was performed following the procedure listed above but using 0.2 mmol Fmoc-Met-OH, 0.2 mmol TBTU and 0.4 mmol DIEA in DMF per gram of resin. Resin loading was determined using UV spectroscopy. For this purpose, the resin was washed with DMF (3 × 1 min), DCM (3 × 1 min), iPrOH (3 × 1 min) and MTBE (3 × 1 min) and dried *in vacuo*. Next, 1 mg of the resin was weighed and suspended in 3 mL of a solution of piperidine (20% v/v) in DMF and incubated for 20 min. The absorption of the resulting solution was determined at 290 nm and the loading determined with the following equation:[5]

$$\text{Loading [mmol g}^{-1}\text{]} = \frac{A_{290}}{m \cdot 1.65}$$

with A_{290} = absorption at 290 nm und m = weight of resin sample [mg].

Alloc-deprotection: The resin was swollen in anhydrous DCM for 10 min and filtrated. A solution of tetrakis(triphenylphosphine)palladium(0) (0.1 equiv) and morpholine (10 equiv) in anhydrous DCM was added to the resin and mixed by a gentle stream of argon for 20 min at room temperature. The suspension was filtrated and the procedure repeated once. The resin was washed with DCM (3 × 1 min), DMF (3 × 1 min) and DCM (3 × 1 min).

Kaiser test: To ensure complete couplings, a Kaiser test was performed after each coupling step. After the resin was washed with DMF (3 × 1 min), DCM (3 × 1 min), iPrOH (3 × 1 min) and MTBE (3 × 1 min), it was dried *in vacuo*. A few resin beads were transferred into an Eppendorf tube, three drops of each solution A, B and C were added and incubated for 5 min at 100 °C. Incomplete couplings were indicated by either blue beads or a blue solution. Solution A: 500 mg ninhydrin in 10 mL EtOH (abs.), solution B: 100 mg phenol in 25 mL EtOH (abs.), solution C: 1 mM NaCN in 49 mL pyridine.[6]

Test cleavage: The resin was washed with DMF (3 × 1 min), DCM (3 × 1 min), iPrOH (3 × 1 min) and MTBE (3 × 1 min) and dried *in vacuo*. A few resin beads were transferred into an Eppendorf tube, 100 µL of a solution of TFA/TIS/H_2O 95:2.5:2.5 including DTT (2.5% w/v) and phenol (2.5% w/v) were added and the resulting suspension incubated at room temperature for 60 min. Next, 1.4 mL of cold MTBE were added, the sample spun down, the supernatant discarded, the residue dried by a stream of Argon and dissolved in a mixture of ACN/H_2O 1:1. The sample was spun down again and the supernatant either analysed by HPLC-MS or MALDI-ToF.

Capping: After Fmoc-deprotection, the resin was swollen in DMF for 10 min and filtrated. A solution of acetic anhydride (10 equiv) and pyridine (10 equiv) in DMF was added and incubated for 20 min at room temperature. The suspension was filtrated and the procedure repeated once. The resin was washed with DMF (3 × 1 min) and DCM (3 × 1 min).

FITC-labelling: After *N*-terminal Fmoc-deprotection, the resin was swollen in DMF for 10 min and filtrated. Fmoc-β-alanine coupling was performed following the procedure listed above but using Fmoc-β-Ala-OH (5 equiv), HATU (5 equiv) and DIEA (10 equiv) in DMF. The suspension was filtrated and the procedure repeated once. The resin was washed with DMF (3 × 1 min), DCM (3 × 1 min) and DMF (3 × 1 min) followed by Fmoc-deprotection. Next, the resin was swollen in DMF for 10 min, filtrated and a solution of FITC (7 equiv) and DIEA (7 equiv) in DMF was added and the suspension shaken at room temperature for 18 h under light protection. The suspension was filtrated, the resin washed with DMF (3 × 1 min), DCM (3 × 1 min), iPrOH (3 × 1 min) and MTBE (3 × 1 min) and dried *in vacuo*. FITC-labelled peptides should be stored under light protection.[7]

Cleavage and purification: A degassed solution of TFA/TIS/H_2O 95:2.5:2.5 including DTT (2.5% w/v) and phenol (2.5% w/v) was added to the resin and the resulting suspension incubated at room temperature for 120 min. From time to time, the suspension was mixed by

a gentle stream of argon. The suspension was filtrated and the procedure was repeated once. The TFA solution obtained was added dropwise into cold MTBE and the resulting turbid suspension incubated at −20 °C for 60 min. Next, the precipitated peptide was spun down at 4 °C at 5000 rpm for 30 min, the supernatant was discarded and the residue dried by a stream of argon. The crude peptide was freeze dried and purified by preparative RP-HPLC (method A).

GP3: General procedure for on-resin Suzuki–Miyaura cross-coupling

Under Argon, a degassed mixture of DME/EtOH/H_2O 9:9:2 (1 mL per 50 µmol peptide) was added to the resin and KF (4 equiv) in a pressure-sealed microwave vial. Subsequently, solutions of sSPhos (0.4 equiv) and $Pd_2(dba)_3$ (0.2 equiv) in degassed mixtures of DME/EtOH/H_2O 9:9:2 (1 mL per 50 µmol peptide each) were added causing a total reaction volume of 3 mL. The suspension was heated under microwave irradiation (50 W) and stirring at 120 °C for 30 min. The resulting suspension was filtrated, the black resin washed with DMF (3 × 1 min), EtOH (3 × 1 min), DCM (3 × 1 min) and MTBE (3 × 1 min) and dried *in vacuo*. The peptide was cleaved from the resin and purified by RP-HPLC (protocols see section "Peptide Synthesis").[8]

GP4: General procedure for on-resin ring-closing metathesis

The resin was swollen in DCM (3 × 1 min) and DCE (3 × 1 min) and filtrated. Next, an 8.3 mM solution of Grubbs' 1st generation catalyst (0.5 equiv) in DCE was added and the suspension mixed by a gentle stream of argon for 120 min. The suspension was filtrated and the procedure repeated once. The resin was washed with DCE (3 × 1 min), DCM (3 × 1 min) and MTBE and dried *in vacuo*. A test cleavage was performed (see section "Peptide Synthesis") and analysed by HPLC-MS. If the conversion was incomplete, metathesis has been repeated as described above until a conversion >95% was observed. The peptide was cleaved from the resin and purified by RP-HPLC (protocols see section "Peptide Synthesis").[7]

Example MALDI TOF MS of crude peptide P1b

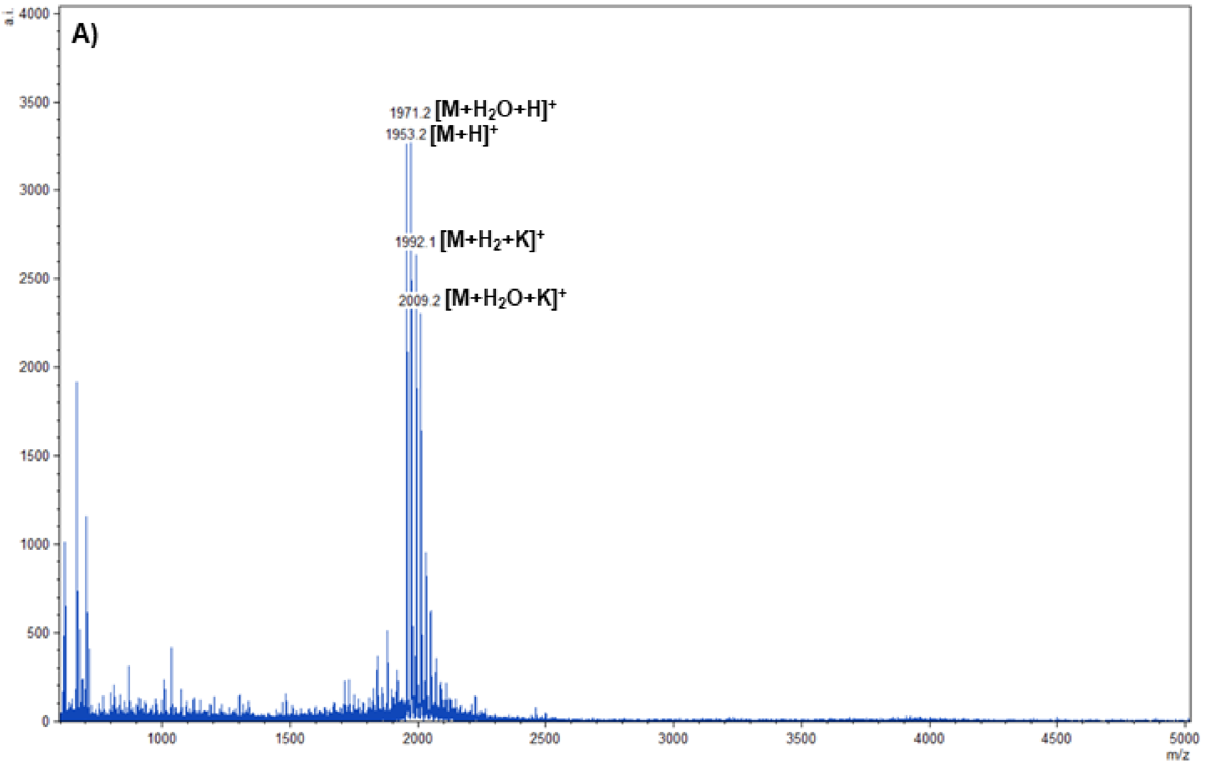

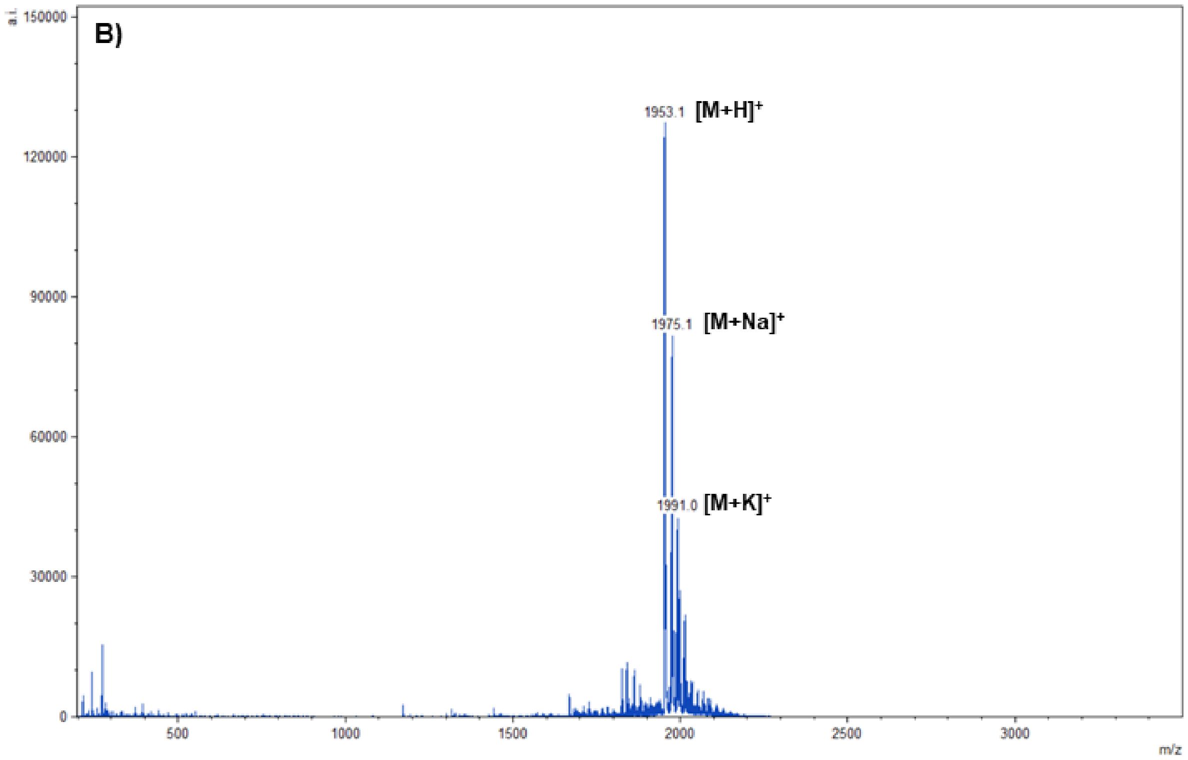

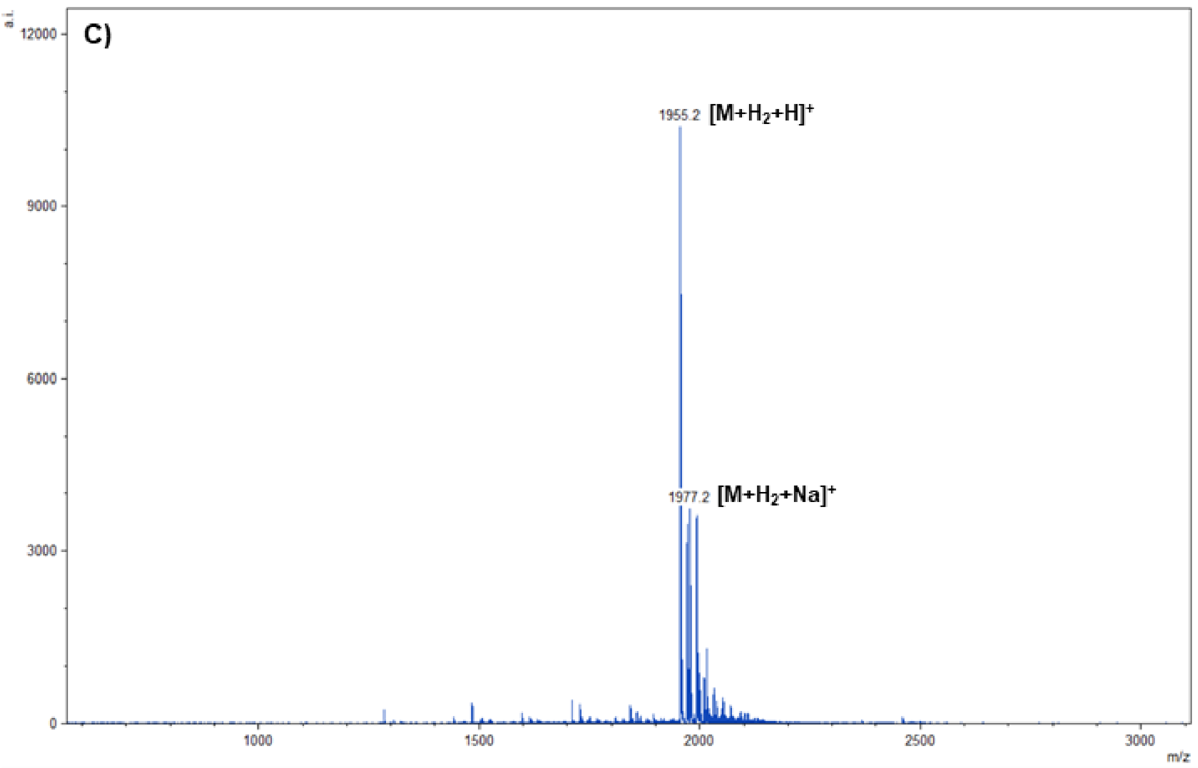

Figure S1: MALDI TOF MS analysis of peptide **P1b** after on-resin SMC. **A**) Spectrum of the crude peptide after test cleavage indicating that no intermolecular dimerisation products were formed; **B**) Spectrum of the product fraction after purification by preparative RP-HPLC; **C**) Spectrum of the fraction containing the deboronated and dehalogenated species ($M+H_2$).

Analytical data of peptides

SMC-stapled peptides

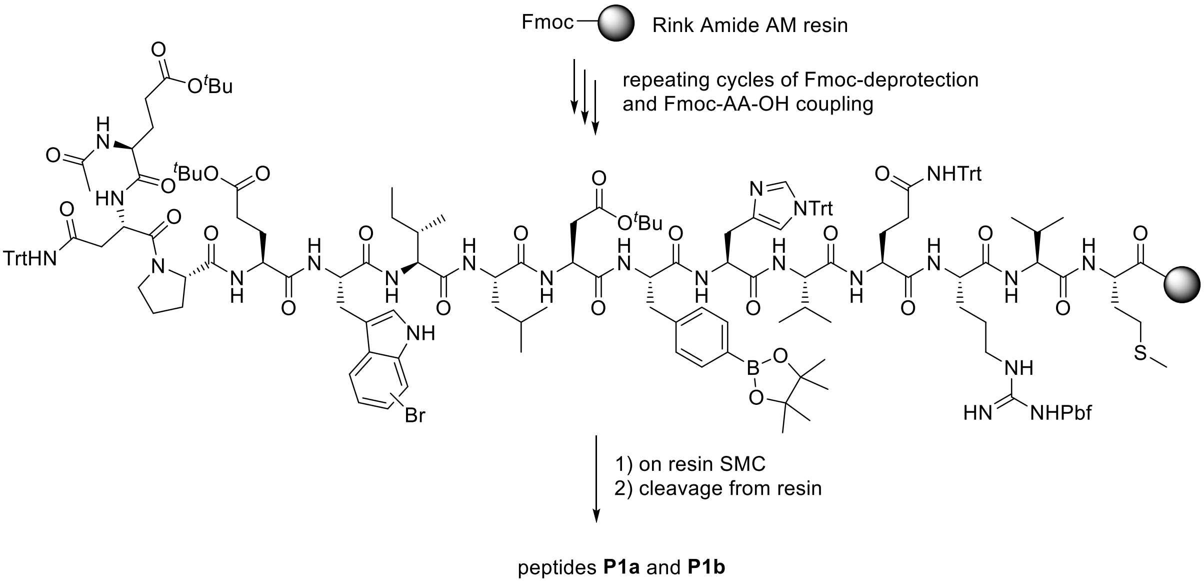

Scheme S2: General overview of the on-resin synthesis of peptides **P1a** and **P1b**.

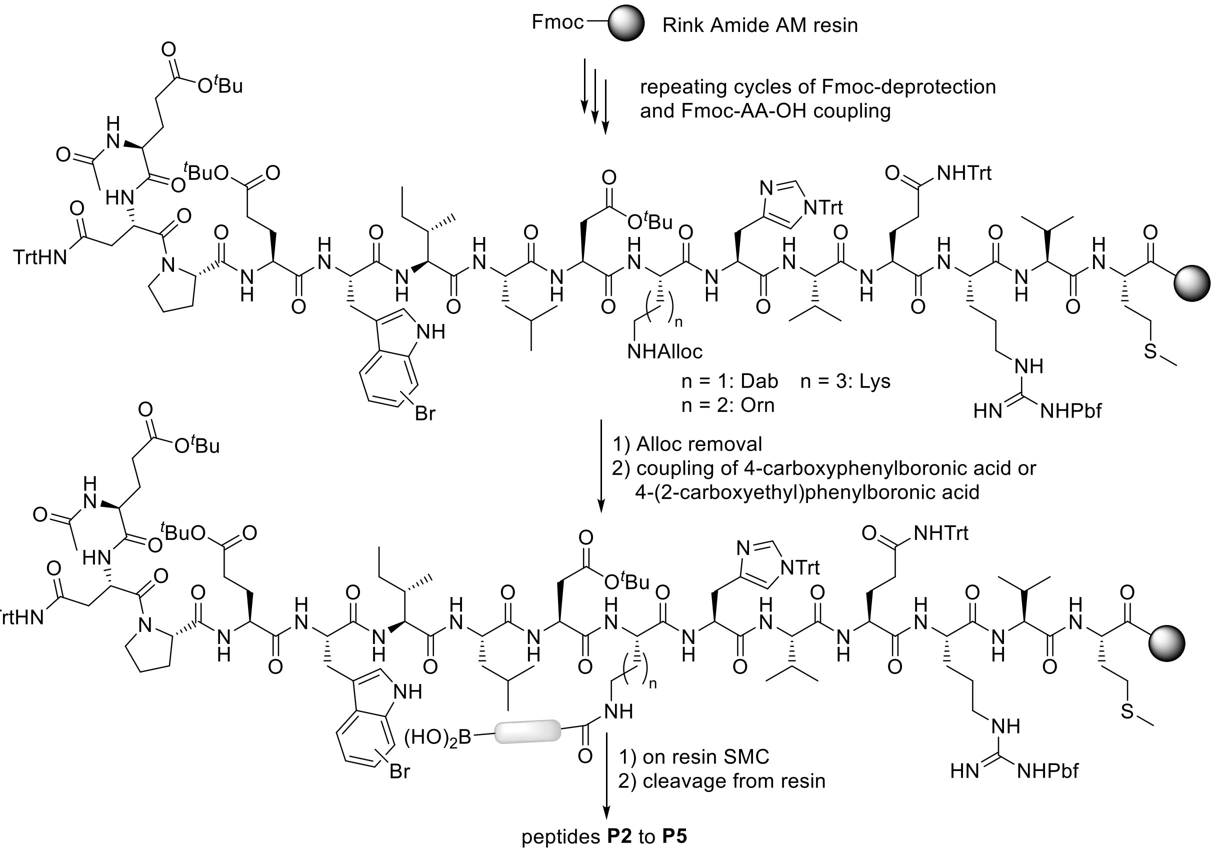

Scheme S3: General overview of the on-resin synthesis of peptides **P2** to **P5**.

Peptide **P1a**

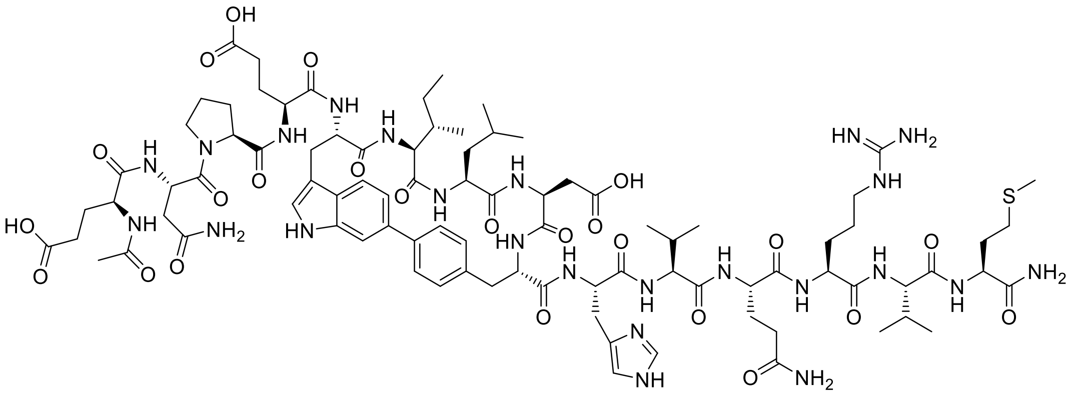

$C_{89}H_{130}N_{24}O_{24}S$
M = 1952.22 g mol^{-1}
M x TFA = 2066.24 g mol^{-1}

The synthesis was performed following the general procedures on a 50 µmol scale. Peptide **P1a** (3.8 mg, 4% overall isolated yield referring to initial resin loading) was obtained as colourless solid after purification by preparative RP-HPLC (method A). **RP-HPLC:** t_R = 4.5 min. **HRMS** (ESI, positive ions): found m/z = 976.4749 $[M+2H]^{2+}$; calcd. m/z = 976.4778 $[M+2H]^{2+}$; deviation = 2.97 ppm.

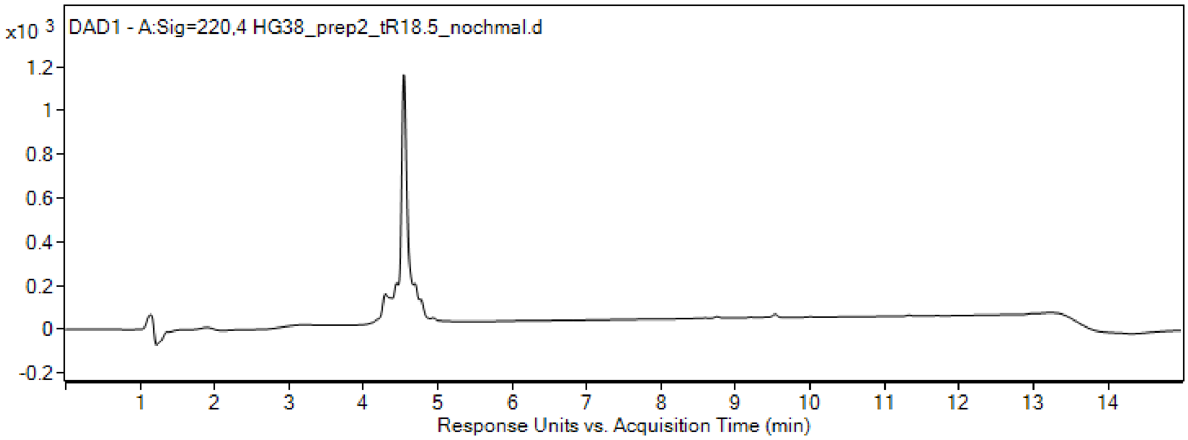

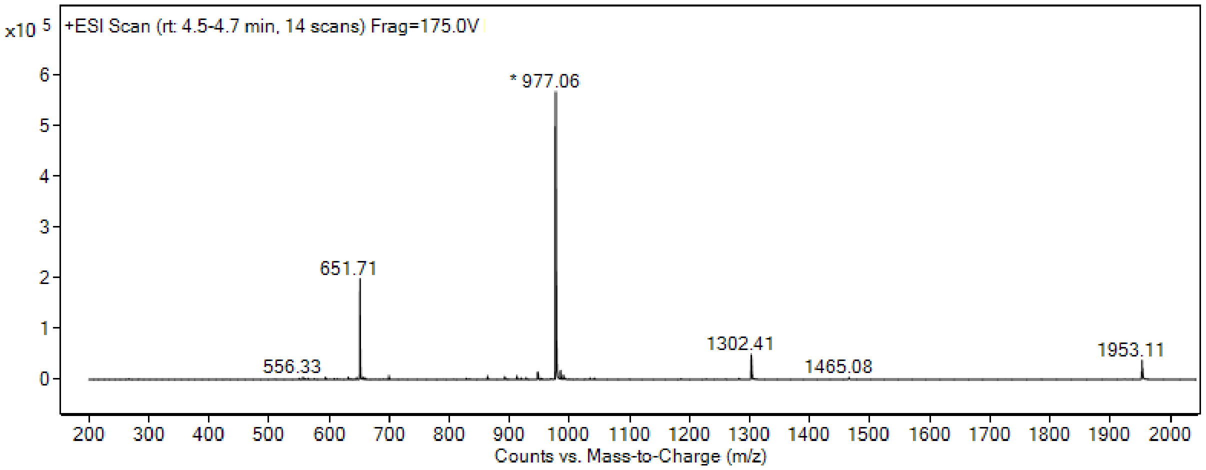

Peptide **P1b**

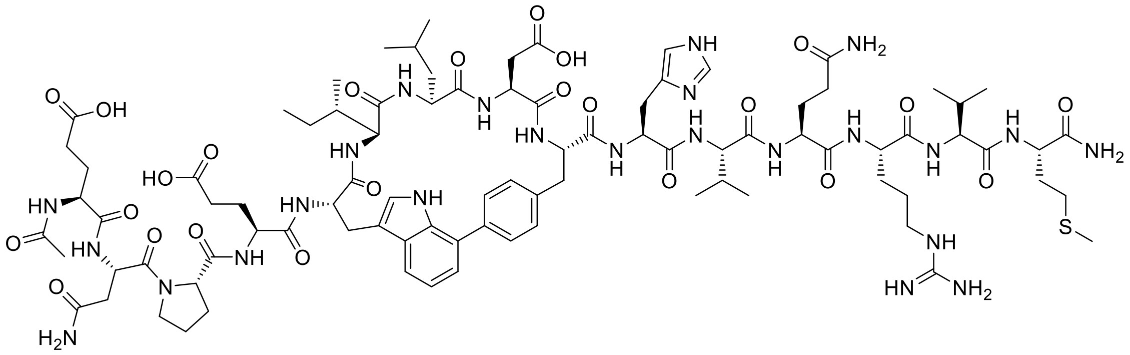

$C_{89}H_{130}N_{24}O_{24}S$
M = 1952.22 g mol^{-1}
M x TFA = 2066.24 g mol^{-1}

The synthesis was performed following the general procedures on a 50 µmol scale. Peptide **P1b** (3.5 mg, 3% overall isolated yield referring to initial resin loading) was obtained as colourless solid after purification by preparative RP-HPLC (method A). **RP-HPLC:** t_R = 5.1 min. **HRMS** (ESI, positive ions): found *m*/*z* = 976.4757 [M+2H]$^{2+}$; calcd. *m*/*z* = 976.4778 [M+2H]$^{2+}$; deviation = 2.15 ppm.

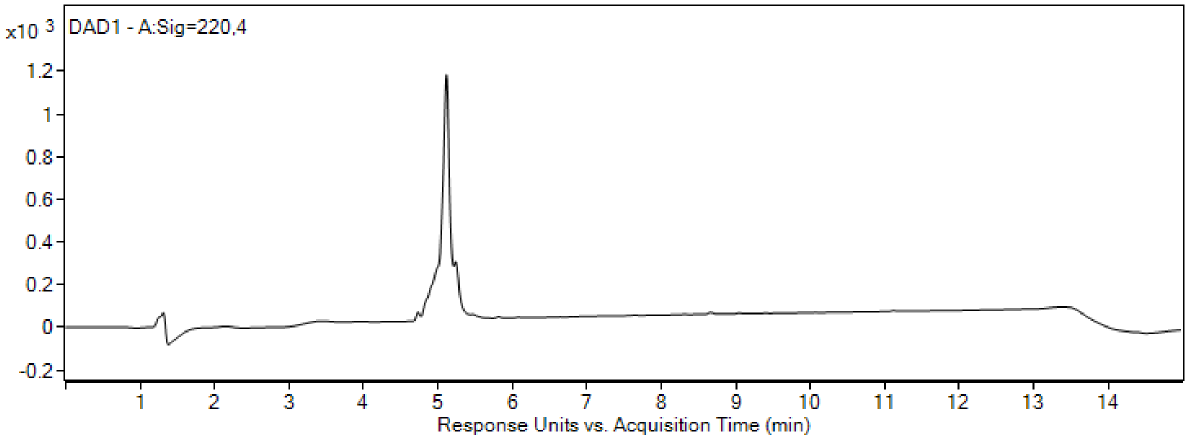
x10 3
DAD1 - A:Sig=220,4
Response Units vs. Acquisition Time (min)

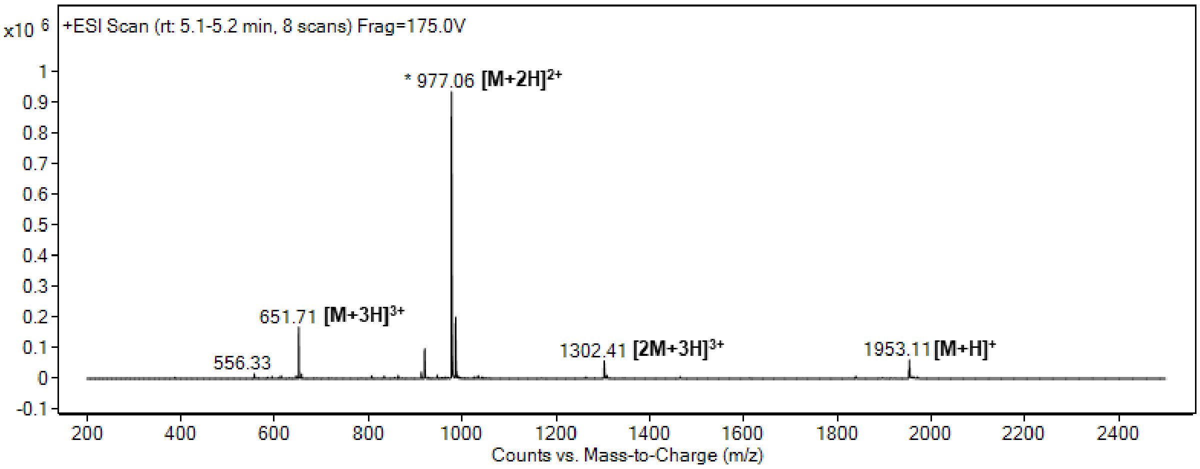
x10 6
+ESI Scan (rt: 5.1-5.2 min, 8 scans) Frag=175.0V
* 977.06 [M+2H]2+
651.71 [M+3H]3+
556.33
1302.41 [2M+3H]3+
1953.11 [M+H]+
Counts vs. Mass-to-Charge (m/z)

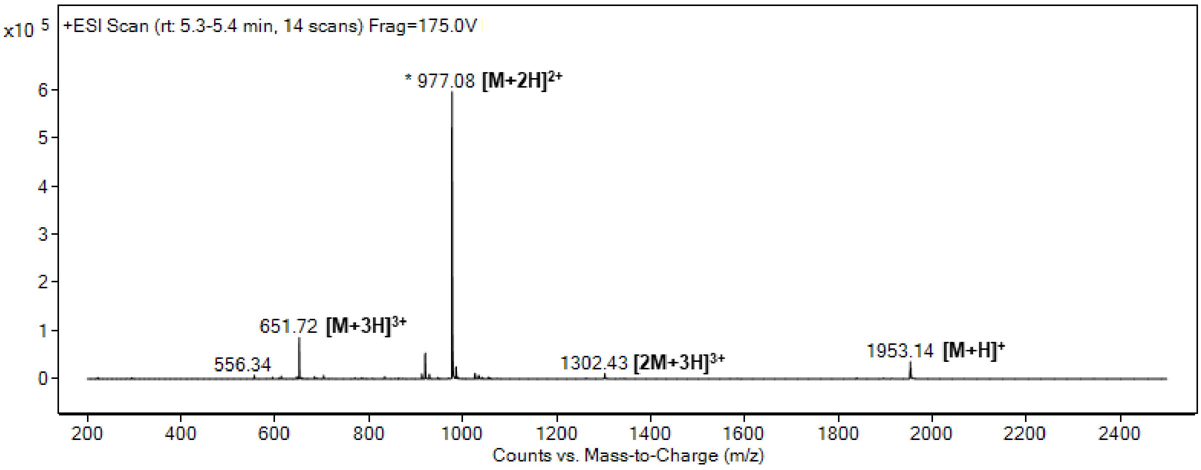
x10 5
+ESI Scan (rt: 5.3-5.4 min, 14 scans) Frag=175.0V
* 977.08 [M+2H]2+
651.72 [M+3H]3+
556.34
1302.43 [2M+3H]3+
1953.14 [M+H]+
Counts vs. Mass-to-Charge (m/z)

Peptide **P2a**

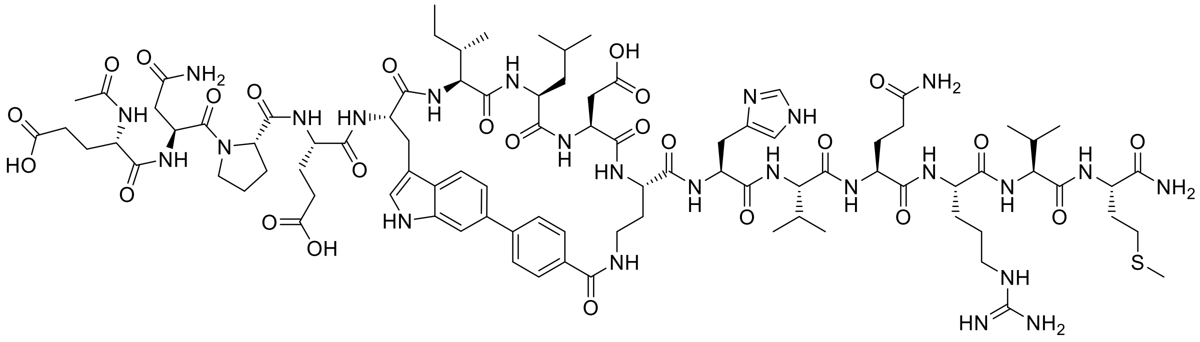

$C_{91}H_{133}N_{25}O_{25}S$
M = 2009.28 g mol^{-1}
M x TFA = 2123.30 g mol^{-1}

The synthesis was performed following the general procedures on a 52 µmol scale. Peptide **P2a** (9.1 mg, 8% overall isolated yield referring to initial resin loading) was obtained as colourless solid after purification by preparative RP-HPLC (method A). **RP-HPLC:** t_R = 4.5 min. **HRMS** (ESI, positive ions): found m/z = 1004.9883 $[M+2H]^{2+}$; calcd. m/z = 1004.9885 $[M+2H]^{2+}$; deviation = 0.23 ppm.

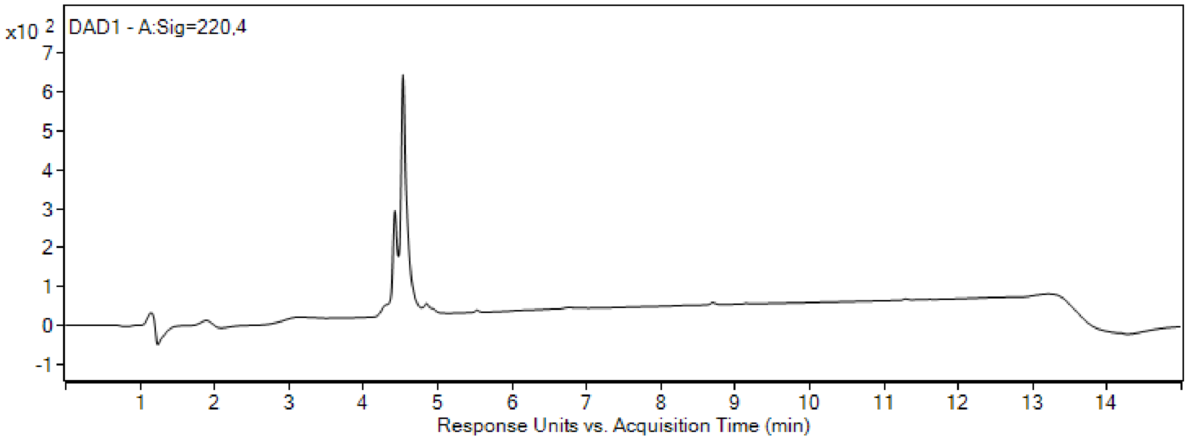

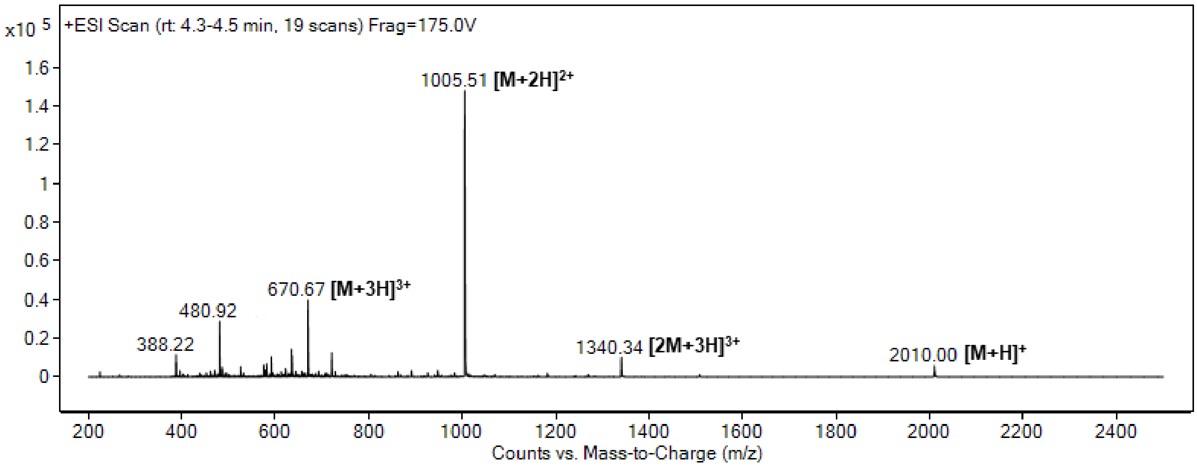

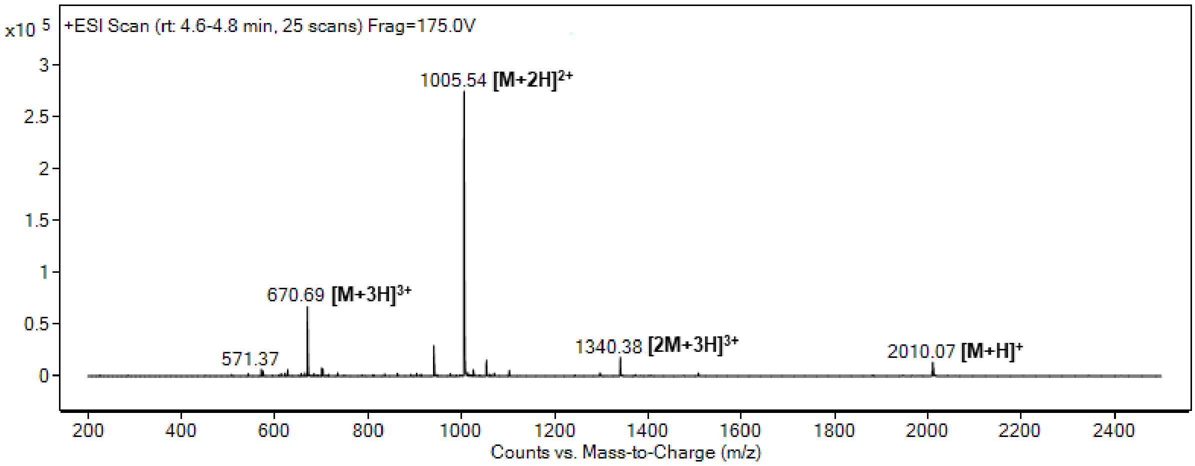

Peptide **P2b**

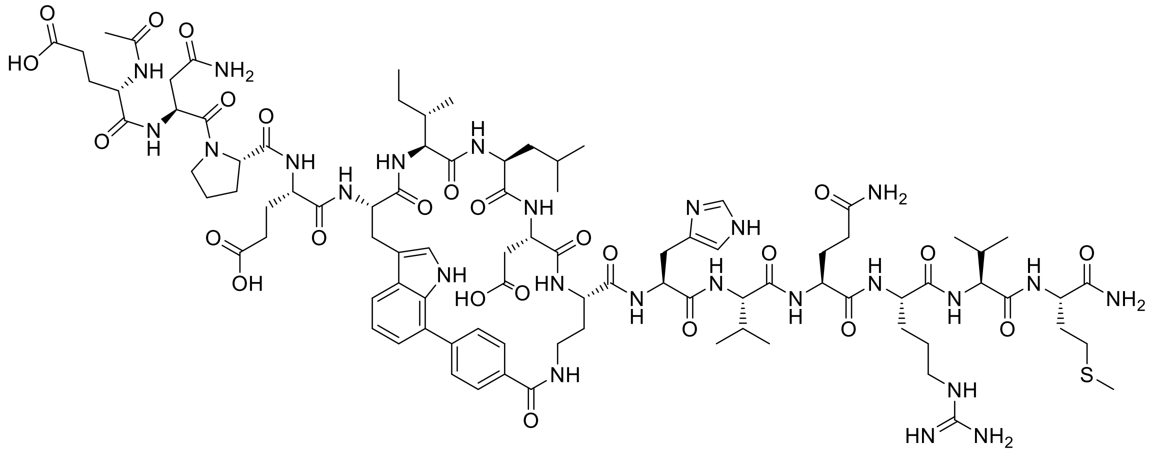

$C_{91}H_{133}N_{25}O_{25}S$
M = 2009.28 g mol^{-1}
M x TFA = 2123.30 g mol^{-1}

The synthesis was performed following the general procedures on a 52 µmol scale. Peptide **P2b** (9.6 mg, 9% overall isolated yield referring to initial resin loading) was obtained as colourless solid after purification by preparative RP-HPLC (method A). **RP-HPLC:** t_R = 4.9 min. **HRMS** (ESI, positive ions): found m/z = 1004.9883 [M+2H]$^{2+}$; calcd. m/z = 1004.9885 [M+2H]$^{2+}$; deviation = 0.2 ppm.

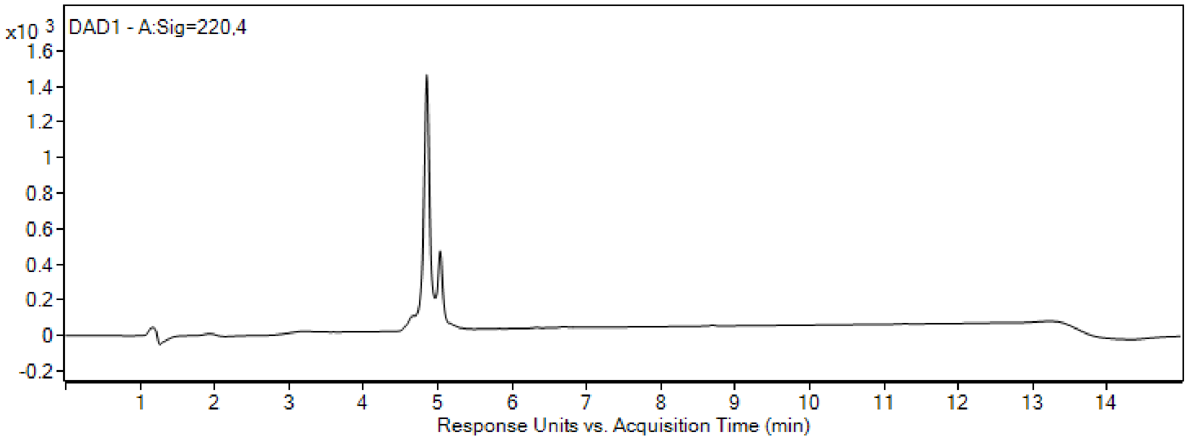

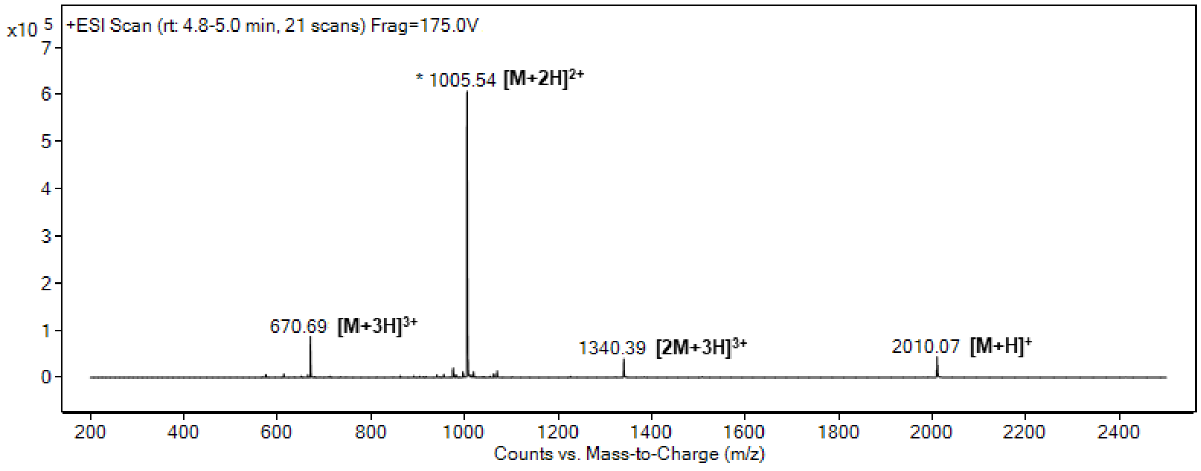

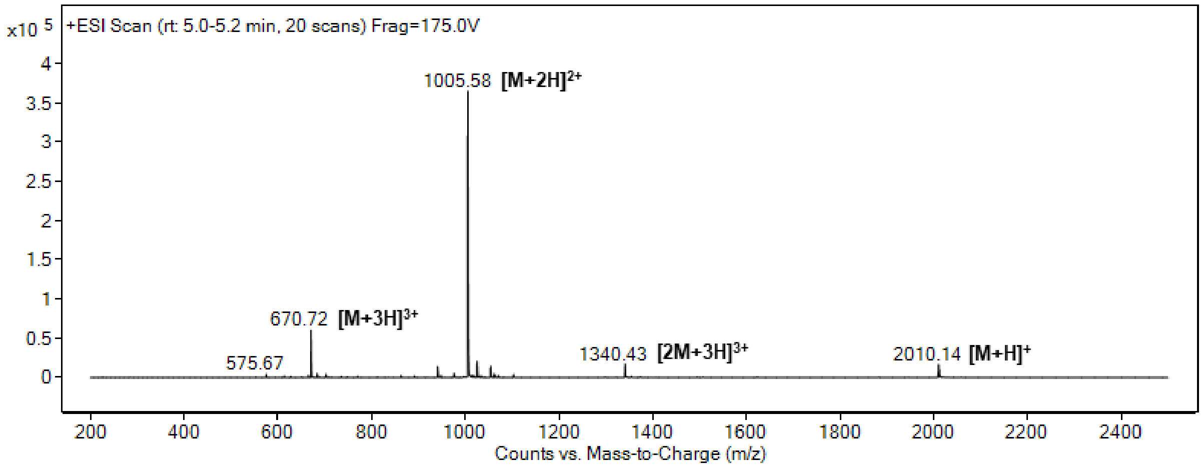

Peptide **P3a**

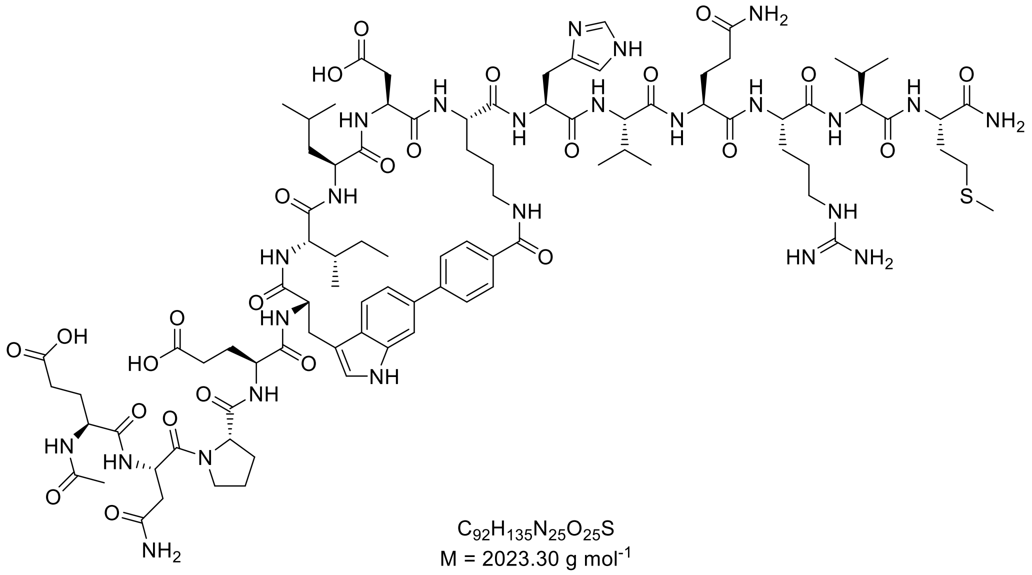

$C_{92}H_{135}N_{25}O_{25}S$
M = 2023.30 g mol^{-1}
M x TFA = 2137.32 g mol^{-1}

The synthesis was performed following the general procedures on a 42 µmol scale. Peptide **P3a** (6.8 mg, 8% overall isolated yield referring to initial resin loading) was obtained as colourless solid after purification by preparative RP-HPLC (method A). **RP-HPLC:** t_R = 4.5 min.

HRMS (ESI, positive ions): found *m*/*z* = 1011.9976 $[M+2H]^{2+}$; calcd. *m*/*z* = 1011.9964 $[M+2H]^{2+}$; deviation = 1.23 ppm.

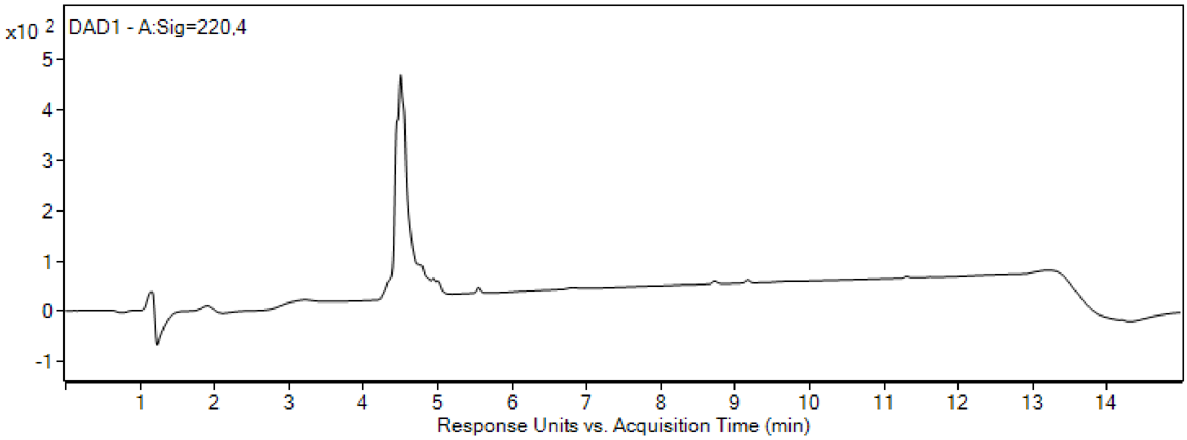

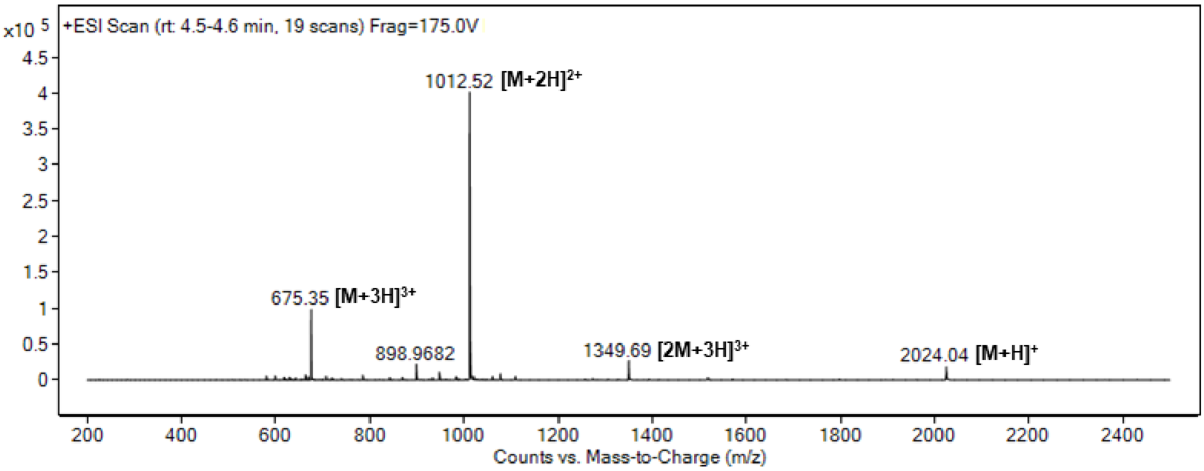

Peptide **P3b**

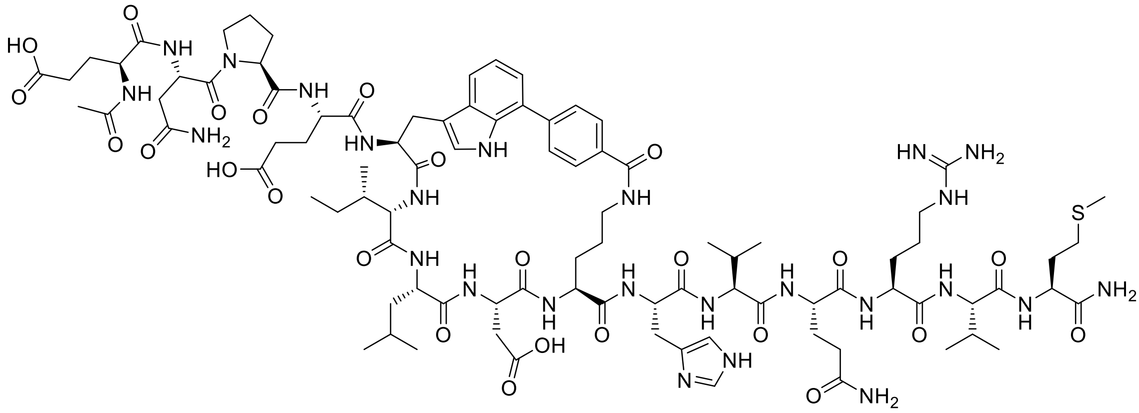

$C_{92}H_{135}N_{25}O_{25}S$
M = 2023.30 g mol^{-1}
M x TFA = 2137.32 g mol^{-1}

The synthesis was performed following the general procedures on a 52 µmol scale. Peptide **P3b** (8.5 mg, 8% overall isolated yield referring to initial resin loading) was obtained as colourless solid after purification by preparative RP-HPLC (method A). **RP-HPLC:** t_R = 4.8 min.

HRMS (ESI, positive ions): found *m*/*z* = 1011.9974 $[M+2H]^{2+}$; calcd. *m*/*z* = 1011.9964 $[M+2H]^{2+}$; deviation = 1.03 ppm.

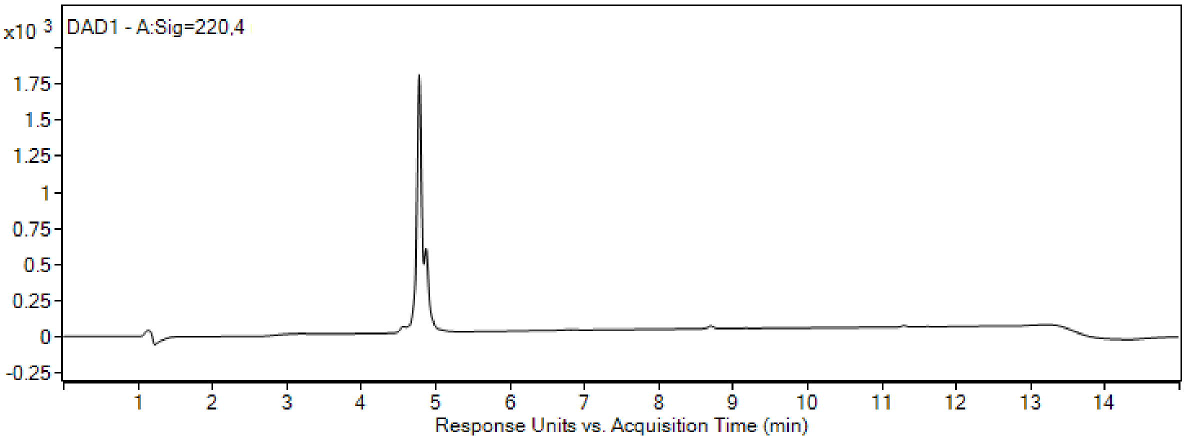

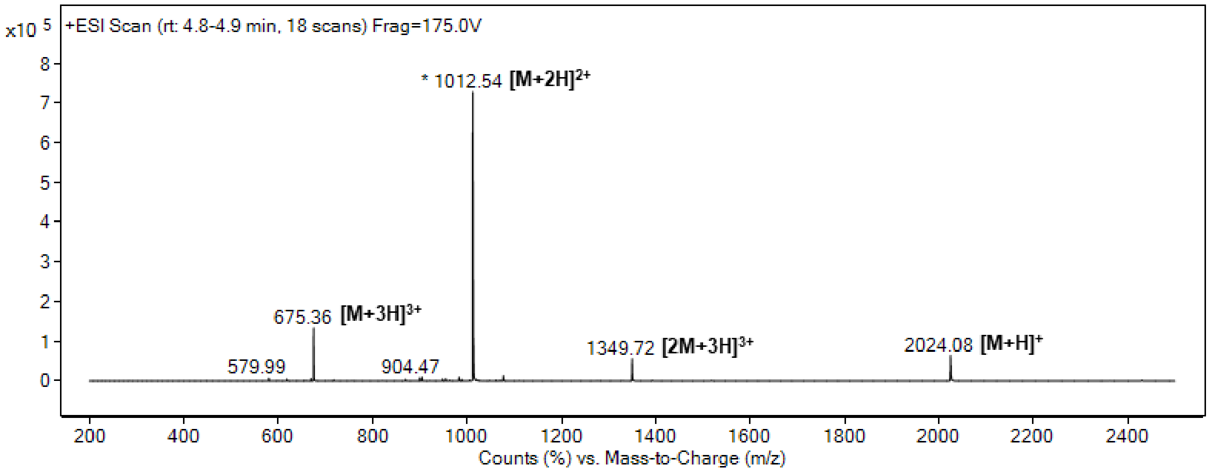

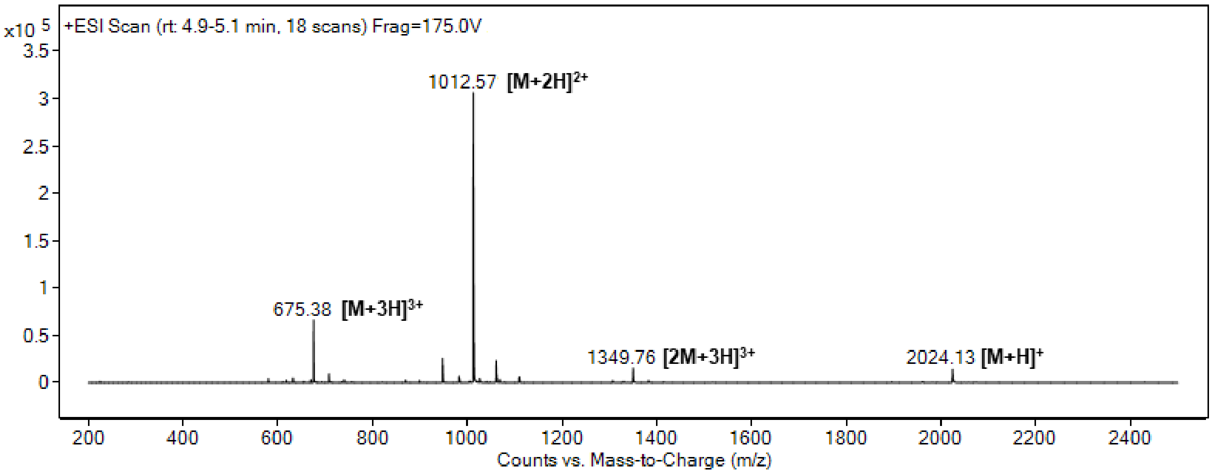

<u>Peptide **P4a**</u>

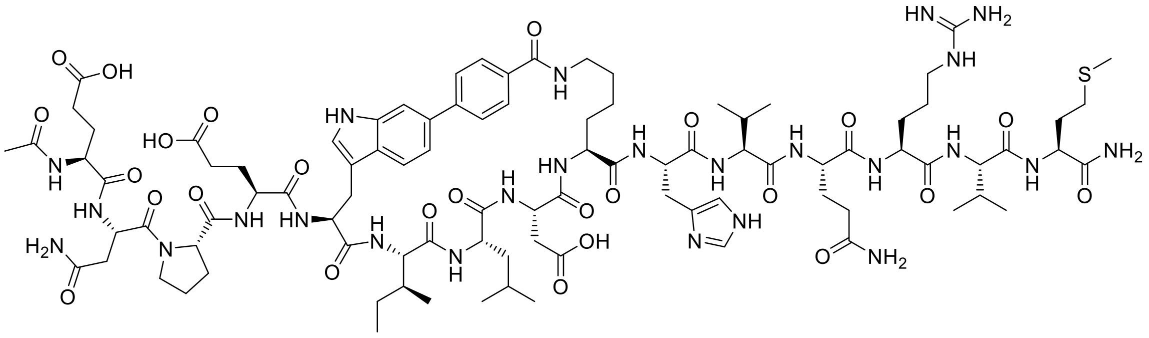

$C_{93}H_{137}N_{25}O_{25}S$
M = 2037.33 g mol^{-1}
M x TFA = 2151.35 g mol^{-1}

The synthesis was performed following the general procedures on a 50 µmol scale. Peptide **P4a** (11.0 mg, 10% overall isolated yield referring to initial resin loading) was obtained as colourless solid after purification by preparative RP-HPLC (method A). **RP-HPLC:** t_R = 4.8 min. **HRMS** (ESI, positive ions): found *m*/*z* = 1019.0026 [M+2H]$^{2+}$; calcd. *m*/*z* = 1019.0042 [M+2H]$^{2+}$; deviation = 1.55 ppm.

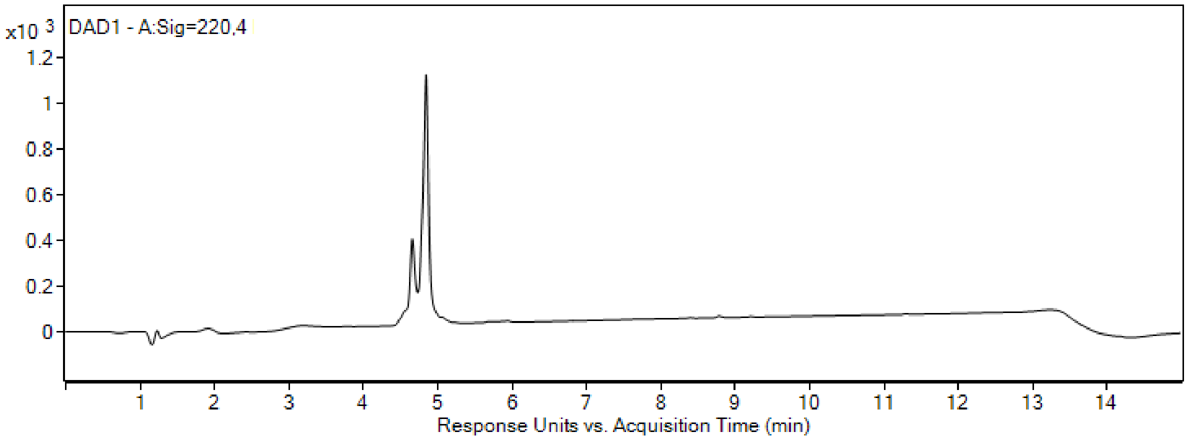

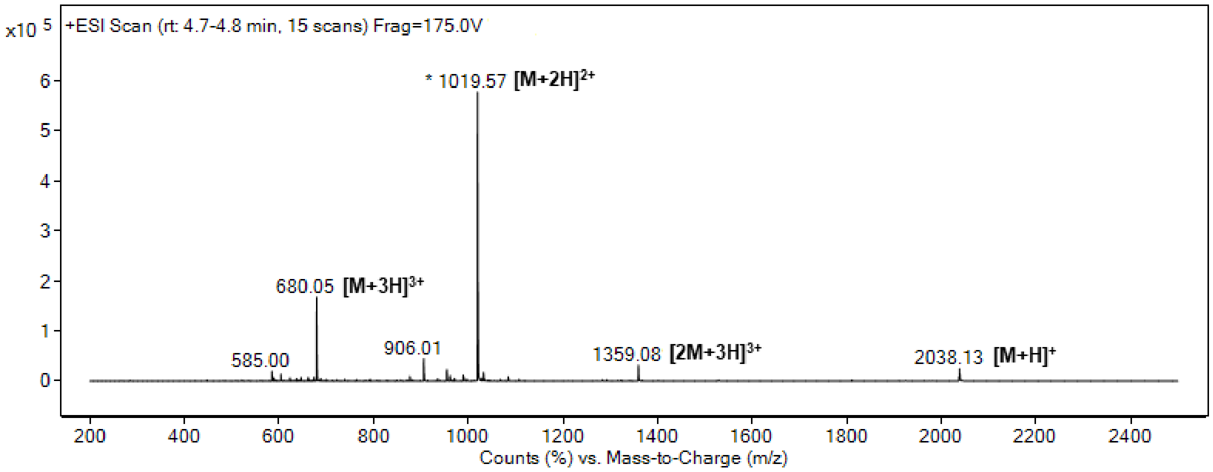

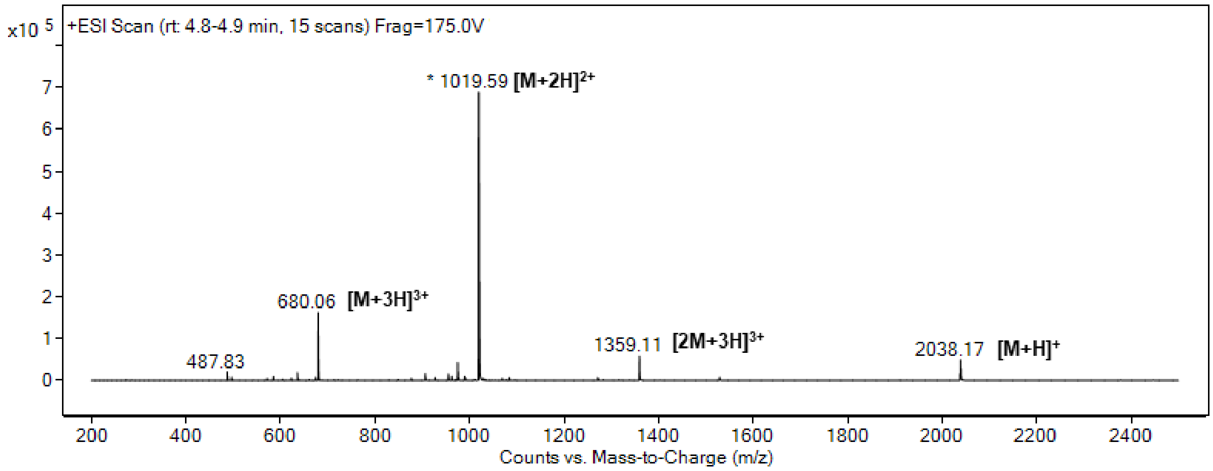

Peptide **P4b**

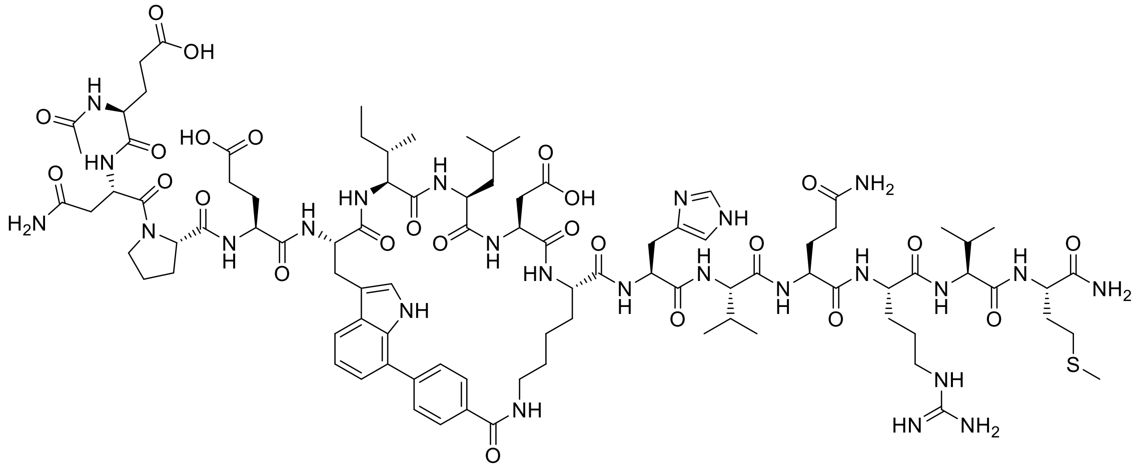

$C_{93}H_{137}N_{25}O_{25}S$
M = 2037.33 g mol^{-1}
M x TFA = 2151.35 g mol^{-1}

The synthesis was performed following the general procedures on a 50 µmol scale. Peptide **P4b** (11.4 mg, 11% overall isolated yield referring to initial resin loading) was obtained as colourless solid after purification by preparative RP-HPLC (method A). **RP-HPLC:** t_R = 5.2 min. **HRMS** (ESI, positive ions): found m/z = 1019.0034 $[M+2H]^{2+}$; calcd. m/z = 1019.0042 $[M+2H]^{2+}$; deviation = 0.77 ppm.

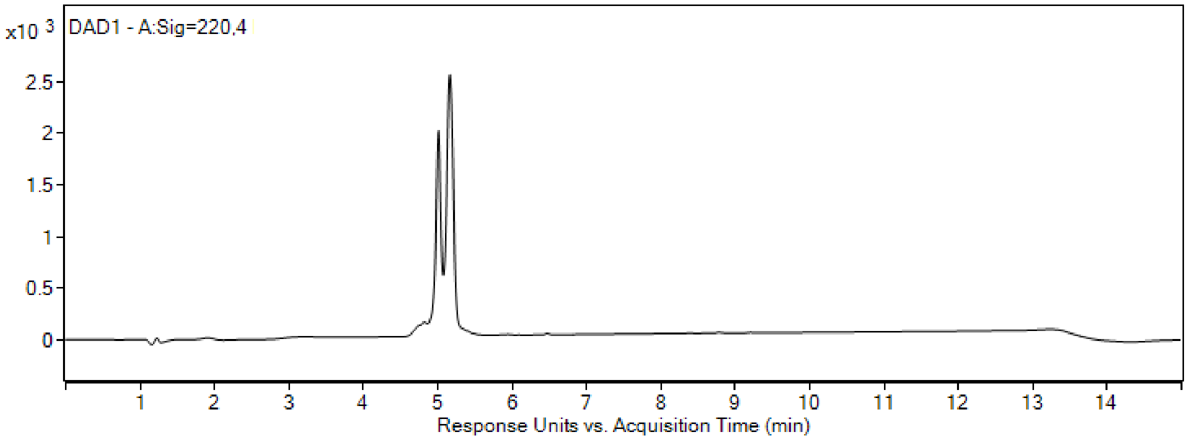
x10 3
DAD1 - A:Sig=220,4
Response Units vs. Acquisition Time (min)

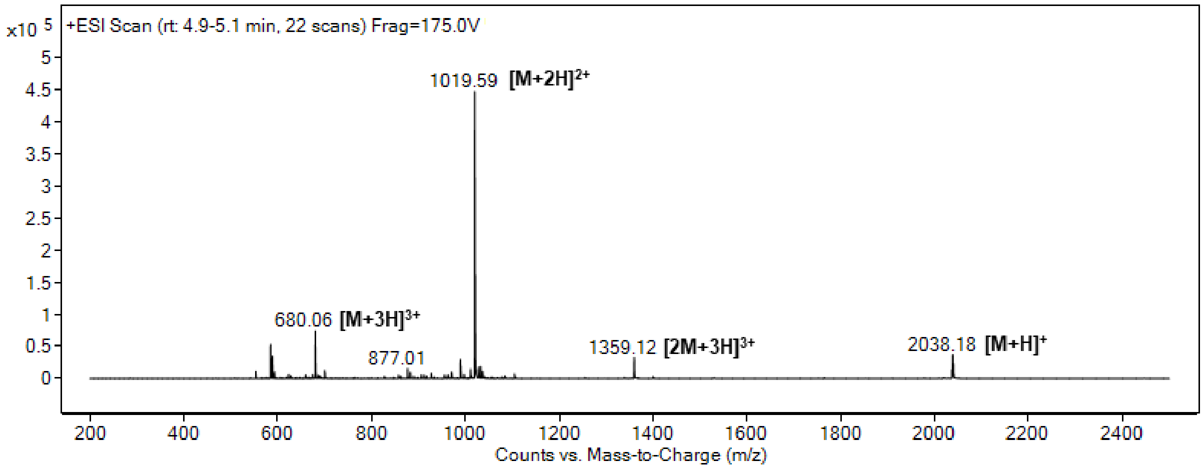
x10 5
+ESI Scan (rt: 4.9-5.1 min, 22 scans) Frag=175.0V
1019.59 [M+2H]2+
680.06 [M+3H]3+
877.01
1359.12 [2M+3H]3+
2038.18 [M+H]+
Counts vs. Mass-to-Charge (m/z)

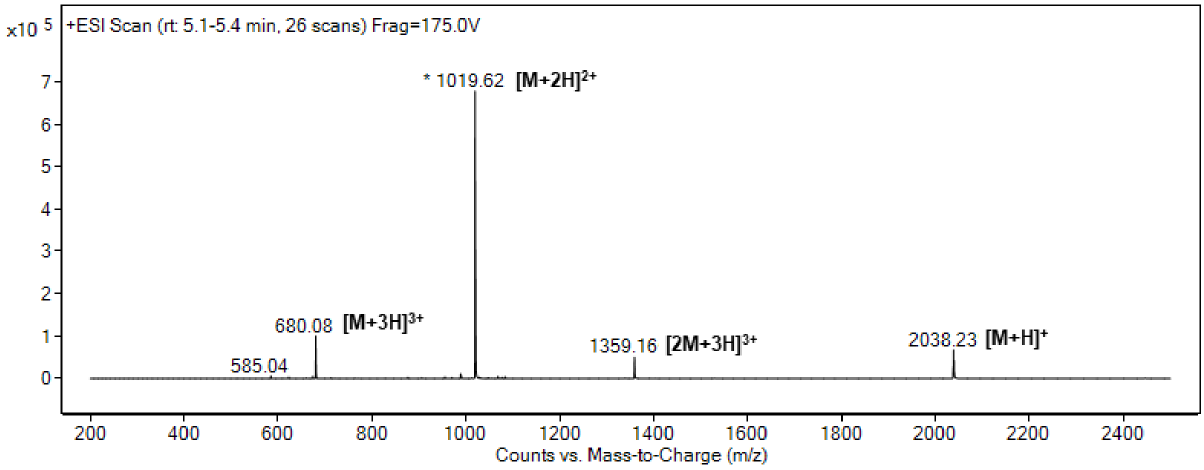
x10 5
+ESI Scan (rt: 5.1-5.4 min, 26 scans) Frag=175.0V
* 1019.62 [M+2H]2+
680.08 [M+3H]3+
585.04
1359.16 [2M+3H]3+
2038.23 [M+H]+
Counts vs. Mass-to-Charge (m/z)

Peptide **P5**

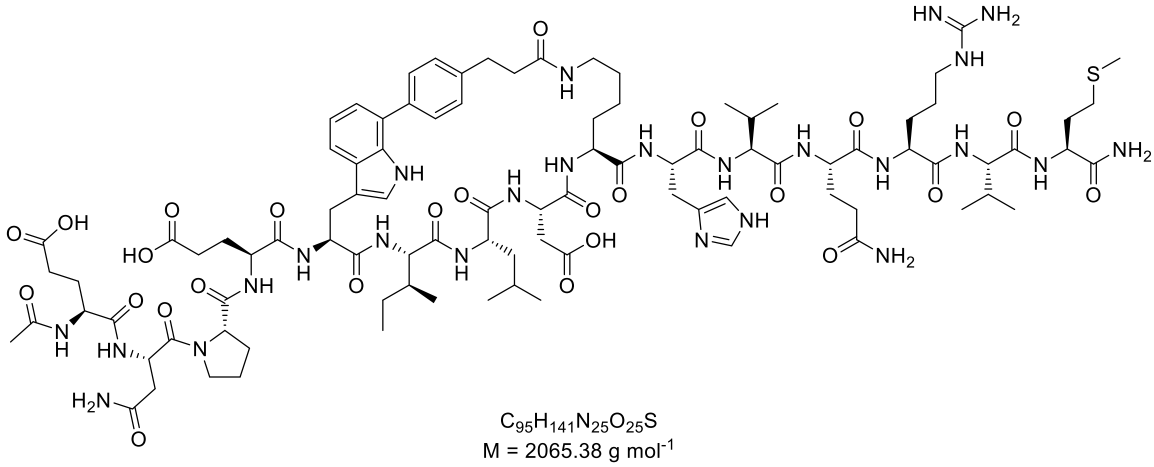

$C_{95}H_{141}N_{25}O_{25}S$

M = 2065.38 g mol^{-1}

M x TFA = 2179.40 g mol^{-1}

The synthesis was performed following the general procedures on a 50 µmol scale. Peptide **P5** (15.7 mg, 14% overall isolated yield referring to initial resin loading) was obtained as colourless solid after purification by preparative RP-HPLC (method A). **RP-HPLC:** t_R = 5.3 min (peptide **5.1**), t_R = 5.5 min (peptide **5.2**). **HRMS** (ESI, positive ions): found *m*/*z* = 1033.0148 $[M+2H]^{2+}$; calcd. *m*/*z* = 1033.0198 $[M+2H]^{2+}$; deviation = 4.87 ppm.

Fraction 1 obtained from preparative RP-HPLC purification (peptide **P5.1**)

Amount: 3.9 mg of 15.7 mg.

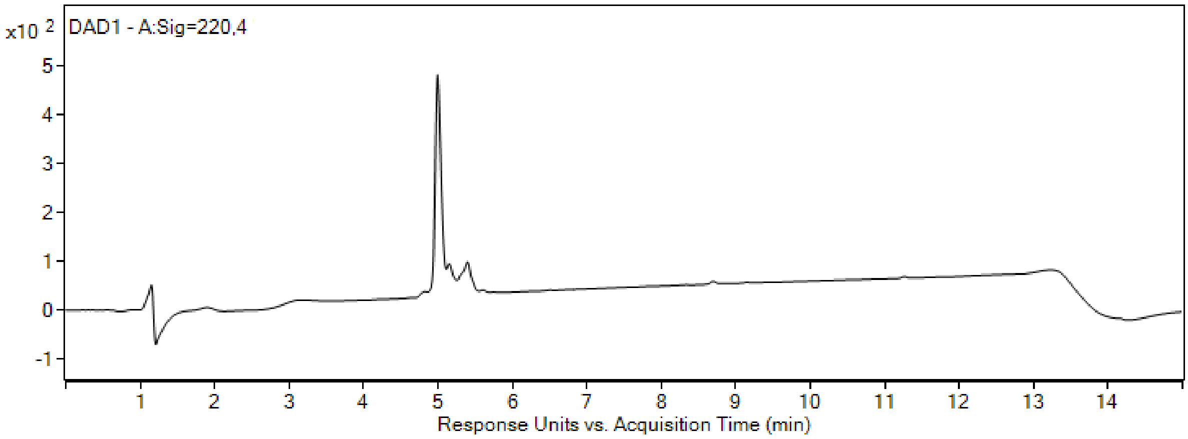

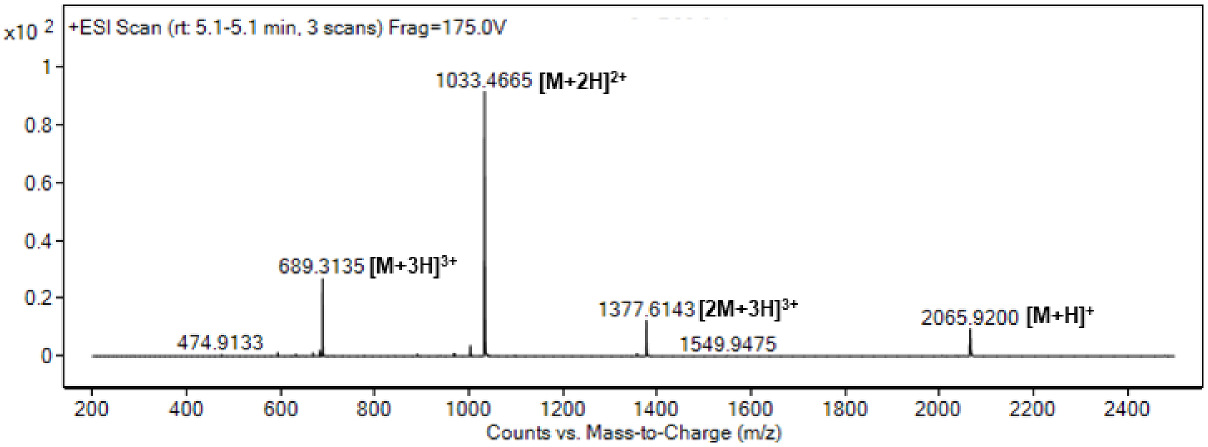

Fraction 2 obtained from preparative RP-HPLC purification (peptide **P5.2**)

Amount: 11.8 mg of 15.7 mg.

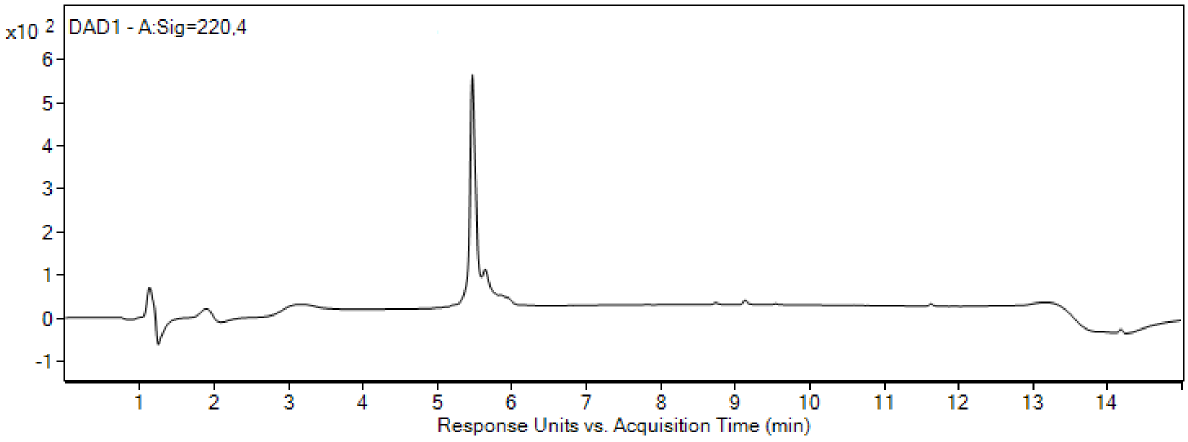

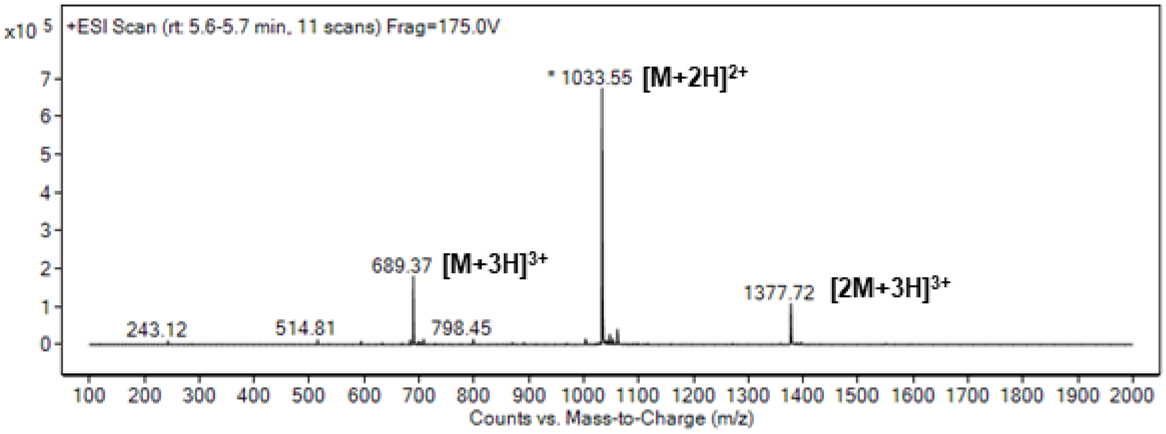

Linear peptides

Peptide **P6**

The synthesis was performed following the general procedures on a 33 µmol scale. Peptide **P6** (17.1 mg, 24% overall isolated yield referring to initial resin loading) was obtained as colourless solid after purification by preparative RP-HPLC (method A). **RP-HPLC:** t_R = 4.7 min. **HRMS** (ESI, positive ions): found m/z = 967.9947 $[M+2H]^{2+}$; calcd. m/z = 967.9989 $[M+2H]^{2+}$; deviation = 4.34 ppm.

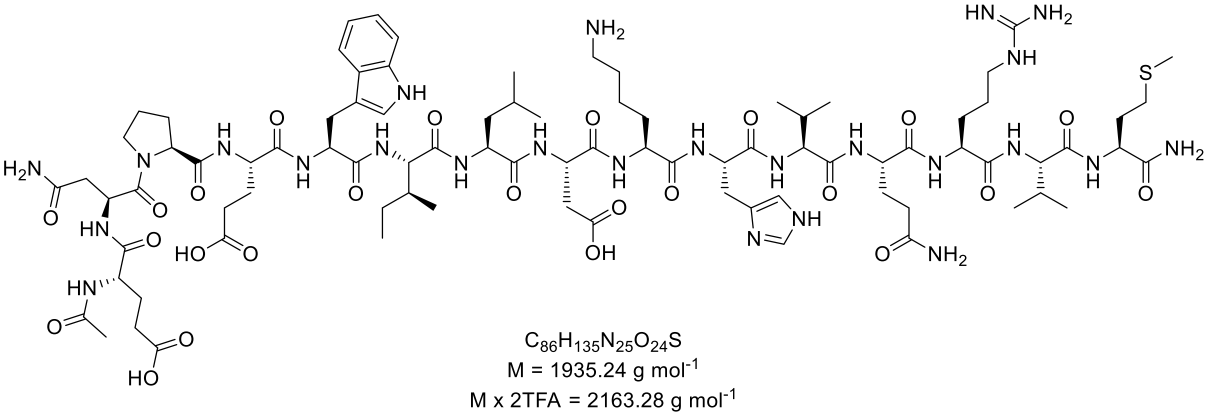

$C_{86}H_{135}N_{25}O_{24}S$
M = 1935.24 g mol^{-1}
M x 2TFA = 2163.28 g mol^{-1}

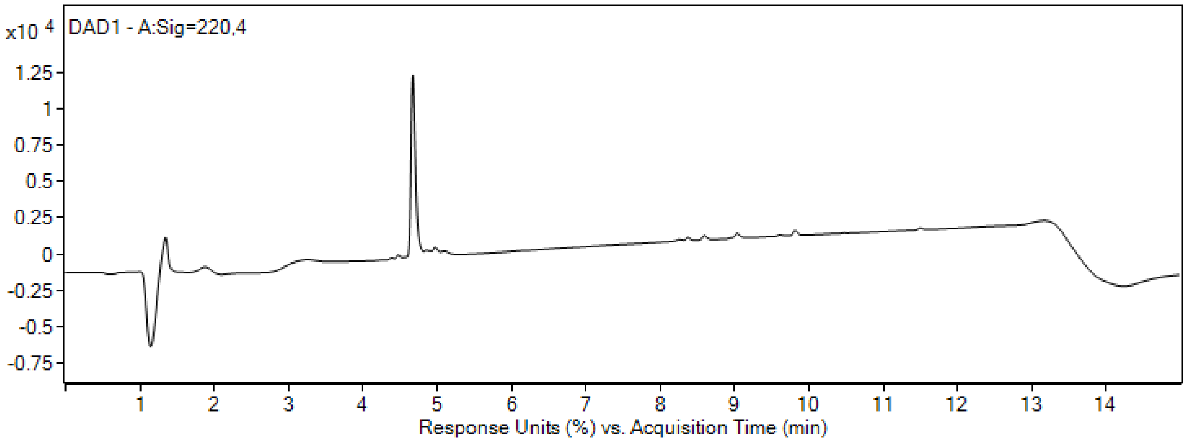

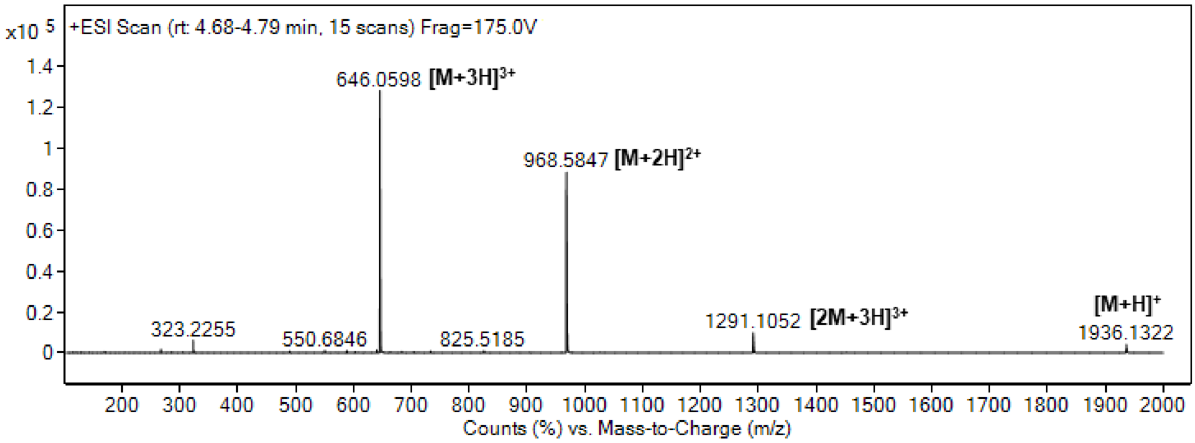

Peptide ***a*AxWt**

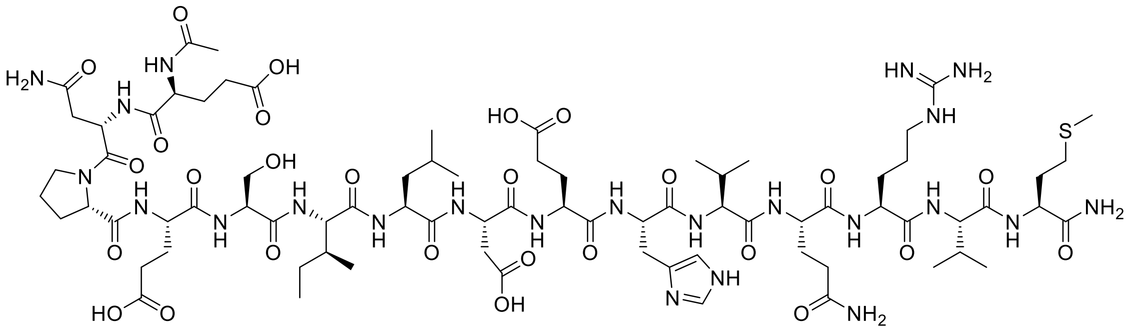

$C_{77}H_{125}N_{23}O_{27}S$
M = 1837.04 g mol^{-1}
M x TFA = 1951.06 g mol^{-1}

The synthesis was performed following the general procedures on a 100 µmol scale. Peptide ***a*AxWt** (9.0 mg, 5% overall isolated yield referring to initial resin loading) was obtained as colourless solid after purification by preparative RP-HPLC (method A). **RP-HPLC:** t_R = 4.7 min. **HRMS** (ESI, positive ions): found *m/z* = 918.9466 [M+2H]$^{2+}$; calcd. *m/z* = 918.9491 [M+2H]$^{2+}$; deviation = 2.70 ppm.

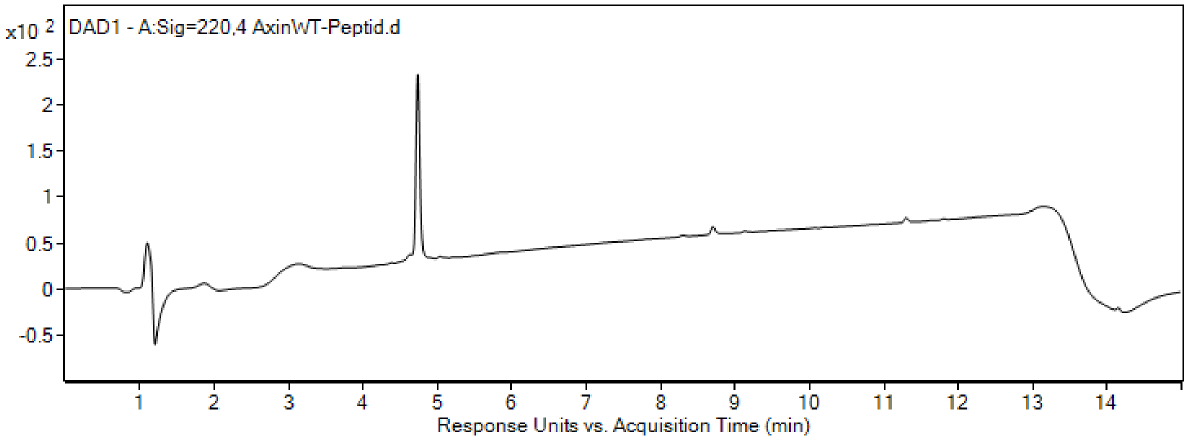

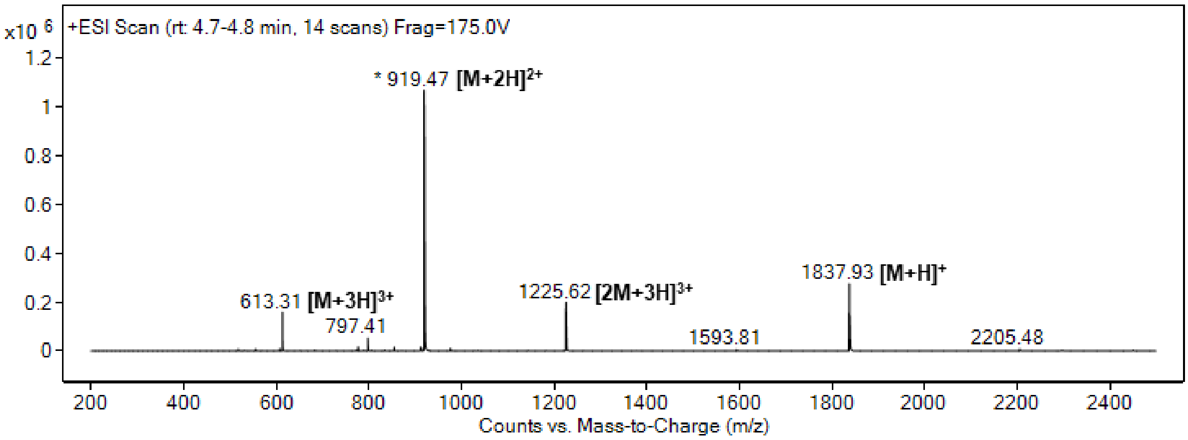

<u>Peptide ***f*AxWt**</u>

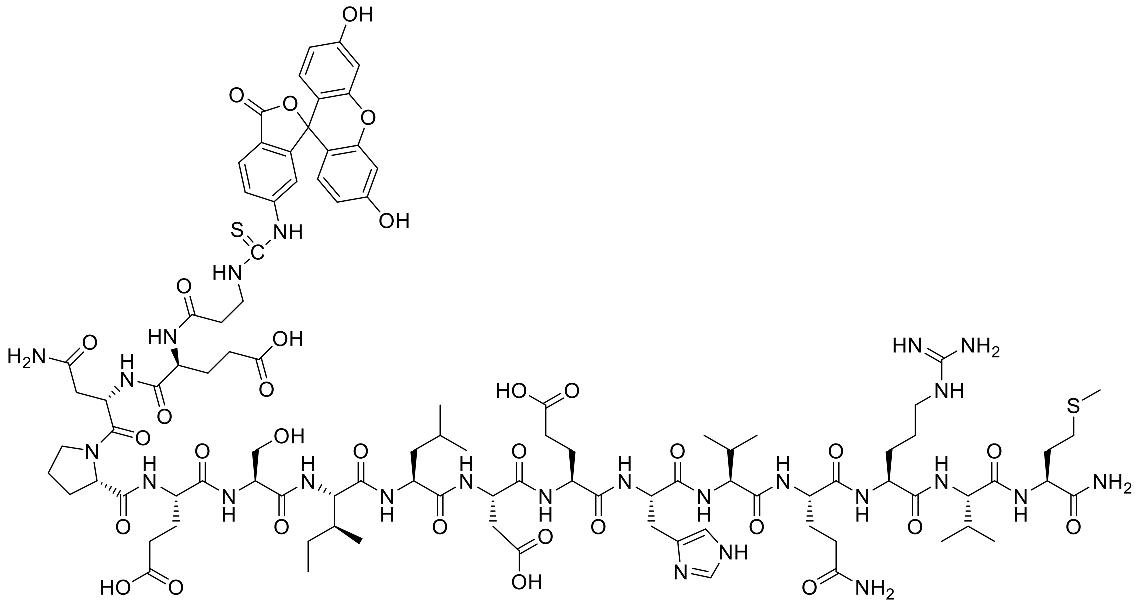

$C_{99}H_{139}N_{25}O_{32}S_2$
M = 2255.46 g mol^{-1}
M x TFA = 2369.48 g mol^{-1}

The synthesis was performed following the general procedures on a 33 µmol scale. Peptide ***f*AxWt** (43.3 mg, 55% overall isolated yield referring to initial resin loading) was obtained as yellow solid after purification by preparative RP-HPLC (method A). **RP-HPLC:** t_R = 5.2 min. **HRMS** (ESI, positive ions): found *m*/*z* = 1127.9758 [M+2H]$^{2+}$; calcd. *m*/*z* = 1127.9803 [M+2H]$^{2+}$; deviation = 3.95 ppm.

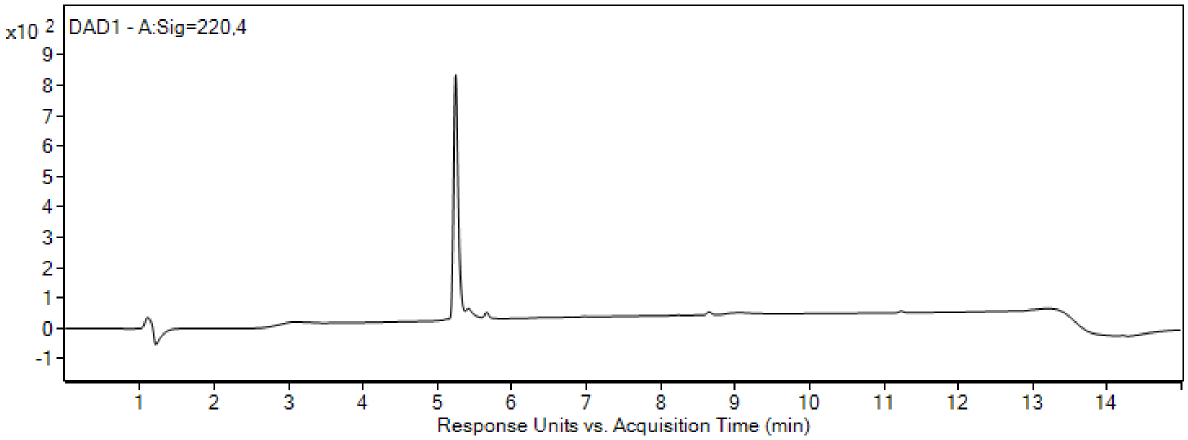

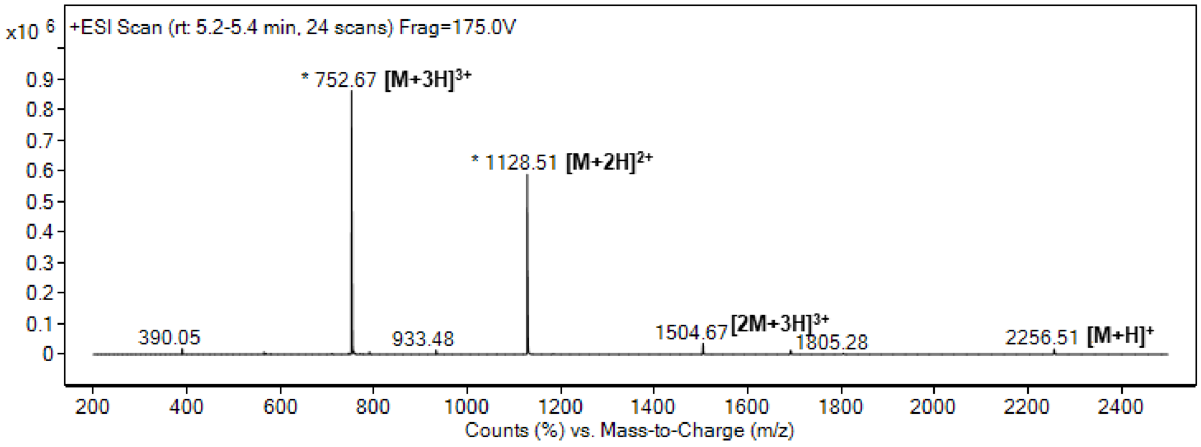

RCM-stapled peptides

Peptide *a*StAx-3

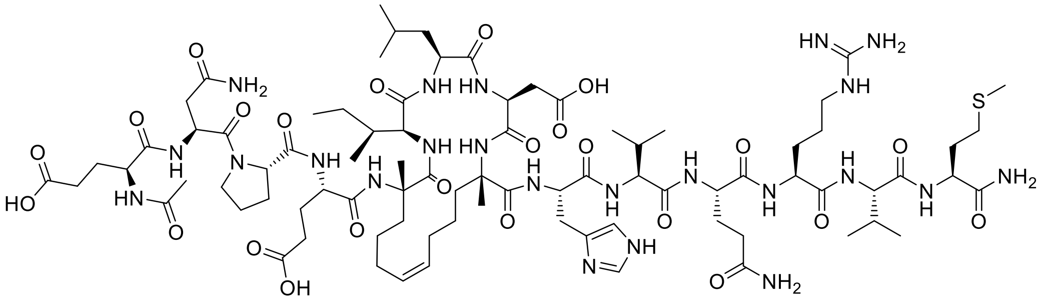

$C_{83}H_{135}N_{23}O_{24}S$
M = 1871.19 g mol^{-1}
M x TFA = 1985.21 g mol^{-1}

The synthesis was performed following the general procedures on a 50 µmol scale. Peptide ***a*StAx-3** (30.6 mg, 31% overall isolated yield referring to initial resin loading) was obtained as colourless solid after purification by preparative RP-HPLC (method A). **RP-HPLC:** t_R = 5.1 min. **HRMS** (ESI, positive ions): found *m/z* = 935.9949 $[M+2H]^{2+}$; calcd. *m/z* = 935.9958 $[M+2H]^{2+}$; deviation = 0.99 ppm.

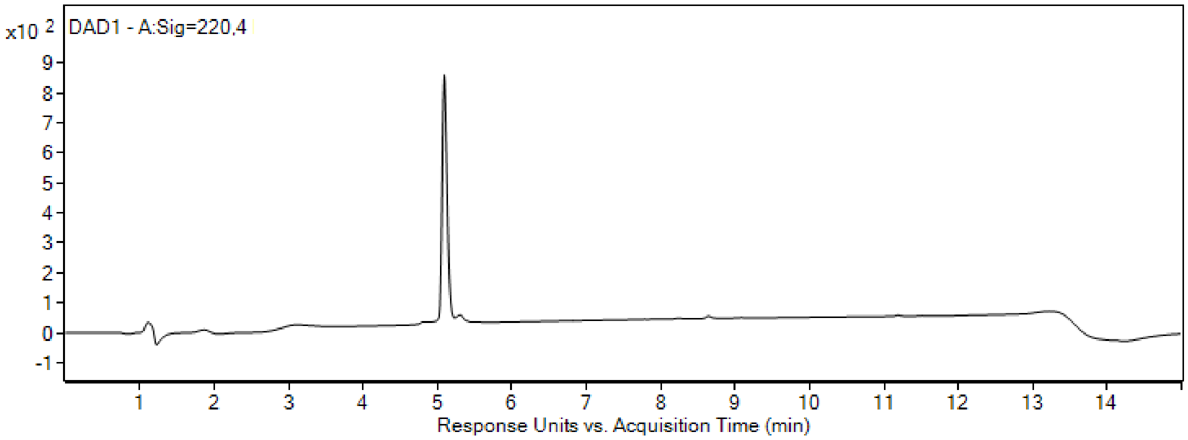

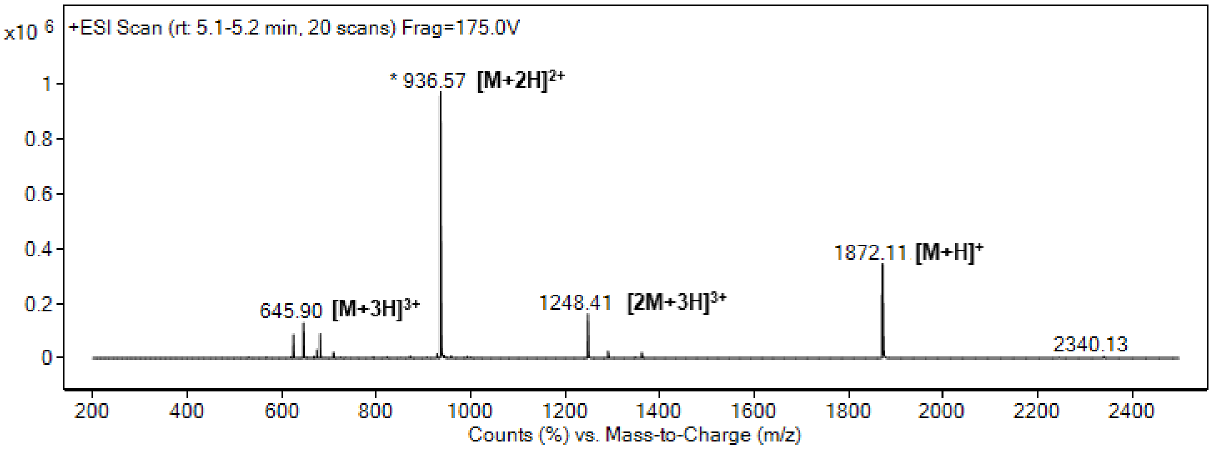

Peptide ***f*****StAx-3**

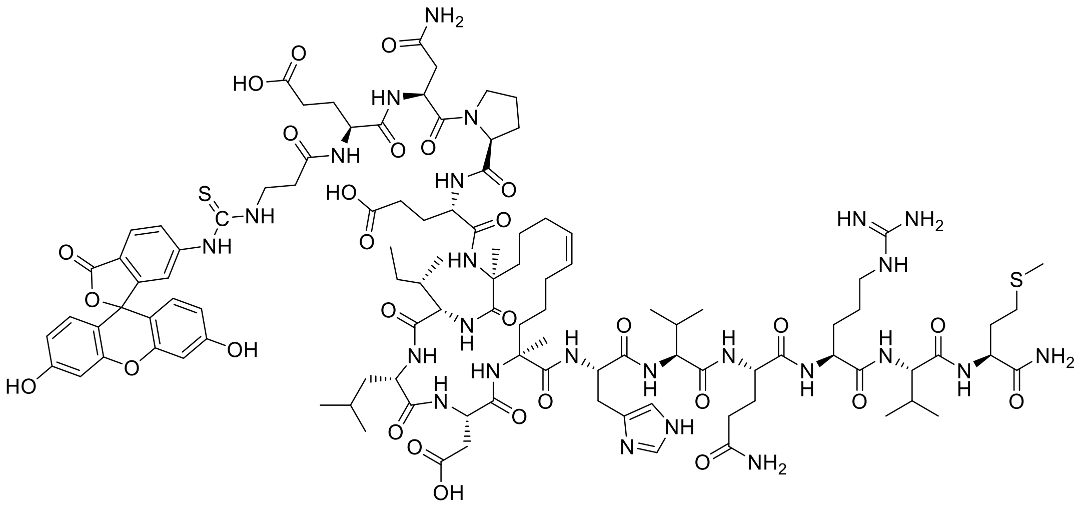

$C_{105}H_{149}N_{25}O_{29}S_2$
M = 2289.61 g mol^{-1}
M x TFA = 2403.63 g mol^{-1}

The synthesis was performed following the general procedures on a 50 µmol scale. Peptide ***f*****StAx-3** (35.0 mg, 29% overall isolated yield referring to initial resin loading) was obtained as yellow solid after purification by preparative RP-HPLC (method A). **RP-HPLC:** t_R = 5.5 min. **HRMS** (ESI, positive ions): found *m*/*z* = 1145.0296 $[M+2H]^{2+}$; calcd. *m*/*z* = 1145.0270 $[M+2H]^{2+}$; deviation = 2.27 ppm.

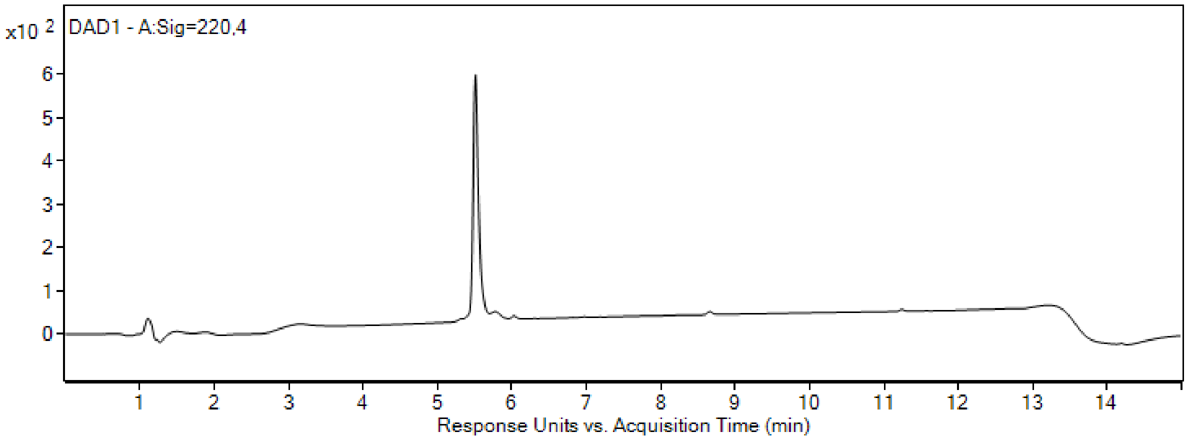

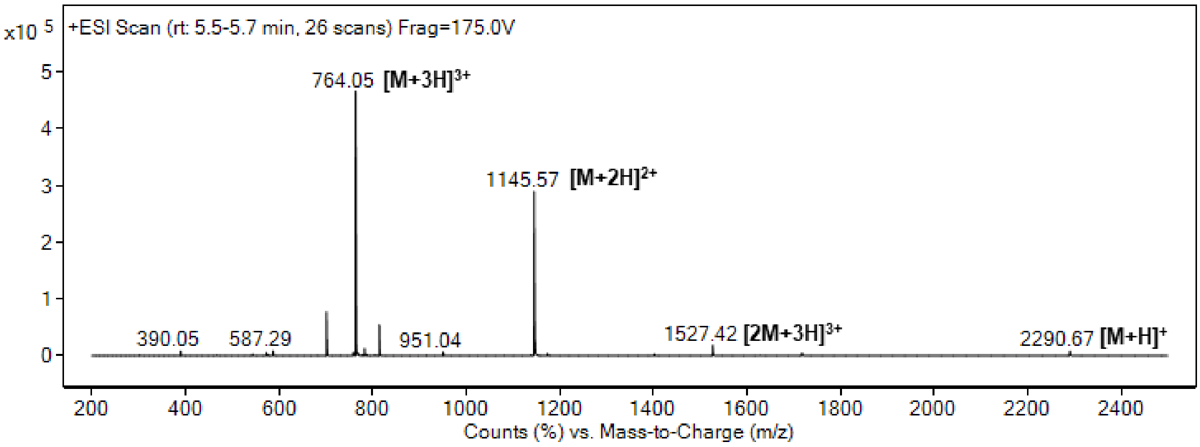

Circular dichroism

CD spectra were recorded using a Jasco J-810 CD spectrometer. Samples were prepared as 100 µM solutions in water and measured in a quartz cuvette at 20 °C. Data points were recorded between 250 and 190 nm in a resolution of 0.1 nm at a scanning rate of 50 nm min^{-1}. Each spectrum is an average of three accumulations. The measured ellipticity at 222 nm (θ_{222}) was converted into the molar ellipticity $[\theta]_{222}$ (deg cm^2 $dmol^{-1}$). The content of α-helix was calculated by the following equations as previously reported:[8a]

$$\text{Helicity} = 100 * (\text{MRE}/\text{MRE}_{max})$$

$$\text{MRE}_{max} = -40000 \cdot (1 - 2.5/n) + 100 \cdot T$$

$$= -31333 \text{ deg cm}^2 \text{ dmol}^{-1}$$

With MRE_{max} = maximum mean residue ellipticity, n = 15 (number of amino acids), T = 20 °C

Table S1: Calculated contents of α-helix.

Peptide	θ_{222} [mdeg]	$[\theta]_{222}$ [deg cm^2 dmol^{-1}] a)	MRE [deg cm^2 dmol^{-1}] b)	α-Helix content [%]
**P5.1**	-7.04	-70400	-4693	15
**P5.2**	-10.02	-100200	-6680	21
**aStAx-3**	-16.19	-161900	-10793	34 (51)c)
**P6**	-5.94	-59400	-3960	13
**aAxWt**	-4.29	-42900	-2860	9 (15)c)

a) $[\theta] = \theta / (c * 10 * l)$ with $c = 10^{-4}$ mol/L, l = 0.1 cm; b) MRE = $[\theta] / n$; c) in brackets literature values for FITC-labelled analogues, ref: Grossmann, T. N.; Yeh, J. T.-H.; Bowman, B. R.; Chu, Q.; Moellering, R. E.; Verdine, G. L. *Proc. Natl. Acad. Sci. U. S. A.* **2012,** *109*, 17942–17947.

For TFE titration experiments, peptide solutions in a mixture of TFE/H_2O 4:1 were additionally measured.

Reference peptides (linear and RCM stapled peptides)

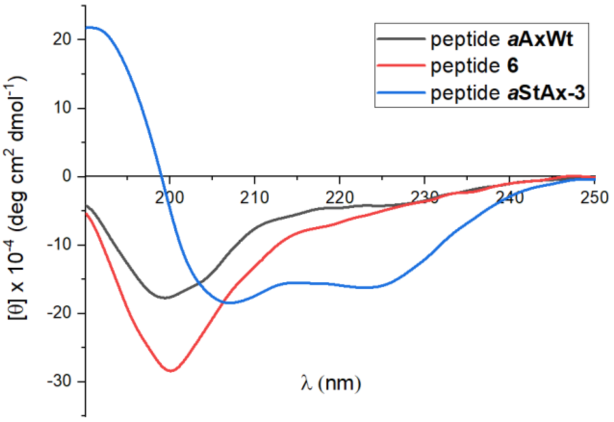

Figure S2: CD spectra of reference peptides **P6**, ***a*AxWt** and ***a*StAx-3**, c = 100 μM in H_2O.

SMC stapled peptides

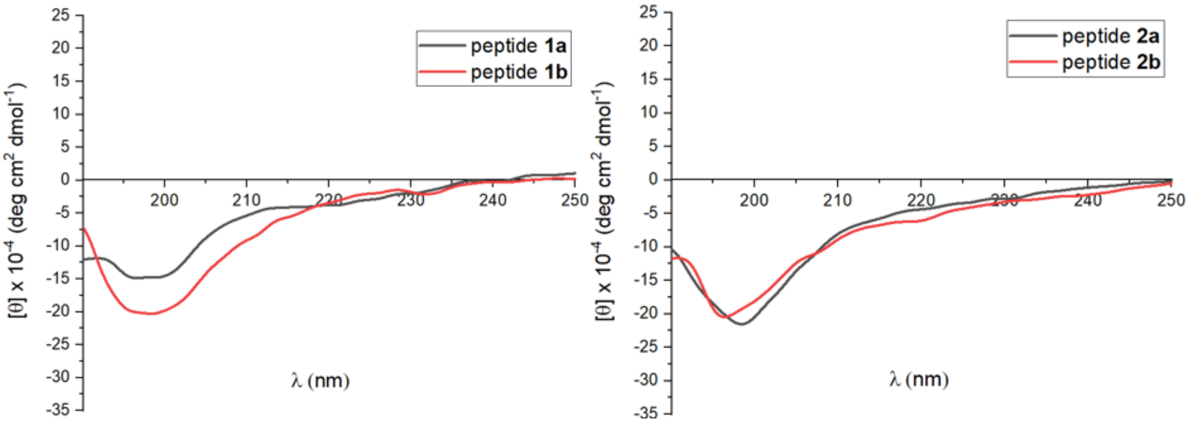

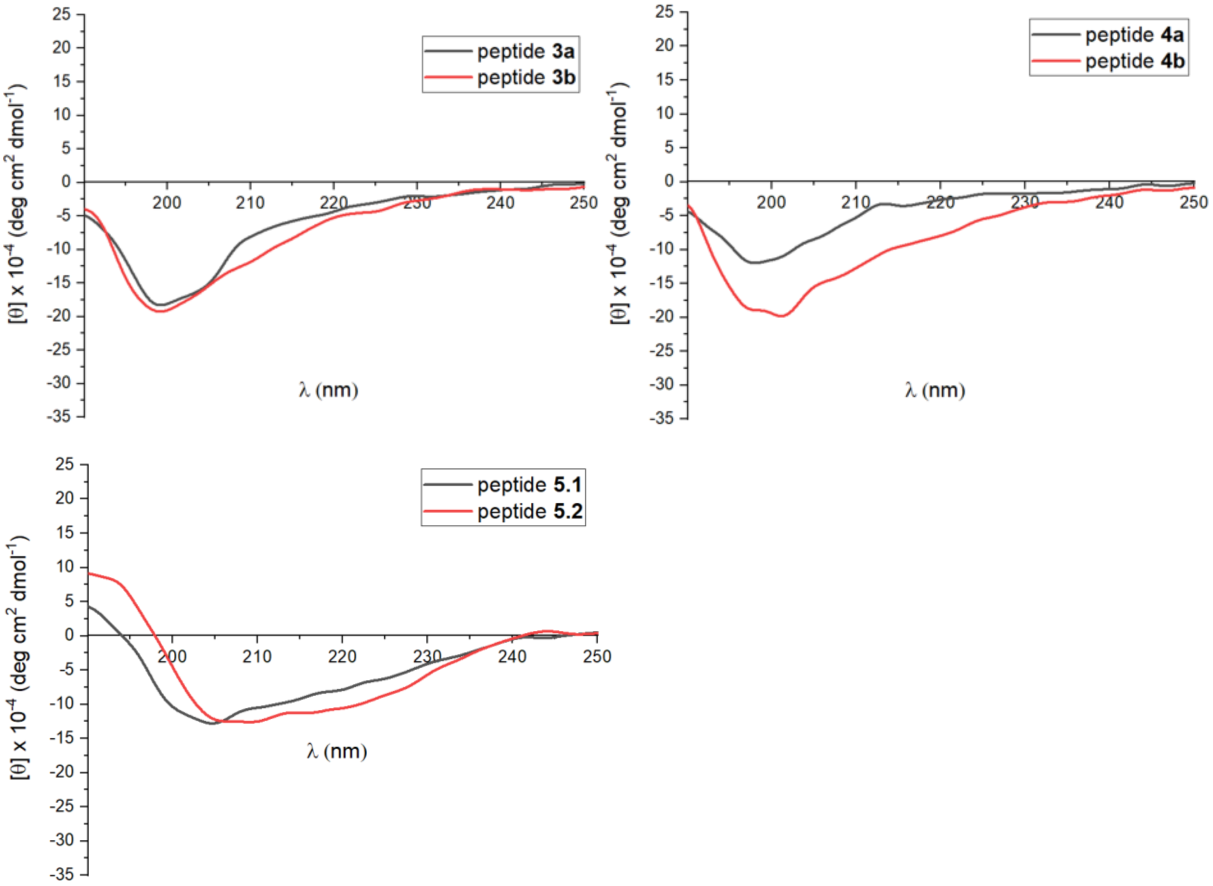

Figure S3: CD spectra of SMC stapled peptides **P1** to **P5**, *c* = 100 µM in H_2O. For peptide **P5**, two isomers were separated by RP-HPLC (peptides **P5.1** and **P5.2**).

TFE titration experiments

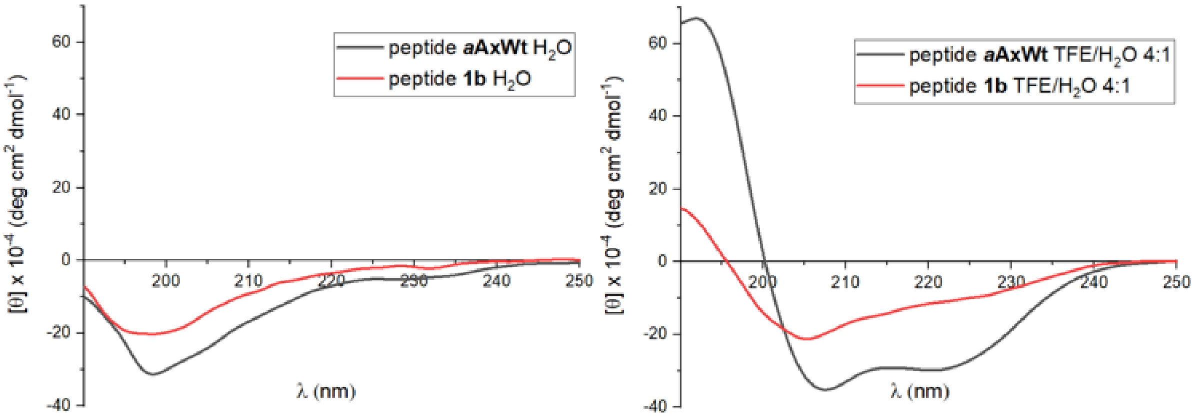

Figure S4: CD spectra of reference peptide ***a*AxWt** and stapled peptide **P1b**, c = 100 µM in H_2O (left) and TFE/H_2O 4:1 (right), respectively. As can be seen, recording CD spectra of the peptides in a mixture of TFE/H_2O 4:1 induces a nice and typical curve progression of a α-helix for the wild type sequence of the CBD domain ***a*AxWt**. The helicity of stapled peptide **P1b** is also increased; however, compared to ***a*AxWt** the curve progression is significantly flatter indicating that the introduction of the cross-link forces the peptide in a more distorted structure.

β-Catenin expression and purification

For fluorescence polarisation assay β-Catenin was expressed and purified according to literature protocols.[9] The plasmid M57 pPET28a-TEV-full-length was transformed into BL21(DE3) competent *E. coli*. An overnight preculture (37 °C, 180 rpm) in LB medium was used to inoculate a culture in 1.5 L TB medium containing 50 µg mL^{-1} kanamycin. This culture was grown (37 °C, 180 rpm) up to an OD_{600} of 1. Protein expression was then induced with isopropyl-β-D-thiogalactopyranoside (IPTG) (0.4 mM) and the cells were cultivated at 18 °C, 180 rpm overnight. Afterwards, cells were harvested by centrifugation (3220 × g, 30 min), washed with 100 mM Na_2HPO_4 buffer (pH 7.4) and stored at −20 °C. For protein purification, cells from cultivation were thawed and suspended in 30 mL lysis buffer (50 mM Tris, 300 mM NaCl, 10 mM imidazole, 1 mM β-mercaptoethanol, 5% (v/v) glycerin, pH 8.0). The cell membrane of bacteria was disrupted by French press for three times at a pressure of 1000 psi. Cell debris was separated from soluble protein by centrifugation at 15.000*g* for 30 min at 4°C. The supernatant was filtrated and applied to a Protino Ni-NTA column for purification. After washing with 30 CV washing buffer (50 mM Tris, 300 mM NaCl, 25 mM imidazole, 1 mM β-mercaptoethanol, 5% (v/v) glycerin, pH 8.0), the protein was eluted with an elution buffer (50 mM Tris, 300 mM NaCl, 250 mM imidazole, 1 mM β-mercaptoethanol, 5% (v/v) glycerin, pH 8.0). Finally, eluted protein was concentrated to 14.9 mg mL^{-1} in storage buffer (20 mM Tris, 150 mM NaCl, 5% (v/v) glycerol, 5 mM β-mercaptoethanol, pH 8.0) using an Amicon Ultra centrifugal filter unit (MWCO 30 kDa). The final sample is centrifuged at 16,000*g* (10 min, 4 °C) and stored at 4 °C.

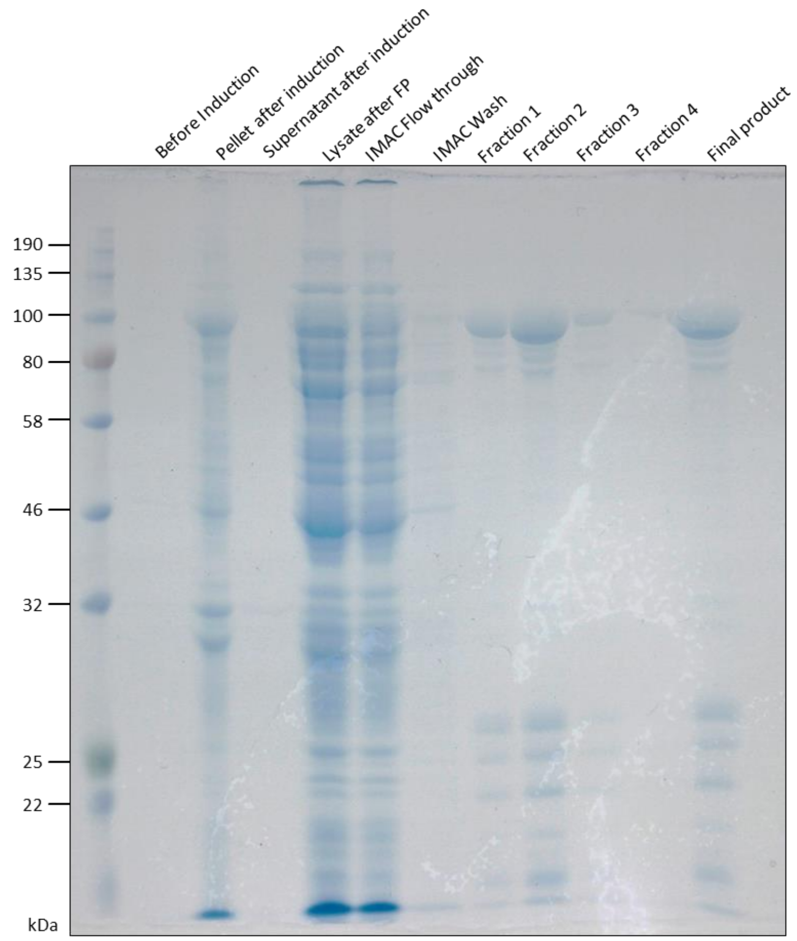

Figure S5: SDS-PAGE analysis (10%) of expression and purification of full-length β-catenin. The band below 100 kDa (NEB Prestained marker) indicates successful isolation of β-catenin (89.9 kDa).

Direct fluorescence polarisation assay

A stock solution of the FITC-labelled peptide in DMSO (20 µM) was diluted to 40 nM in FP buffer (1× PBS with 0.05% Tween20, pH 7.4). A β-Catenin stock with a concentration of 33.3 µM was serially diluted (2.5-fold) in FP buffer. FITC-labelled peptide (5 µL) and β-Catenin (15 µL) dilutions were added to a 384-well plate (BRANDplates®, 384-well, pureGrade™, black). The plate was incubated at 4 °C for 1 h before centrifugation at 1000*g*, 2 min. Fluorescence polarisation was measured using a Tecan Spark 10M with λ_{ex} = 485/20 nm and λ_{ex} = 525/20 nm. Each sample was measured as biological duplicate and technical triplicate. Polarisation was converted into anisotropy and analysis of experimental data was performed in Origin2019 for the normalised values. The 1:1 binding model equation is shown below with r as anisotropy, r_0 as anisotropy of the free peptide, r_b as anisotropy of the β-Catenin:FITC-labelled peptide, K_d as dissociation constant, $[L]_t$ as total labelled ligand concentration and $[P]_t$ as total protein concentration. Equations for all calculations were described in detail by Roehrl *et al.*[10]

$$r = r_0 + (r_b - r_0)\frac{K_d + [L]_t + [P]_t - \sqrt{(K_d + [L]_t + [P]_t)^2 - 4[L]_t[P]_t}}{2[L]_t}$$

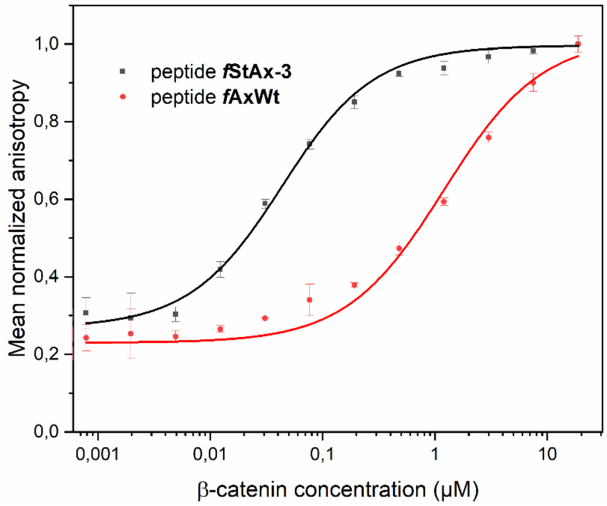

Figure S6: Direct titration curve of fluorescence polarisation assay of FITC-labelled AxWt (***f*AxWt**) and FITC-labelled RCM-stapled peptide StAx-3 (***f*StAx-3**). Dissociation constants were determined to be K_d = 1191 ± 182 nM for ***f*AxWt** and K_d = 63 ± 6 nM for ***f*StAx-3**. ***f*StAx-3** was used as tracer peptide for competitive fluorescence polarisation assay (see below).

Competitive fluorescence polarisation assay

A stock solution of the FITC-labelled peptide in DMSO (20 µM) was diluted to 40 nM in FP buffer (1× PBS with 0.05% Tween20, pH 7.4). A β-Catenin stock solution with a concentration of 6 µM was prepared in FP buffer. Stock solutions of non-labelled peptides (1 mM) in DMSO were serially diluted (2.5-fold) in FP buffer. Dilutions of peptides (5 µL), FITC-labelled tracer peptide (5 µL, 10 nM final) and β-catenin (10 µL, 3 µM final) were added to a black 384-well plate. The plate was incubated at 4 °C for 1 h before centrifugation at 1000*g*, 2 min. Fluorescence polarisation was measured using a Tecan Spark 10M with λ_{ex} = 485/20 nm and λ_{ex} = 525/20 nm. Each sample was measured as biological duplicate and technical triplicate. Polarisation was converted into anisotropy and analysis of experimental data was performed in Origin2019 for the normalised values.

The equation used for K_d determination of unlabeled peptides is shown below with r as anisotropy, r_0 as anisotropy of the free peptide, r_b as anisotropy of the β-Catenin:FITC-labelled tracer peptide, K_{d2} as dissociation constant between the non-labelled ligand and the protein, $[L]_t$ as total non-labelled ligand concentration and $[L]_{st}$ as total FITC-labelled peptide concentration. K_{d1} is the apparent K_d that was determined in direct fluorescence polarisation assays for the labelled-tracer peptide. Equations for all calculations were described in detail by Roehrl *et al.*[10]

$$r = r_0 + (r_b - r_0)\frac{2\sqrt{(d-3e)}\cos\left(\frac{\theta}{3}\right) - d}{3K_{d1} + 2\sqrt{(d-3e)}\cos\left(\frac{\theta}{3}\right) - d}$$

$$d = \ K_{d1} + K_{d2} + [L]_{st} + [L]_t - [P]_t$$

$$e = \ ([L]_t - [P]_t)K_{d1} + ([L]_t - [P]_t)K_{d2} + K_{d1}K_{d2}$$

$$f = \ -K_{d1}K_{d2}[P]_t$$

$$\theta = \ arcos\left[\frac{-2d^3 + 9de - 27f}{2\sqrt{(d^2 - 3e)^3}}\right]$$

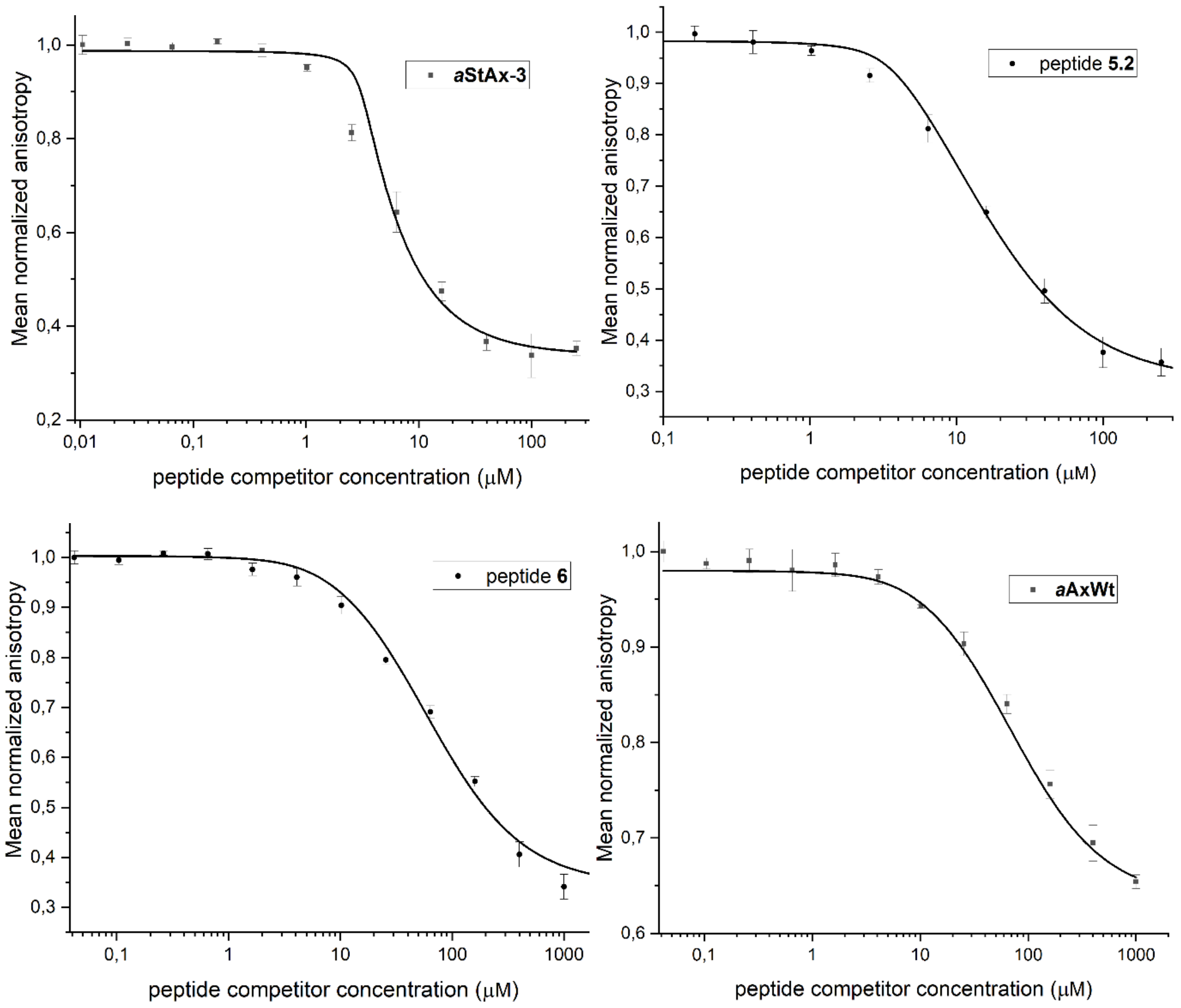

Figure S7: Inhibition curves of competitive fluorescence polarisation assays for unlabelled, linear peptides **P6** and ***a*AxWt** and unlabelled, stapled peptides **P5.1**, **P5.2** and ***a*StAx**, which were titrated against tracer peptide ***f*StAx-3**. Dissociation constants were determined to be K_d = 1448 ± 204 nM for ***a*AxWt**, K_d = 1241 ± 162 nM for **P6**, K_d = 258 ± 43 nM for **P5.2** and K_d = 49 ± 26 nM for ***a*StAx-3**. These reasonable K_d values are confirmed by agreement with those K_d values of references, which were determined by direct fluorescence polarisation assays for the FITC-labelled analogues of **AxWt** and **StAx-3** (K_d = 1191 ± 182 nM for ***f*AxWt** and 63 ± 6 nM for ***f*StAx-3**) as control (see section Direct fluorescence polarisation assay).

Proteinase K stability assays

The assay was performed using a modified protocol.[11] A 10 mM peptide stock solution was prepared in a mixture of Na_2HPO_4 buffer (100 mM, pH 7.4) and DMSO (9:1). The peptide stock solution (25 µL) was diluted with more Na_2HPO_4 buffer (450 µL) and thoroughly mixed. Proteolytic digestion was started by addition of a solution of Proteinase K (50 µg mL^{-1}, activity 36.9 mAnsonU mg^{-1}) in Na_2HPO_4 buffer (25 µL) and incubated for 120 min at 25 °C (resulting assay concentrations 0.5 mM peptide and 2.5 µg mL^{-1} proteinase K). For LC-MS analysis, a 50 µL sample was diluted with 50 µL of methanol containing 0.1% TFA (v/v) and spun down at 10000*g* and 4 °C for 30 min.

Reaction monitoring via LC-MS

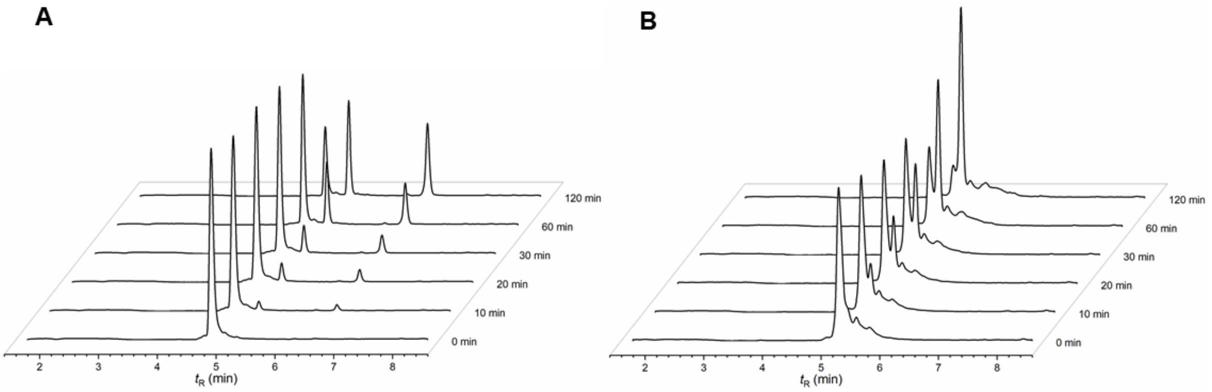

Figure S8: Reaction monitoring via LC-MS at λ = 280 nm. **A**) Linear Peptide **P6**, samples were taken at *t* = 0, 10, 20, 30, 60 and 120 min. After 120 min, peptide **P6** was degraded in three fragments with fragment Ac-ENPEWILDKHVQ-OH eluting at exactly the same retention time than peptide **P6** (see mass spectra below at reaction times 0 and 120 min). For fragment assignments, see mass spectra below. **B**) SMC stapled peptide **P5**, samples were taken at *t* = 0, 10, 20, 30, 60 and 120 min. Compared to linear peptide **P6**, two of three cleaving sites are blocked.

LC-MS mass spectra

Peptide **P6** at *t* = 0 min

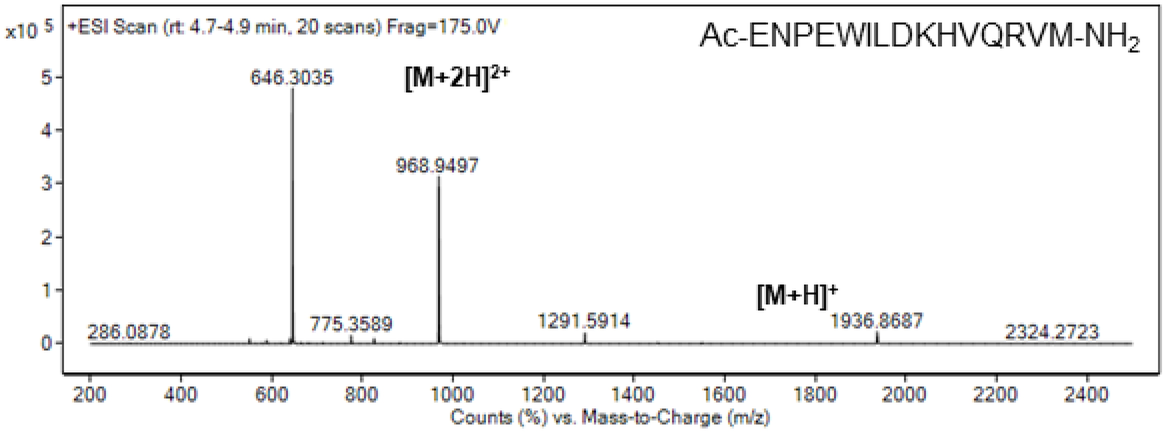

Peptide **P6** at *t* = 120 min

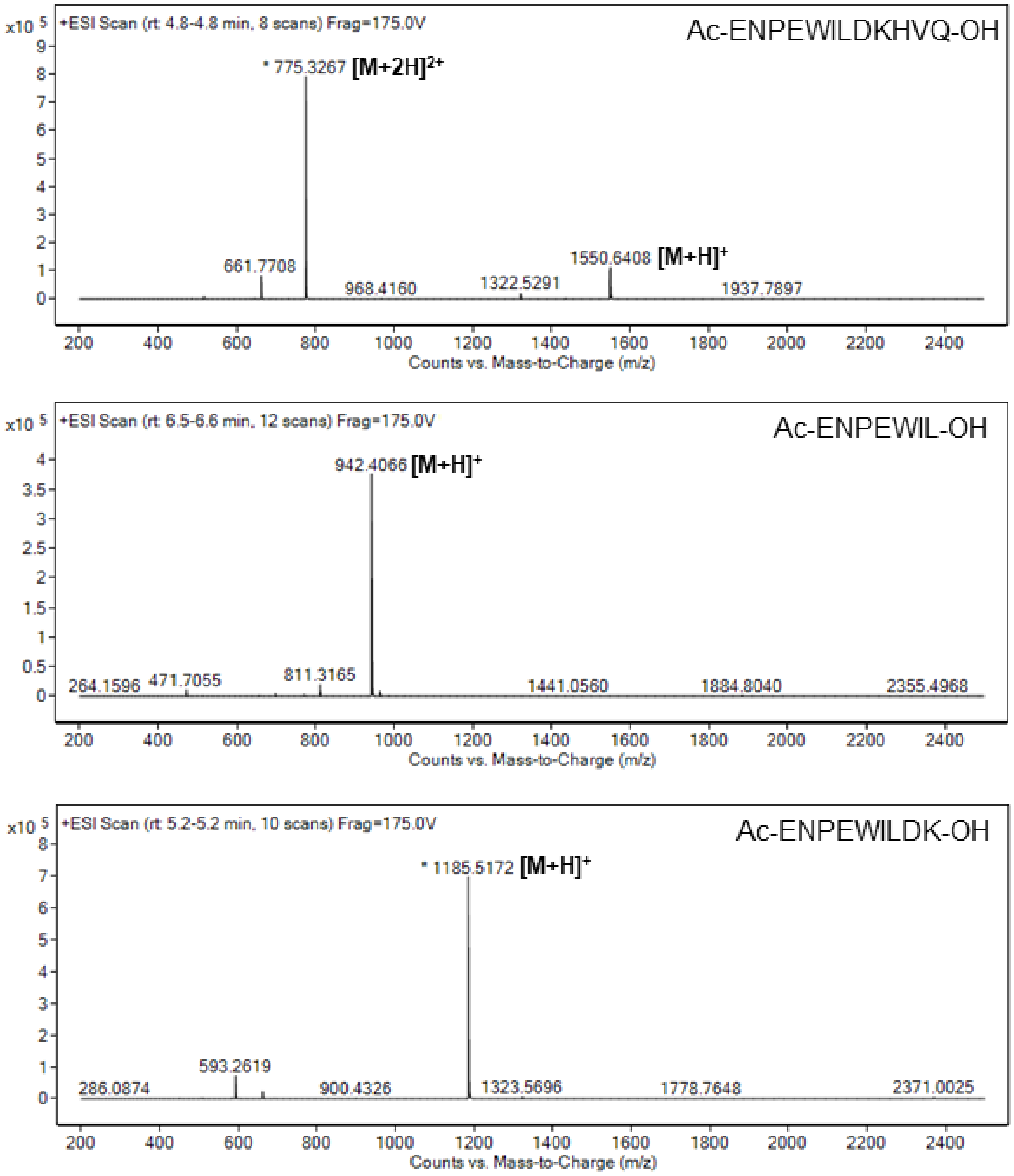

Figure S9: ESI-MS spectra of the different fragments of peptide **P6** digestion by proteinase K. As can be seen in the HPLC and mass spectra, after 120 min the linear peptide **P6** is degraded by Proteinase K, which cleaves at three sites after Leu (fragment at t_R = 6.5 min), Lys (fragment at t_R = 5.1 min) and Gln (fragment at t_R = 4.7 min): Ac-ENPEWI**L**D**K**HV**Q**RVM-NH_2.

Peptide **5** at *t* = 0 min

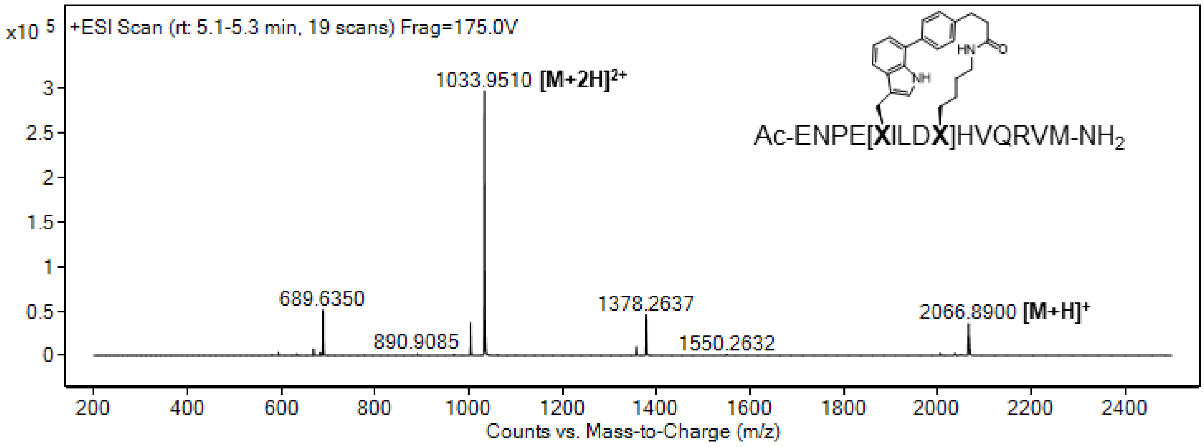

Peptide **5** at *t* = 120 min

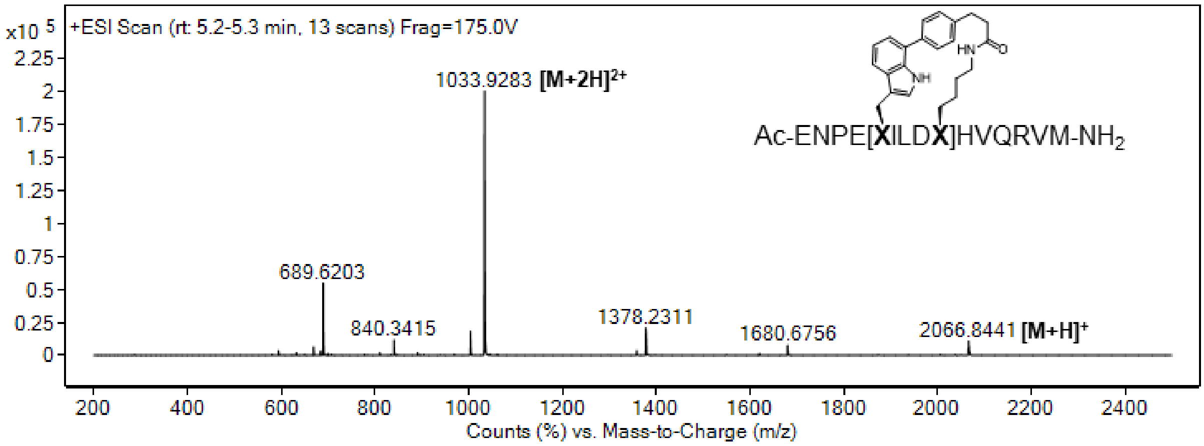

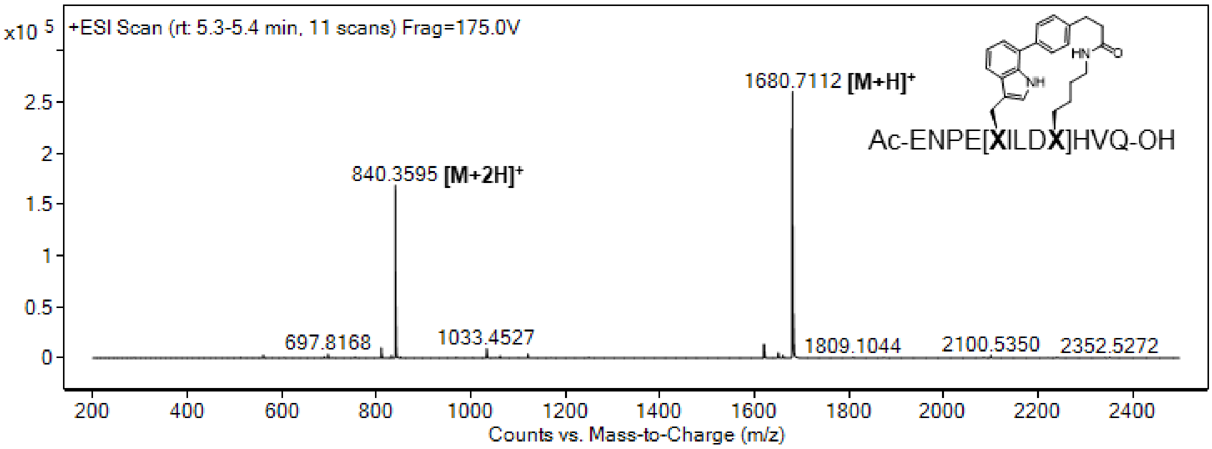

Figure S10: ESI-MS spectra of the stapled peptide **P5** and the only fragment resulting from digestion by Proteinase K. Compared to the linear analogue **P6**, two of three cleaving sites are blocked and Proteinase K is not able to hydrolyse peptide **5** after leucine and lysine anymore due to the macrocycle. Only cleavage after glutamine is still observed (fragment at t_R = 5.3 min).

Density functional theory (DFT) calculations

Calculations have been performed at the density functional theory (DFT) level using Gaussian 09.[12] Standard geometry optimisation was performed using Pople's 6-31g basis set with polarisation functions on hydrogens and heavier elements with the B3LYP functional and the Polarisable Continuum Model (PCM) with dielectric constant of water (i.e., B3LYP/6-31g(d,p)/PCM(water)). The character of the stationary points was assessed by frequency calculations and all structures reported correspond to true minima (i.e., no imaginary frequency). Calculation of the reference structure (peptide **P6**) started from the helical conformation and the resulting minimum structure served as a base to build structures of the proposed derivatives (labelled as prop. X in what follows). Notice that no further exhaustive search for global minimum was performed in these preliminary calculations. The main chain dihedral angles φ(C-Cα-N-C_{-1}) and ψ(N-Cα-C-N_{+1}) characterising the obtained conformations are listed in Table S2.

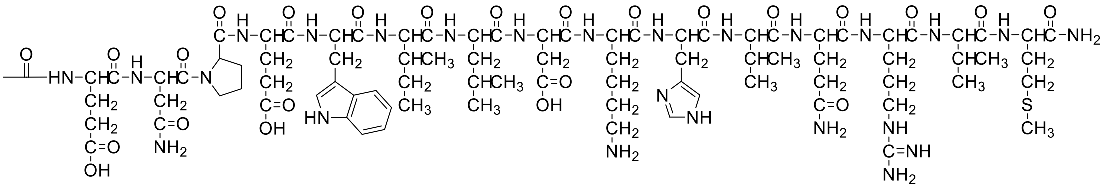

Reference

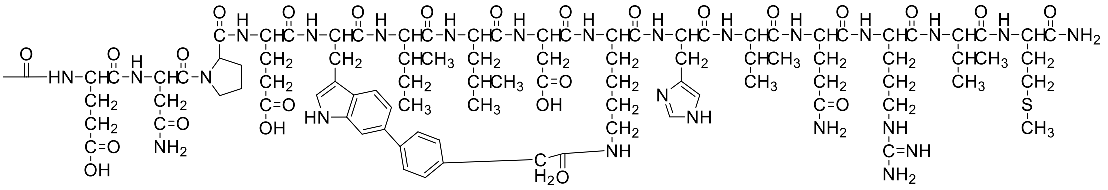

Prop. 1

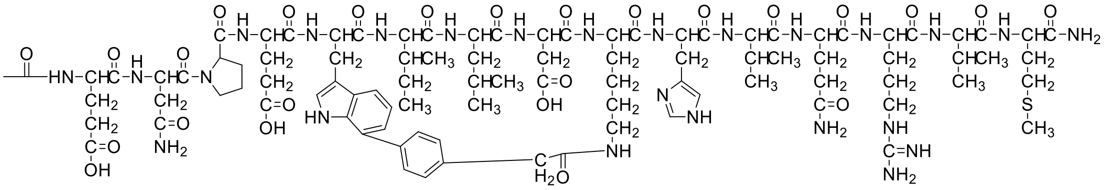

Prop. 2

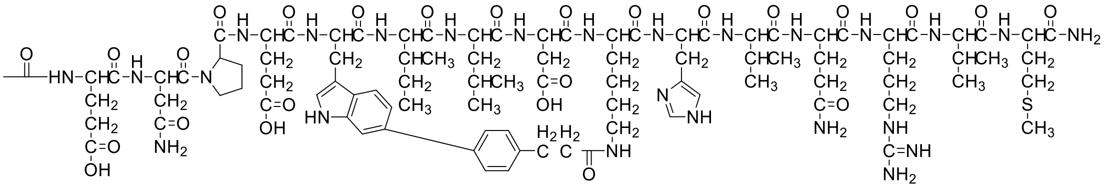

Prop. 3

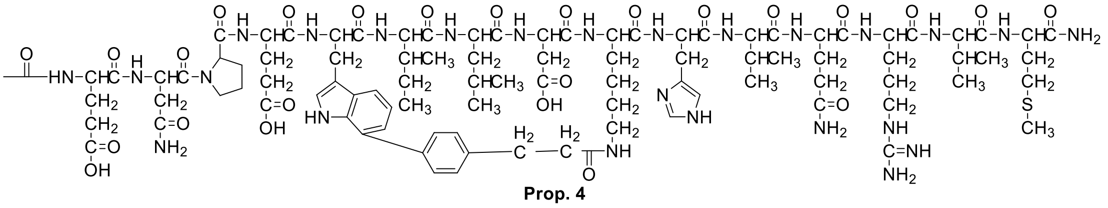

Prop. 4

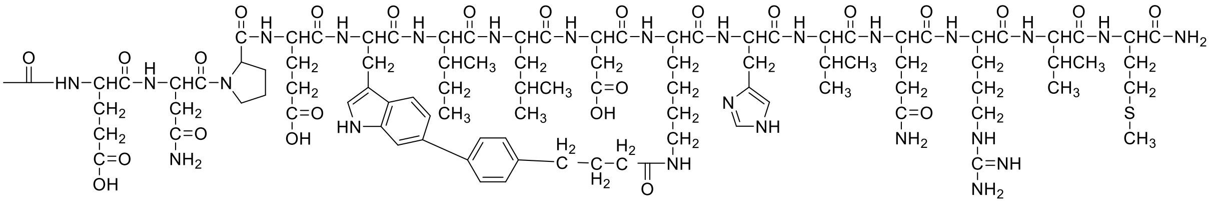

Prop. 3L

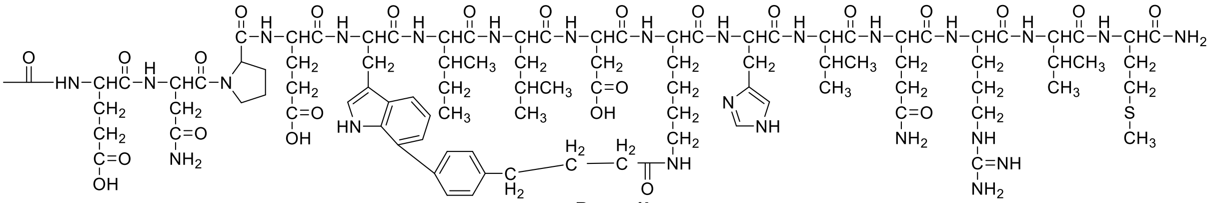

Prop. 4L

Table S2: Main chain torsion angles for the conformations as optimised in this work.

Residue no.	Residue	Dihedral angle types	Dihedral angle values [º]						
			Reference (peptide **P6**)	Prop. 1	Prop. 2	Prop. 3	Prop. 3L	Prop. 4 (peptide **P5**)	Prop. 4L
1	Glu	phi1	80.1	70.7	69.9	70.5	70.5	69.9	69.7
		psi1	38.3	-49.9	-46.0	-51.2	-58.1	-54.4	-56.0
2	Asn	phi2	-105.9	-66.0	-65.8	-66.0	-67.8	-66.3	-63.3
		psi2	-44.6	-50.3	-52.4	-49.7	-44.6	-48.1	-49.3
3	Pro	phi3	-79.8	-57.9	-60.7	-58.0	-56.0	-57.9	-61.9
		psi3	-38.9	-47.9	-41.9	-48.5	-50.5	-49.0	-52.8
4	Glu	phi4	-88.3	-54.7	-59.0	-53.1	-50.1	-53.9	-56.4
		psi4	-39.4	-37.0	-50.2	-43.5	-47.9	-40.9	-46.0
5	Trp	phi5	-67.4	-107.0	-159.8	-90.6	-84.8	-87.2	-94.1
		psi5	-50.0	-23.7	21.3	17.4	-34.1	8.5	4.9
6	Ile	phi6	-99.9	-114.0	-95.7	-103.5	-125.7	-104.2	-85.3
		psi6	-40.9	-59.9	-51.4	-43.1	-46.6	-35.7	-40.3
7	Leu	phi7	-92.0	-92.8	-83.0	-81.8	-68.8	-77.7	-74.1
		psi7	-24.3	53.8	53.7	-25.7	-28.9	-31.9	-32.7
8	Asp	phi8	-63.5	-59.6	-127.6	-41.7	-45.8	-48.3	-49.4
		psi8	-41.6	-41.0	-108.0	-61.7	-50.8	-46.4	-43.2
9	Lys	phi9	-107.4	-66.5	-79.7	-73.2	-70.1	-107.6	-117.9
		psi9	12.3	-13.1	85.6	-7.6	-10.8	32.0	29.2
10	His	phi10	-91.3	-90.4	-91.8	-91.6	-91.7	-58.1	-62.1
		psi10	-29.7	-6.7	-25.1	-12.6	-9.7	-36.8	-34.6
11	Val	phi11	-87.2	-107.7	-65.9	-93.5	-95.8	-64.2	-64.7
		psi11	-42.0	-47.4	-29.5	-41.5	-41.8	-40.0	-40.8
12	Gln	phi12	-79.2	-59.8	-85.6	-59.1	-59.3	-75.4	-72.7

		psi12	-33.5	-38.2	-7.0	-35.9	-36.0	-27.8	-30.2
13	Arg	phi13	-79.7	-76.6	-109.5	-71.1	-71.0	-65.7	-67.7
		psi13	-34.9	-3.8	-54.9	-10.8	-11.2	-33.8	-30.9
14	Val	phi14	-81.3	-87.6	-118.5	-83.5	-82.9	-81.9	-83.2
		psi14	-36.2	-17.9	-95.5	-19.6	-19.7	-49.3	-48.7
15	Met	phi15	-78.5	-85.0	-81.8	-85.2	-85.1	-85.7	-84.9
		psi15	84.6	67.9	91.3	67.0	67.3	70.0	69.1

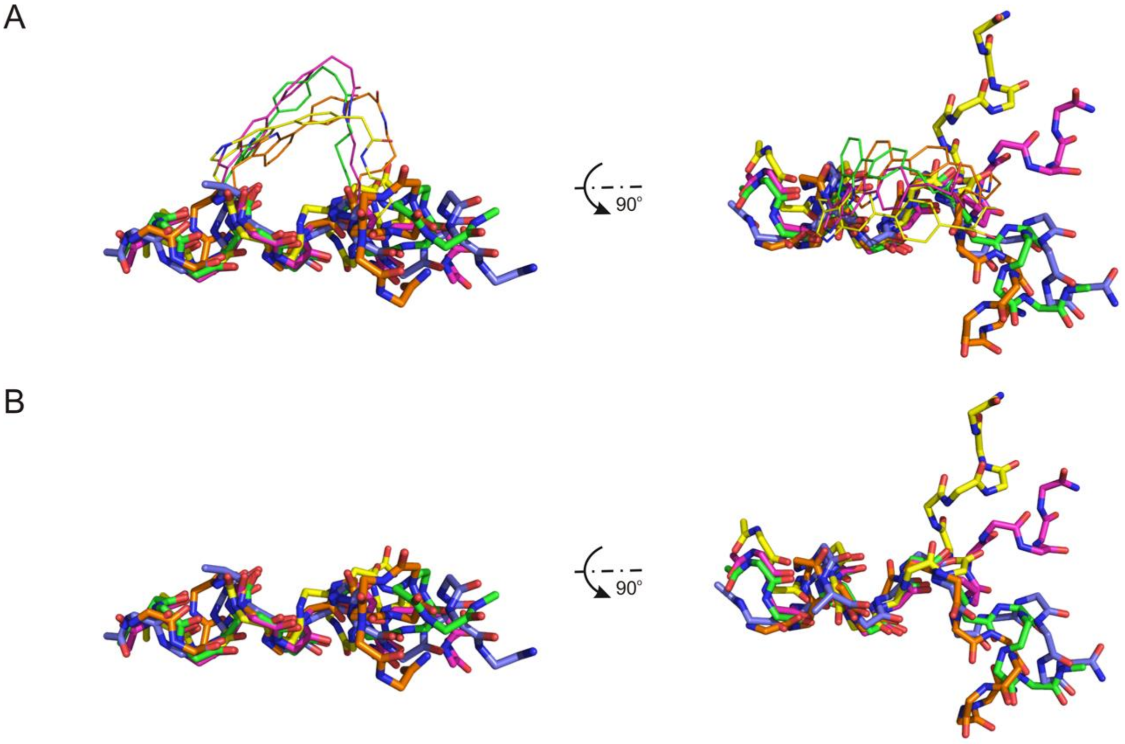

Figure S11: Superimposition of the conformations optimised for the **reference peptide** (referred to as peptide **P6** in the manuscript, violet), **Prop. 1** (yellow), **Prop. 2** (orange), **Prop. 3** (pink) and **Prop. 4** (referred to as peptide **P5** in the manuscript, green). On panel **A)**: main chain and linker moiety; on panel **B)** only main chain.

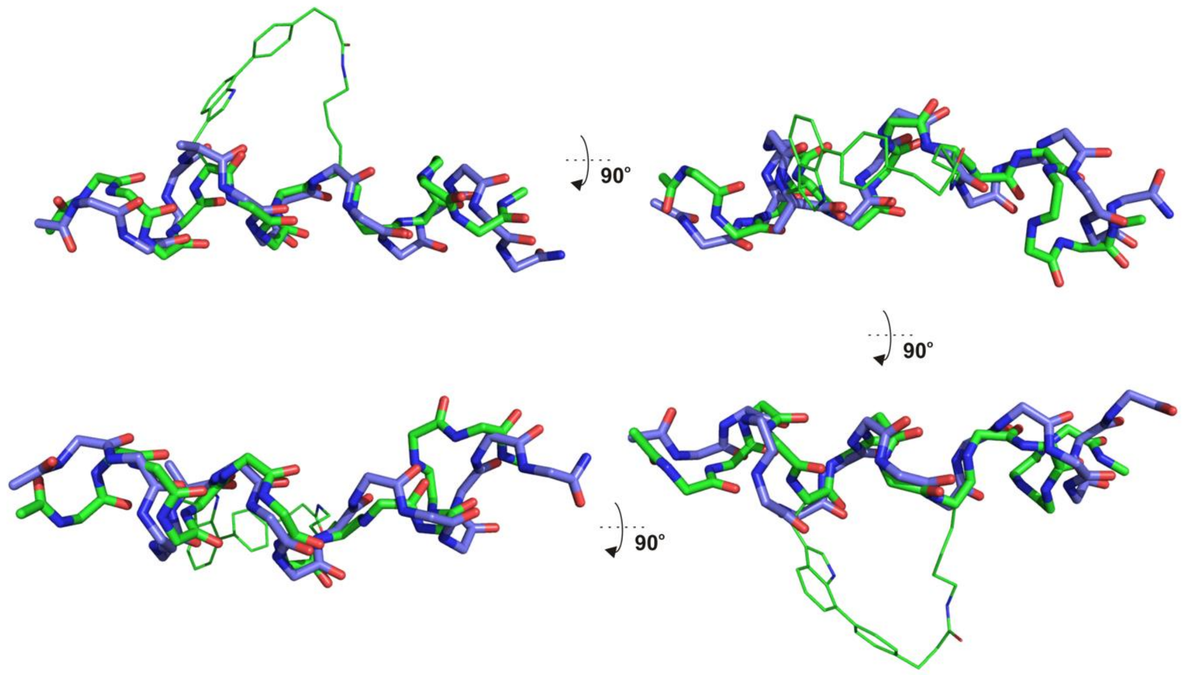

Figure S12: Superimposition of the conformations optimised for **reference peptide** (**P6**, violet) and **Prop. 4** (**P5**, green) shown as sticks (main chain) and lines (linker).

Within the limit of the current calculations, analysis of the optimised structures harbouring different linkers and comparison to the reference peptide (**P6**) tends to indicate that introduction of a linker significantly affects the structure of the peptide. In the case of prop. 1-3, the linker induces a strain that bends the helix, which would necessarily result in a different preferred secondary structure for these derivatives that would in turn reduce their biological activity. Molecule prop. 4, however, shows little to no change in secondary structure compared to the helical form of the reference structure peptide **P6**. The calculations therefore suggest prop. 4 as a potential candidate for enhancing the helical character of the peptide since it does not disrupt the structure of the reference and is short enough to expect a locking of the helical conformation. Prop. 4 was then further considered for synthesis and is referred to as peptide **P5** in the manuscript and in this document. Comparing prop. 1 and 2 as well as prop. 3 and 4 shows that substitution at the C^7 position (prop. 2 and 4) of the indole ring is geometrically more favourable than its counterpart at the C^6 position (prop. 1 and 3). Elongation of the aliphatic part of the linker (prop. 3L and 4L) did not improve the final structure. Because the ultimate goal of the molecular design is to lock the helical structure of the peptide, the shorter linker in prop. 4 (peptide **P5**) was thus preferred over the longer variant of prop. 4L.

Molecular dynamics-based conformational analysis

Molecular dynamics (MD) simulations and analysis were performed within the Amber18/AmberTools 19 modelling suite.[13] All MD runs were done using the CUDA accelerated version of pmemd as implemented in Amber18. Quantum mechanical (QM) calculations for the derivation of molecular mechanics (MM) parameters were performed with the Orca QM program[14] and corresponding in-house scripts.

MM parameters for the modified amino acids (i.e., 7-subsituted tryptophan and modified lysine), where adapted from the ff14SB[15] force filed and from GAFF[16] for the linker. Standard amino acids where modeled with the ff14SB forcefield. Atomic partial charges of the new residues were obtained by following the standard RESP procedure for Amber force fileds, i.e., optimisation and derivation of electrostatic potential (ESP) at the HF/6-31G* level and following a similar procedure as in our earlier work.[17] The parameters for the dihedral angle between the indole and the linker where directly ported from our previous study.[17d] All peptides where capped at their *N*- and *C*-terminal ends with acetyl and *N*-methyl groups, respectively.

The same simulation protocol was applied for all peptides considered in this work. Initial structures of all the peptides were built in an arbitrary conformation and produced using a combination of tleap from the AmberTools19 and Avogadro 1.2. The structures then underwent a simulated annealing (SA) with random initial velocities for 1000 independent runs of 500 ns with a time step of 2 fs and a temperature ramp from 0K to 600K and back to 0K in the gas phase. The 1000 conformations from the SA were then energy minimized and clustered using the hierarchical agglomerative algorithm with an epsilon of 3.0 Å. The representative structures of the four most populated clusters were selected as the starting structures of the particular peptide. The four starting structures were then individually solvated in a truncated octahedron box of TIP4Pew[18] water with a buffer region of 15.0 Å using the tleap program of AmberTools19. The number of water molecules was further adjusted manually using in-house scripts to obtain the same number of atoms in all 4 boxes for each peptide. Since we analyzed three different peptides there was a total of 12 boxes. Each box was minimized and further heated up using the same strategy as in ref. [17d]. The runs were continued in the NPT ensemble for a short conventional molecular dynamics (cMD) production simulation of 75 ns. For each peptide, the last 50ns of each of the four independent cMDs were used to obtain parameters necessary for the corresponding accelerated molecular dynamics (aMD) simulations (i.e., the averaged total potential energy and the averaged dihedral potential energy). The structures from the last frames of each cMD were used as starting structures for the aMD runs which was performed for a period of 700 ns for every box. Parameters a1, a2, b1, and b2 of the boosting potential where taken as 3.5, 3.5, 0.175, and 0.175 kcal mol^{-1}, respectively (see ref. [20] for details and Table S3 for the boosting parameters used in this work). All aMD and cMD simulations were performed with a time step of 2 fs, periodic boundary

conditions and the obtained trajectories were saved every 20 ps. The SHAKE algorithm was applied to restrict all hydrogen containing bonds while long-range electrostatic interactions were calculated using the particle-mesh Ewald scheme as implemented in Amber18. Temperature and pressure were controlled with Langevin dynamics (collision frequency of 2.0 ps^{-1}) and isotropic position scaling, respectively.

Clustering and secondary structure analysis were performed on the last 300 ns of each aMD simulation using the cpptraj program of AmberTools19. Secondary structure analysis was additionally performed for the whole set of conformation in each structural cluster for each peptide. The average fraction of secondary structure for each amino acid were then summed up and normalize to yield the final representation as shown in Figure 3 of the main text. Clustering was done using the hierarchical agglomerative algorithm based on root mean square deviation (RMSD) of the backbone atoms (CA, C, and N) of the peptides, complete-linkage (using the maximum distance between members of two clusters) and requesting a total number of 20 clusters. Number of clusters was preferred over distance criterion due to the high flexibility of some of the peptides considered here.

RMSD of the representative structures of each cluster was calculated with respect to the active conformation of Axin's binding domain as found in the crystal structure with PDB ID 1QZ7[19] based on backbone atoms and disregarding the first three and the last two residues of the sequence due to the rather random conformation that this part of the Axin binding domain adopts in the crystal structure.

Table S3: Boosting parameters for accelerated molecular dynamics (energies are given in kcal mol^{-1}).

	peptide **P5**	peptide **P6**	peptide ***a*AxWt**
Number of residues	18	17	17
Number of atoms	12946	13790	13064
Average total potential energy	-35745.6	-38049.6	-36385.1
Average dihedral potential energy	222.9	210.2	209.5
Potential energy thershold	-33480.1	-35636.4	-34098.9
Dihedral potential energy thershold	285.9	269.7	269

References

[1] M. Frese, N. Sewald, *Angew. Chem. Int. Ed.* **2015**, *54*, 298.

[2] J. García-Pindado, T. Willemse, R. J. M. Goss, B. U. W. Maes, E. Giralt, S. Ballet, M. Teixidó, *Biopolymers* **2018**, *109*, e23112.

[3] A. Dumas, C. D. Spicer, Z. Gao, T. Takehana, Y. A. Lin, T. Yasukohchi, B. G. Davis, *Angew. Chem. Int. Ed.* **2013**, *52*, 3916 –3921.

[4] R. T. Raines, G. Ellis, M. Palte, Patent WO 2013/110005 A1.

[5] Novabiochem, *Catalog & Peptide Synthesis Handbook*, **1999**.

[6] E. Kaiser, R. L. Colescott, C. D. Bossinger, P. I. Cook, *Anal. Biochem.* **1970**, *34*, 595.

[7] Y.-W. Kim, T. N. Grossmann, G. L. Verdine, *Nat. Protoc.* **2011**, *6*, 761.

[8] A. Afonso, O. Cussó, L. Feliu, M. Planas, *Eur. J. Org. Chem.* **2012**, *2012*, 6204.

[8a] J. M. Scholtz, H. Qian, E. J. York, J. M. Stewart, R. L. Baldwin, *Biopolymers*, **1991**, *31*, 1463-1470.

[9] L. Dietrich, B. Rathmer, K. Ewan, T. Bange, S. Heinrichs, T. C. Dale, D. Schade, T. N. Grossmann, *Cell Chem. Biol.* **2017**, *24*, 958-968.e5.

[10] M. H. A. Roehrl, J. Y. Wang, G. Wagner, *Biochemistry* **2004**, *43*, 16056-16066.

[11] G. Lautrette, F. Touti, H. G. Lee, P. Dai, B. L. Pentelute, *J. Am. Chem. Soc.* **2016**, *138*, 8340.

[12] Gaussian 09, Revision A.02, M. J. Frisch, G. W. Trucks, H. B. Schlegel, G. E. Scuseria, M. A. Robb, J. R. Cheeseman, G. Scalmani, V. Barone, G. A. Petersson, H. Nakatsuji, X. Li, M. Caricato, A. Marenich, J. Bloino, B. G. Janesko, R. Gomperts, B. Mennucci, H. P. Hratchian, J. V. Ortiz, A. F. Izmaylov, J. L. Sonnenberg, D. Williams-Young, F. Ding, F. Lipparini, F. Egidi, J. Goings, B. Peng, A. Petrone, T. Henderson, D. Ranasinghe, V. G. Zakrzewski, J. Gao, N. Rega, G. Zheng, W. Liang, M. Hada, M. Ehara, K. Toyota, R. Fukuda, J. Hasegawa, M. Ishida, T. Nakajima, Y. Honda, O. Kitao, H. Nakai, T. Vreven, K. Throssell, J. A. Montgomery, Jr., J. E. Peralta, F. Ogliaro, M. Bearpark, J. J. Heyd, E. Brothers, K. N. Kudin, V. N. Staroverov, T. Keith, R. Kobayashi, J. Normand, K. Raghavachari, A. Rendell, J. C. Burant, S. S. Iyengar, J. Tomasi, M. Cossi, J. M. Millam, M. Klene, C. Adamo, R. Cammi, J. W. Ochterski, R. L. Martin, K. Morokuma, O. Farkas, J. B. Foresman, and D. J. Fox, Gaussian, Inc., Wallingford CT, 2016.

[13] D. A. Case, I. Y. Ben-Shalom, S. R. Brozell, D. S. Cerutti, T. E. Cheatham, III, V. W. D. Cruzeiro, T. A. Darden, R. E. Duke, D. Ghoreishi, M. K. Gilson, H. Gohlke, A. W. Goetz, D. Greene, R Harris, N. Homeyer, S. Izadi, A. Kovalenko, T. Kurtzman, T. S. Lee, S. LeGrand, P. Li, C. Lin, J. Liu, T. Luchko, R. Luo, D. J. Mermelstein, K. M. Merz, Y. Miao, G. Monard, C. Nguyen, H. Nguyen, I. Omelyan, A. Onufriev, F. Pan, R. Qi, D. R. Roe, A. Roitberg, C. Sagui, S. Schott-Verdugo, J. Shen, C. L. Simmerling, J. Smith, R. Salomon-Ferrer, J. Swails, R. C. Walker, J. Wang, H. Wei, R. M. Wolf, X. Wu, L. Xiao, D. M. York and P. A. Kollman (2018), AMBER 2018, University of California, San Francisco.

[14] F. Neese, *WIREs Comput. Mol. Sci.* **2012**, *2*, 73.

[15] J. A. Maier, C. Martinez, K. Kasavajhala, L. Wickstrom, K. E. Hauser, C. Simmerling, *J. Chem. Theory Comput.* **2015**, *11*, 3696.

[16] J. Wang, R. M. Wolf, J. W. Caldwell, P. A. Kollman, D. A. Case, *J. Comput. Chem.* **2004**, *25*, 1157.

[17] a) A. Marion, J. Góra, O. Kracker, T. Fröhr, R. Latajka, N. Sewald, I. Antes, *J. Chem. Inf. Model.* **2018**, *58*, 90. b) D. C. S. Schröder, O. Kracker, T. Fröhr, J. Góra, M. Jewginski, A. Nieß, I. Antes, R. Latajka, A. Marion, N. Sewald, *Front. Chem.* **2019**, *7*, 155. c) O. Kracker, J. Góra, J. Krzciuk-Gula, A. Marion, B. Neumann, H.-G. Stammler, A. Nieß, I. Antes, R. Latajka, N. Sewald, *Chem. Eur. J.* **2018**, *24*, 953. d) I. Kemker, C. Schnepel, D. C. Schröder, A. Marion, N. Sewald, *J. Med. Chem.* **2019**, *62*, 7417.

[18] H. W. Horn, W. C. Swope, J. W. Pitera, J. D. Madura, T. J. Dick, G. L. Hura, T. Head-Gordon, *J. Chem. Phys.* **2004**, *120*, 9665.

[19] Y. Xing, W. K. Clements, D. Kimelman, W. Xu, *Genes Dev.* **2003**, *17*, 2753.

[20] Y. Miao, F. Feixas, C. Eun, J. A. McCammon, *J. Comput. Chem.* **2015**, *36*, 1536.
